# Secrets of Soil Survival Revealed by the Genome Sequence of Arthrobacter aurescens TC1

**DOI:** 10.1371/journal.pgen.0020214

**Published:** 2006-12-22

**Authors:** Emmanuel F Mongodin, Nir Shapir, Sean C Daugherty, Robert T DeBoy, Joanne B Emerson, Alla Shvartzbeyn, Diana Radune, Jessica Vamathevan, Florenta Riggs, Viktoria Grinberg, Hoda Khouri, Lawrence P Wackett, Karen E Nelson, Michael J Sadowsky

**Affiliations:** 1 The Institute for Genomic Research, Rockville, Maryland, United States of America; 2 The BioTechnology Institute, University of Minnesota, St. Paul, Minnesota, United States of America; 3 Microbial and Plant Genomics Institute, University of Minnesota, St. Paul, Minnesota, United States of America; Department of Energy Joint Genome Institute, United States of America

## Abstract

Arthrobacter sp. strains are among the most frequently isolated, indigenous, aerobic bacterial genera found in soils. Member of the genus are metabolically and ecologically diverse and have the ability to survive in environmentally harsh conditions for extended periods of time. The genome of Arthrobacter aurescens strain TC1, which was originally isolated from soil at an atrazine spill site, is composed of a single 4,597,686 basepair (bp) circular chromosome and two circular plasmids, pTC1 and pTC2, which are 408,237 bp and 300,725 bp, respectively. Over 66% of the 4,702 open reading frames (ORFs) present in the TC1 genome could be assigned a putative function, and 13.2% (623 genes) appear to be unique to this bacterium, suggesting niche specialization. The genome of TC1 is most similar to that of *Tropheryma, Leifsonia, Streptomyces,* and *Corynebacterium glutamicum,* and analyses suggest that A. aurescens TC1 has expanded its metabolic abilities by relying on the duplication of catabolic genes and by funneling metabolic intermediates generated by plasmid-borne genes to chromosomally encoded pathways. The data presented here suggest that *Arthrobacter*'s environmental prevalence may be due to its ability to survive under stressful conditions induced by starvation, ionizing radiation, oxygen radicals, and toxic chemicals.

## Synopsis

Soil systems contain the greatest diversity of microorganisms on earth, with 5,000–10,000 species of microorganism per gram of soil. Arthrobacter sp. strains have a primitive life cycle and are among the most frequently isolated, indigenous soil bacteria, found in common and deep subsurface soils, arctic ice, and environments contaminated with industrial chemicals and radioactive materials. To better understand how these bacteria survive in environmentally harsh conditions, the authors used a structural genomics approach to identify genes involved in soil survival of Arthrobacter aurescens strain TC1, a bacterium originally isolated for its ability to degrade the herbicide atrazine. They found that the genome of this bacterium comprises a single circular chromosome and two plasmids that encode for a large number proteins involved in stress responses due to starvation, desiccation, oxygen radicals, and toxic chemicals. *A. aurescens'* metabolic versatility is in part due to the presence of duplicated catabolic genes and its ability to funnel plasmid-derived intermediates into chromosomally encoded pathways. *Arthrobacter*'s array of genes that allow for survival in stressful conditions and its ability to produce a temperature-tolerant “cyst”-like resting cell render this soil microorganism able to survive and prosper in a variety of environmental conditions.

## Introduction

Strains of *Arthrobacter* species were first culled from soils in the 19th century [1] and are among the most frequently isolated, indigenous, aerobic bacterial genera found in soils [2–6]. These bacteria typically appear as Gram-negative rods in younger cultures and as Gram-positive cocci in older cultures. The molecular basis for their distinct method of growth is not known. Due to their pleomorphic and heterogeneous appearances, Arthrobacter sp. strains were originally grouped with the Corynebacteria [7]. However, more modern systematic analyses indicate that members of the genus *Arthrobacter* are taxonomically clustered with the Micrococcaceae, which is comprised of high G+C, Gram-positive bacteria of the genera *Citrococcus, Kocuria, Micrococcus, Renibacterium, Nesterenkonia,* and *Rothia* [8].

Arthrobacter sp. are ubiquitous and have been found in common soils and in extreme environments, such as the deep subsurface, arctic ice, chemically contaminated sites, and radioactive environments [9–13]. Arthrobacter sp. strains were reported to be among the most prevalent genera of bacteria isolated from beneath leaking radionuclide storage tanks at the Department of Energy facility in Hanford, Washington, United States [14].

The environmental prevalence of *Arthrobacter* may be due, in part, to its ability to survive long periods under stressful conditions induced by starvation, temperature shifts, ionizing radiation, oxygen radicals, and toxic chemicals [15–19]. This remarkable survival ability is exemplified by the recovery of Arthrobacter sp. from desert Antarctic soils following 3 y of drying [20]; experiments in the laboratory confirm these observations [21–23]. In these studies, morphogenesis of *Arthrobacter* from rod to coccus has been implicated in the bacterium's ability to survive stresses, with the small coccoid-like state described as the most stable form. The transition to this coccoid-like state has been demonstrated to require manganese [22], and accumulation of this metal in the bacterial cytoplasm has been linked to radiation-stress survival in Deinococcus radiodurans and other bacteria [24].

Arthrobacter sp. are metabolically diverse and have been isolated for their ability to biodegrade a variety of environmental pollutants such as glyphosate, methyl tert-butyl ether, 2,4-dichlorophenoxyacetate (2,4-D), nictotine, 4-nitrophenol, dimethylsilanediol, endoxohexahydrophthalate (endothal), fluorene, phthalate, nitroglycerine, and a very large number of *s*-triazine herbicides. *Arthrobacter* have also been shown to be highly resistant to some toxic heavy metals and chromate anion [25–31]. Arthrobacter aurescens strain TC1 (originally isolated from soil at a South Dakota spill site containing 1,000 lb of the herbicide atrazine [30]) has been shown to metabolize over 23 different *s*-triazine compounds [31], the greatest number of *s*-triazine compounds catabolized by a single organism thus far reported. Moreover, metabolic and genomic analyses suggest that A. aurescens TC1 has the capacity to catabolize over 500 structurally diverse *s*-triazine compounds [45].

The molecular basis for *Arthrobacter's* success in surviving stress conditions in soil and metabolizing diverse compounds has been investigated only sporadically. Such studies included the isolation of genes involved in glycine betaine synthesis in A. globiformis [32,33], the analysis of trehalose and glycogen synthesis under stress conditions in A. globiformis [34], the sequencing of the nicotine-degradation plasmid in A. nicotinovorans [35], and the partial sequencing of the genome of the heavy-metal resistant Arthrobacter sp. strain FB24 (http://genome.jgi-psf.org/draft_microbes/artf/art_f.home.html).

In this report we describe the complete sequencing, assembly, and annotation of the genome of A. aurescens TC1. The A. aurescens genome consists of a chromosome and two plasmids. Genomic analyses provide new insights into this versatile and autochthonous bacterium's ecological niche and survival strategies in soils.

## Results/Discussion

### Genome Features of A. aurescens TC1

#### General genome features.

**Figure 1:**
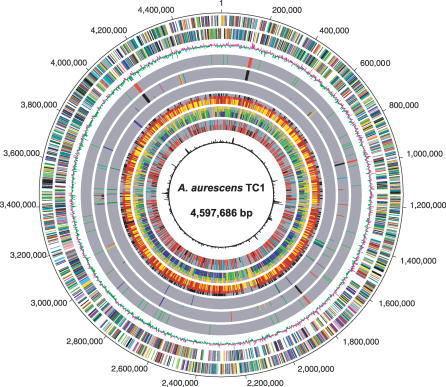
Circular Representation of the Chromosome of A. aurescens TC1 Each concentric circle is numbered from the outermost circle to the inner most circle and represents genomic data for A. aurescens strain TC1 chromosome. The first and second circles represent the predicted coding sequences on the plus and minus strands, respectively, colored by functional role categories: salmon, amino acid biosynthesis; light blue, biosynthesis of cofactors and prosthetic groups and carriers; light green, cell envelope; red, cellular processes; brown, central intermediary metabolism; yellow, DNA metabolism; green, energy metabolism; purple, fatty acid and phospholipid metabolism; pink, protein fate and synthesis; orange, purines, pyrimidines, nucleosides, and nucleotides; blue, regulatory functions; grey, transcription; teal, transport and binding proteins; and black, hypothetical and conserved hypothetical proteins. The third circle displays the G + C skew: positive G + C skew in magenta and negative G + C skew in green. The fourth circle displays the rRNAs (red), sRNAs (blue), and tRNAs (green). The fifth circle displays repeated sequences of at least 50 bp long (at least 97% identity between two repeats); each color/tick size represents a different repeat. Prophage (blue ticks) and transposon (dark green ticks) genes are displayed on the sixth circle. The seventh circle displays the percentage of similarity (BLASTP searches) between TC1 and Arthrobacter sp. FB24 ORFs: >95%, full-sized black ticks; 85%–95%, three-quarter sized brown ticks; 75%–85%, three-quarter sized red ticks; 65%–75%, half-sized gold ticks; 55%–65%, half-sized yellow ticks. The eighth and ninth circles display the organism best match: L. xili (blue ticks), S. coelicolor (green ticks), and S. avermitilis (gold ticks) on circle 8. N. farcinica (red ticks), T. fusca (brown ticks), M. avium (cyan ticks), and C. efficiens (black ticks) on circle 9. The tenth circle shows the regions of atypical composition (χ^2^ analysis).

**Figure 2:**
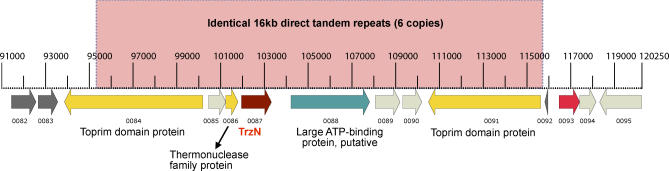
Organization of the Region of the A. aurescens pTC1 Plasmid Containing Six Identical Direct Tandem Repeats of Approximately 16 kb in Length For clarity, the locus tag (AAur_pTC1) was removed from the ORF numbers (for example, ORF number 0082 is AAur_pTC10082).

The genome of A. aurescens TC1 is comprised of three molecules: a single circular chromosome of 4,597,686 bp (locus tag: AAur) and two plasmids: pTC1 (locus tag: AAur_pTC1) and pTC2 (locus tag: AAur_pTC2) of 328,237 and 300,725 bp, respectively (Figure 1; Figure 2; Table 1). Since the pTC1 contains six identical copies of a 16-kb repeat region, the final molecule size is approximately 408 kb (see below and Materials and Methods). Overall, the chromosome and plasmids of the A. aurescens genome contain 4,708 open reading frames (ORFs), of which 3,071 (65.2%) could be assigned a putative function. Approximately 13.2% (623 hypothetical proteins) of the A. aurescens TC1 genome appears to be unique to this bacterium, with no matches to any known sequence.

**Table 1:**
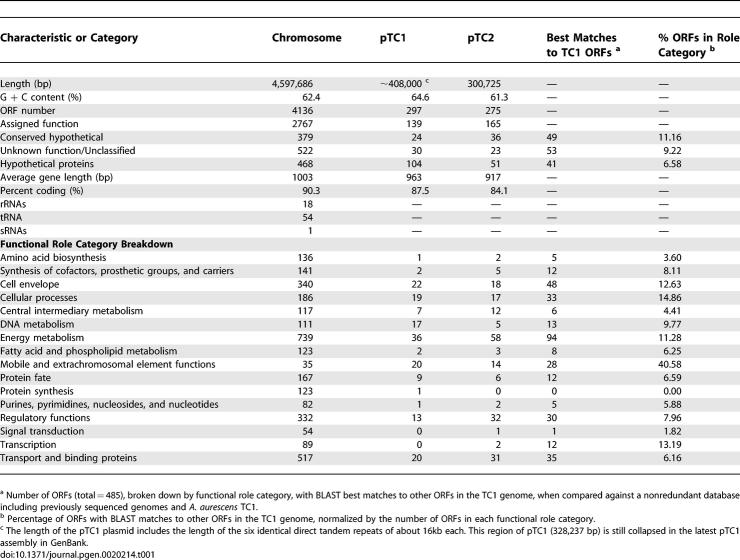
General Features of A. aurescens TC1 Genome

A total of 485 genes (10.3%) have their best BLAST matches to other A. aurescens TC1 genes, rather than to genes outside the genome of strain TC1 (Table 1), indicating a high degree of genome duplication and possible functional redundancy. This redundancy may allow strain TC1 to rapidly adapt to changing environments. A list of these genes, with their best match, is provided in Table S1. Not surprisingly, the largest fraction of these recently duplicated genes consists of transposase genes (see below): 40% of the TC1 transposase genes have their best match within the TC1 genome. Functional role categories that appear to have undergone extensive gene duplication include genes involved in cellular processes (14.8%), transcription (13.2%), cell envelope (12.6%), and energy metabolism (11.3%). Of these ORFs, 30 encode transcriptional regulators, including those in the Gnt, Ars, Lux, and Mar family, and 109 encode proteins involved in metabolism (central metabolism and the metabolism of aldehydes, alcohols, and other substrates). Interestingly, 25 of these genes appear to be involved in resistance to heavy metals or stresses, and four encode for RNA polymerase σ^70^ factor, which is involved in the phosphate starvation response [36].

Gene duplications (paralogs) have been postulated to assist microbes in adapting to changing environments. Since A. aurescens lives in soil, a habitat of constant change, such duplications most likely facilitate the survival of strain TC1. Overall, our results are in agreement with Gevers and colleagues [37], who reported that functional classification of paralogs in 106 microbial genomes revealed a preferential enrichment for genes involved in transcription, metabolism, and other defense mechanisms.

The A. aurescens TC1 chromosome contains 11 genomic islands (of 5 kb or larger) encoding 180 genes that have atypical G+C content and nucleotide composition when compared to the rest of the TC1 genome (Table S2). The islands include transposons and related genes, transcriptional regulators, resistance genes, and genes involved in metabolism and transport of a wide range of substrates. Genes displaying atypical composition have also been detected in the genomes of other soil organisms, like Pseudomonas putida KT2440 [38], and have been postulated to contribute to saprophytic competence and survival as K-strategists, which devote more energy to competitive success and survival than to reproduction [39]. Moreover, these genes are thought to arise from horizontally transferred islands, noncoding sequences, and ancient, conserved gene clusters [40]. In P. putida, about 20% of the genes contain a genomic signature that is different from the rest of the 6.2-Mb genome [38,39], whereas A. aurescens TC1 contains about 10-fold less. This suggests that the A. aurescens TC1 genome may be more stable than that of P. putida, or that the genome of the latter bacterium is more mosaic than that of the former. Twenty-nine out of 105 islands with atypical composition in P. putida are thought to have been acquired by horizontal gene transfer through mobile genetic elements, many of which may contribute to this organism's extensive metabolic abilities [45]. In contrast, A. aurescens TC1 appears to have expanded its metabolic abilities by relying more on gene duplication than on horizontal gene transfer and by funneling metabolic intermediates generated by plasmid-borne genes to chromosomally encoded pathways. Out of the 11 genomic islands with atypical composition that are potentially contributing to this organism's metabolic proficiency, only two are associated with mobile genetic elements. Region 4 contains two degenerate/truncated IS256 family transposases, three Tn554-related transposases, an ISAau1 element, a degenerate IS110 family transposase, a Tn3-family transposase, and two phage integrase family domain proteins. Region 6 contains two copies of the ISAau1 element.

Consistent with the majority of high G + C Gram-positive bacteria, the A. aurescens TC1 genome does not contain genes for the synthesis or hydrolysis of polyhydroxybutyrate, which appears mostly restricted to members of the Proteobacteria [41]. In addition, A. aurescens TC1 does not contain genes for flagella synthesis or motility. Lack of motility in this bacterium is characteristic of this species group and many species within the genus. Concomitantly, A. aurescens TC1 also lacks genes for chemotaxis. In contrast, other soil microbes like P. putida KT2440 have a repertoire of genes for motility, flagella, and chemotaxis [38]. Consequently, the competitiveness, survival ability, and metabolic versatility of A. aurescens TC1 apparently does not require movement of this bacterium, which presumably remains attached to soil particles or soil organic matter.

A. aurescens TC1 also appears to be ecologically versatile and capable of growing on a wide variety of carbon compounds. Moreover, based on gene assignments to the “Energy Metabolism” functional role category of The Institute for Genomic Research (TIGR) (http://cmr.tigr.org/tigr-scripts/CMR/RoleIds.cgi), 17.7% of the TC1 genome (833 ORFs) is devoted to energy production. This is in contrast to many sequenced organisms in which approximately 4%–7% of genes are involved in energy production and conversion [42]. Consistent with the extensive metabolic versatility associated with the degradation of *s*-triazines and other compounds, TC1 encodes 568 putative transporters and binding proteins (12.06% of the TC1 genome): 101 for amines, peptides, and amino acids, and 107 for carbohydrates, alcohols, and acids. This is comparable to the genome of P. putida KT2440, which, when sequenced in 2002 [38], had the highest number of predicted putative transporters and binding proteins (671, 12.38% of the genome) of any sequenced bacterium. Interestingly, A. aurescens TC1 contains three predicted ABC family opine transporters (AAur_0594, AAur_2744, and AAur_3735), suggesting that TC1 may have the ability to degrade plant-derived opines or other novel amino acid–derived compounds produced in the plant rhizosphere [43].

#### Chromosomal insertion sequence elements.

A. aurescens TC1 contains a total of 46 ORFs encoding functions consistent with transposons or insertion sequence (IS) elements, 23 of which are on the chromosome. No phage could be identified in the TC1 genome. Compared to other sequenced soil microorganisms, such as P. putida KT2440, strain TC1 has relatively few IS elements and transposons. The most abundant IS is represented by 11 perfect copies of a previously undescribed ISAau1 element. All 11 copies are flanked by unique 4-bp direct repeats; eight, two, and one copies were localized to the chromosome, pTC1, and pTC2, respectively. This element belongs to the IS407 group of the IS3 family of transposases. Since all copies of this new IS element are perfectly identical, it suggests that they were acquired relatively recently by the A. aurescens TC1 genome. However, at least two copies of ISAau1 interrupt chromosomal genes (AAur_1382/AAur_1385, encoding tyramine oxidase; and AAur_3174/AAur_3176, an acyltransferase family protein), and one copy interrupts a pTC1-encoded putative membrane protein (AAur_pTC10101/AAur_ pTC10103). IS407 elements have previously been reported to be present in other environmental bacteria, and extensive characterization has mainly been done in Burkholderia cepacia strains where this IS element has been shown to activate gene expression via a σ^70^-dependent promoter [44].

### A. aurescens TC1 Plasmid Features

#### A. aurescens pTC1 plasmid.

A. aurescens strain TC1 contains two plasmids, pTC1 and pTC2 (Figure 2). The pTC1 plasmid, is 328,237 bp in size (not including the six identical copies of a 16-kb repeat region; see below and Materials and Methods), contains 297 ORFs, and has a G + C content of 64.6%, a value slightly greater than that of the chromosome. The pTC1 plasmid contains genes involved in the biodegradation of atrazine to cyanuric acid. More interestingly, a portion of pTC1 contains six identical direct tandem repeats of about 16 kb, beginning at around coordinate 95000 (AAur_pTC10084, at nucleotide position 100190–93861) and ending approximately 183 bp upstream of the start of AAur_pTC10091 (nucleotide position: 115622–110496) (Figure 2). This repeat region includes the triazine hydrolase gene, *trzN* (AAur_pTC10087), and an exact gene duplication of a toprim domain protein (AAur_pTC10084 and AAur_pTC10091) (Figure 2), a conserved region from DNA primase, corresponding to the topoisomerase–primase domain common to DnaG primases, topoisomerases, and the RecR/M DNA repair proteins. Multiple copies of *trzN*, the first gene in the *s*-triazine biodegradation pathway, may have important consequences for this bacterium's ability to catabolize a large number of *s*-triazine compounds as the sole nitrogen and carbon sources for growth [45]. Redundancy in *trzN* may enhance catabolism via gene dosage effects, or provide a competitive advantage to this bacterium versus organisms such as Pseudomonas sp. strain ADP, which contains a single triazine hydrolase gene that may be more readily lost in growth conditions lacking adequate selection pressure.

**Figure 3:**
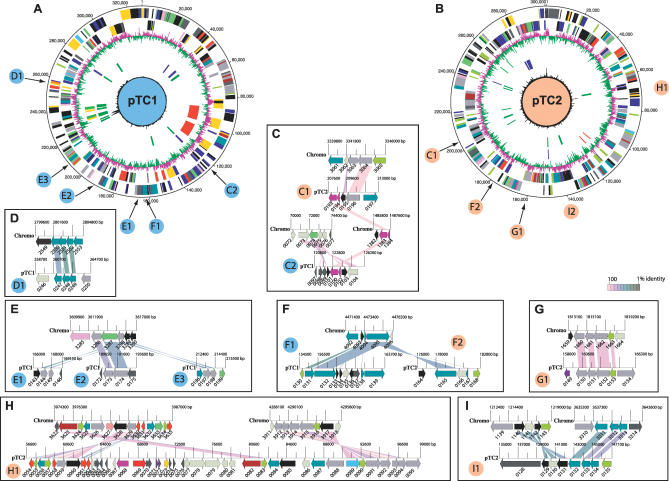
Circular Representations of the pTC1 and pTC2 Plasmids of A. aurescens TC1, and Comparative Linear Displays of Some of the Plasmid Regions Shared with the Strain TC1 Chromosome (A and B) Each concentric circle of the circular figures is numbered from the outermost circle to the innermost circle. For each plasmid, the first and second circles represent the predicted coding sequences on the plus and minus strands, respectively, colored by functional role categories (see Figure 1). The third circle displays the G + C skew: positive G + C skew in magenta and negative G + C skew in green. The fourth circle displays the repeated sequences of at least 50 bp long (at least 97% identity between two repeats); each color/tick size represents a different repeat. Prophage (blue ticks) and transposon (dark green ticks) genes are displayed on the fifth circle. The sixth circle shows the regions of atypical composition (χ^2^ analysis). (C–I) Comparative linear displays of some of the pTC1 and pTC2 sequences matching the TC1 chromosome. The percent of protein identity is indicated by the color of the connecting lines (legend on the right side of the figure). For clarity, the locus tags (AAur_ for the chromosome, and AAur_pTC1 and AAur_pTC2 for the pTC1 and pTC2 plasmids, respectively) were removed from the ORF numbers. For example, the chromosomal ORF number 2549 is AAur_2549, the pTC1 ORF number 0246 is AAur_pTC10246, and the pTC2 ORF number 0054 is AAur_pTC20054.

A cassette of four genes of unknown function is found both on the chromosome (AAur_0073 to AAur_0076) and on pTC1 (AAur_pTC10098, AAur_pTC10099, AAur_pTC10101/AAur_pTC10103, and AAur_pTC10104) (Figure 3C2; Table S3). While gene arrangement is identical in these two cassettes, the genes are not identical to each other, sharing between 82% and 93% identity at the protein level. One plasmid-borne gene (AAur_pTC10101/AAur_pTC10103) also differs due to disruption by an IS element (AAur_pTC10100 and AAur_pTC10102), while the chromosomal version appears intact (Figure 3C2). The gene cassette in pTC1 could have originated from the chromosome of the same strain (the sequence differences between the chromosome and plasmid ORFs could be the consequence of the fast mutation rate of evolving new functions), or, most likely, it originated from the chromosome of a different strain. It will be interesting to test whether these genes of unknown function, in pTC1 and the chromosome, might be beneficial to this organism.

Among the other genes shared between the chromosome and plasmid pTC1 are five genes related to cytochrome *c*: three cytochrome *c* biosynthesis genes (AAur_pTC10174 and two *ccdA* genes encoded by AAur_pTC10144 and AAur_pTC10197) (Figure 3E), one putative cytochrome *c* assembly protein (AAur_pTC10191), and one cytochrome *c* oxidase subunit III (AAur_pTC10179). The CcdA protein is also found on pTC2 (AAur_pTC20033 and AAur_pTC20039); all the CcdA proteins encoded by the pTC1 and pTC2 plasmids are homologs of the same chromosomal *ccdA* gene (AAur_3288). One additional putative cytochrome *c* biogenesis protein was found on the pTC2 plasmid (AAur_pTC20039). Both the pTC1- (AAur_pTC10174) and pTC2-encoded (AAur_pTC20039) ORFs are highly similar (61.5% and 72.7% identity, respectively) to the corresponding chromosomal ORF (AAur_3287; Table S3). In addition, strain TC1 contains five and seven chromosome- and plasmid pTC1–borne genes encoding resistance to cobalt–zinc–cadmium and copper, respectively.

A. aurescens strain TC1 was originally isolated by its ability to degrade atrazine [31]. Preliminary studies showed that the three atrazine catabolism genes *trzN*, *atzB,* and *atzC* were present on a 160-kb region of the largest plasmid [46]; the complete genomic sequence presented here is consistent with these previous findings. All three triazine hydrolase genes, *trzN*, *atzB*, and *atzC* (AAur_pTC10087, AAur_pTC10218, and AAur_pTC10212, respectively), were located on plasmid pTC1 and nowhere else in the A. aurescens TC1 genome. A complete cluster of genes involved in the biodegradation of isopropylamine *(ipu)* was found on each of the two TC1 plasmids, pTC1 (∼14-kb region delimited by AAur_pTC10058 and AAur_pTC10069) and pTC2 (∼16-kb region delimited by AAur_pTC20219 and AAur_pTC20208). They most likely allow A. aurescens TC1 to metabolize several *s*-triazines as a sole carbon and nitrogen source for growth. The TC1 *ipu* genes are highly homologous to several of those in the *ipu* gene cluster previously reported to be involved in isopropylamine catabolism by Pseudomonas sp. strain KIE171 [47]. However, unlike the KIE171 *ipu* genes, which are clustered in an operon-like fashion, the pTC1 and pTC2 *ipu* genes are clustered, but do not appear to be organized in one single operon.

Fourteen ORFs on pTC1 are consistent with transposons and/or IS elements, including the previously identified IS elements IS1071 (Tn3 family element) and ISPps1 (IS91 family element). The IS1071 was previously shown to be located adjacent to the atrazine degradation genes *atzA* and *atzB* on plasmid pADP-1 in *Pseudomonas* strain ADP [48]. In addition, pTC1 harbors transposases belonging to the IS3 (ISAau1), IS5, IS21, IS110, and IS1380 families. In contrast, the chromosome contains only ISAau1 (eight copies), a Tn3 family element that is not IS1071, an element related to Tn554, and two degenerate IS110 and IS256 family transposases.

#### Relationship between pTC1-localized genes to sequences present on plasmids in other microorganisms.

**Figure 4:**
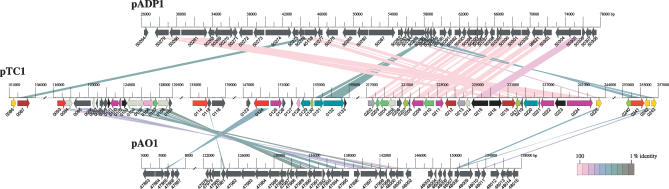
Comparative Linear Display Representing the Sequence Homologies between the A. aurescens pTC1 Plasmid, the Pseudomonas sp. pADP-1 Plasmid, and the A. nicotinivorans pAO1 Plasmid Only selected regions for each of the three plasmids are shown. The percent of protein identity is indicated by the color of the connecting lines (legend on the bottom left side of the figure). For clarity, the locus tags (AAur_pTC1 for the pTC1 plasmid, AAK for the pADP-1 plasmid, and CAD for the pAO1 plasmid) were removed from the ORF numbers.

Genes on pTC1 showed limited homology to those on other sequenced plasmids, including plasmid sequences reported for Arthrobacter sp. FB24. The genes on pTC1 involved in atrazine degradation were initially discovered by homology to genes carried by plasmid pADP-1 from Pseudomonas sp. strain ADP [46]. The greatest relationship between pADP1 and pTC1, with an amino-acid similarity of 83.3%–100%, seems to be limited to the region delimited by 17 pTC1-encoded ORFS (AAur_pTC10202 through AAur_pTC10225; Table S4) containing the atrazine degradation genes *atzB* and *atzC* and several transposases (Figure 4). Outside this region, there were 14 additional genes showing more limited similarity (30%–43%) between plasmids pTC1 and pADP1, primarily encoding transposases, IS elements, and mercury-resistance proteins. However, two additional ORFs (AAur_pTC10210 and AAur_pTC10215) had significant amino acid similarity (81%–100%) to ORFs on pADP-1 encoding a putative transporter and a dihydrolipoamide dehydrogenase homolog, respectively (Table S4).

Twenty-six genes present on pTC1 also displayed significant amino-acid similarity (cutoff value ≥30%) to those on plasmid pAO1 from A. nicotinovorans [35] (Figure 4; Table S4). Among known proteins, the greatest similarity (88%) between the two plasmids was found in AAur_pTC10093, which endodes a putative Soj/ParA family protein, AAur_pTC10124 (51%), which encodes a putative ParB-partitioning protein, and AAur_pTC10243 (70%), which encodes a DNA-invertase (a site-specific recombinase/resolvase family protein). This suggests that genes involved in plasmid partitioning in pTC1 and pAO1 most likely share a common ancestor.

#### A. aurescens pTC2 plasmid.

The pTC2 plasmid, which contains 275 ORFs (Table 1), has a G + C content of 61.3%, the lowest of three replicons in this bacterium. It contains a large number of ORFS, encoding proteins with functions involved in the metabolism of nitrogenous compounds, energy metabolism, and transcriptional regulators, along with nine ORFs encoding functions consistent with transposons and/or IS elements. The plasmid pTC2 contains IS3 (ISAau1), IS110, and IS256 family transposases, and a Tn3 family resolvase. In addition, plasmid pTC2 contains three, four, and one ORFs involved in resistance to copper, arsenate, and cobalt–zinc–cadmium, respectively. Similar to the other plasmid, the pTC2 also contains an *ipuC* homolog encoding γ-glutamylisopropylamide synthetase, and other genes involved in the degradation of isopropylamine, which is also released during the degradation of *s*-triazine compounds. Plasmid pTC2 contains 111 ORFs with significant amino-acid identity to chromosomally-encoded proteins (Table S3). The sharing of nearly identical genes on plasmids and the chromosome in the same organism has previously been reported for soil [49,50] and other bacteria [51,52], and it is tempting to speculate that plasmid-encoded functions may allow for competitive success in the environment. While the origin of the redundant genes is unknown, they may have arisen from transposition events occurring between plasmids and the chromosome or via horizontal gene transfer, especially for redundant homologs (or paralogs) that have significant differences at the amino-acid level. Genes present on pTC2 have no significant identity to translated plasmid-localized ORFs reported for Arthrobacter sp. FB24.

### Comparative Genomics

#### Comparison of the genomes *of A. aurescens* TC1 and Arthrobacter sp. FB24.

The closed genome sequence of Arthrobacter sp. strain FB24, which was isolated from chromate- and xylene-enriched soil microcosms, was produced by the United States Department of Energy Joint Genome Institute (http://genome.jgi-psf.org/draft_microbes/art_f/art_f.download.ftp.html). The FB24 genome released by DOE/JGI consists of four molecules: a 4.7-Mb chromosome and three plasmids, with sizes of 96.5 kb, 115.5 kb, and 159.5 kb. The FB24 genome has an overall estimated G + C content of 65.4%, slightly greater than that of A. aurescens TC1 (62.4%). The FB24 genome has a G + C content of 65.5% for the chromosome and 64.6%, 63.3%, and 65.0% for each of the three plasmids, respectively. A computer-only (i.e., no manual curation) annotation of the four FB24 molecules using the TIGR annotation pipeline predicted a total number of 4,702 ORFs: 4,313 for the chromosome, 105 for the 96.5-kb plasmid, 116 for the 115.5-kb plasmid, and 168 for the 159.5-kb plasmid.

Whole genome nucleotide and amino-acid alignments between the TC1 and FB24 genomes (Figure S1) show an overall conservation of synteny between the chromosomes of the two organisms, with an overall similarity of 79.09% at the amino-acid level. Out of 4,136 ORFs comprising the TC1 chromosome, 540 TC1 ORFs (13.08%) do not have an equivalent in the FB24 genome (BLASTP e-value cutoff of 10^−5^, corresponding to amino-acid level of similarity ≥35%) (Table S5). The remaining 3,596 TC1 ORFs could be mapped to the FB24 chromosome, with a percentage of amino-acid similarity ranging from 37.1% to 100%; 25 proteins are 100% identical between TC1 and FB24; of these 25, ten are ribosomal proteins. The largest cluster of genes unique to A. aurescens TC1, i.e., absent from the FB24 genome (“gaps” in the dot-plot in Figure S1), is a region spanning 250 kb of the TC1 chromosome. An overwhelming majority of the A. aurescens TC1 unique genes encode hypothetical proteins (237 proteins, 43.88% of the total set of unique proteins) (Table S4), conserved hypothetical proteins, conserved domain proteins (76 proteins, 14.07% of total set of unique proteins), or proteins of unknown function (nine proteins). The A. aurescens TC1 chromosome also encodes 38 integral membrane proteins that are not found in the FB24 genome, as well as 15 lipoproteins, 22 ISAau1-related proteins (11 transposase orfA and 11 transposase orfB proteins), and eight transcriptional regulators, four of which belong to the AraC family. Finally, among the unique TC1 proteins that are important for the ability of A. aurescens TC1 to survive in the soil are a manganese-containing catalase (AAur_0634), a putative cobalt–zinc–cadmium efflux permease (AAur_3137), a putative cold shock protein (AAur_2005), and two proteins containing a cupin domain (AAur_3146 and AAur_4032).

#### Comparison with phylogenetically related bacteria.

**Figure 5:**
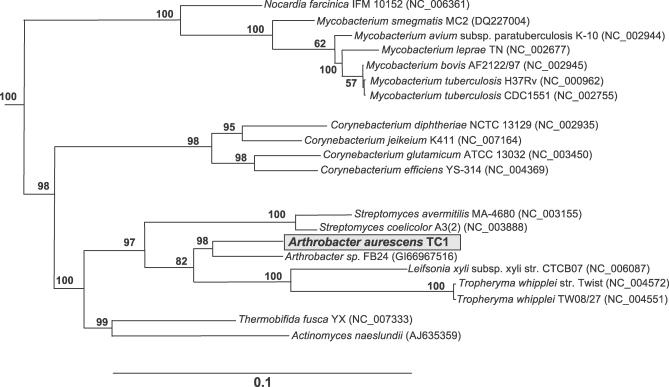
Maximum Likelihood Bootstrap Majority-Rule Consensus Tree for 16S rRNA Gene Sequences from 19 Strains Phylogenetically Related to A. aurescens TC1 Numbers adjacent to branch points are bootstrap percentages (*n* = 100 replicates). The bar represents 10% sequence divergence.

In agreement with phylogeny based on analysis of 16S rRNA (Figure 5), the genome of A. aurescens TC1 shares coding sequences, (>40% amino acid–sequence identity) with Streptomyces coelicolor A3(2) (668 genes, 14.2% of the TC1 genome) and Leifsonia xyli subsp. xyli str. CTCB07 (232 genes, 4.9% of the TC1 genome) (Figure S2). However, A. aurescens TC1 contains a large number of unique ORFs (3,413) relative to these bacteria, suggesting that this bacterium has diverged from its phylogenetic neighbors. Overall, genome comparisons with respect to genes involved in survival reflect, to some degree, the lifestyle of each organism. For example, the intracellular pathogens *Tropheryma* and *Leifsonia* have relatively few genes (three and 38 genes, respectively; Table 2) involved in stress responses, while the bacteria that live in soil, S. avermitilis (147 genes), P. putida (68 genes), A. aurescens TC1 (112 genes), and Arthobacter sp. FB24 (113 genes) have a relatively large number of genes encoding stress-related proteins. Interestingly, however, the industrially important soil bacterium Corynebacterium glutamicum has relatively few genes (39 genes) involved in survival in response to oxidative damage and other stresses (Table 2). As expected, organisms that have both a soil- and animal-host phase, such as the pathogenic Mycobacterium sp. strains, have a number of stress-related ORFs that are intermediate between these two extremes.

**Table 2:**
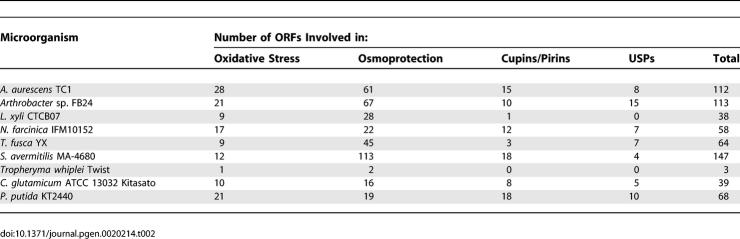
Comparison of the Number of Stress-Response Genes Found in the Genomes of A. aurescens TC1 and Other Related Bacteria

### Genome-Enabled Survival Strategies

Induction of alternative σ factors is an important strategy for coping with environmental stress in bacteria, and there is an apparent correlation between the number of alternative σ factors and the complexity of the environment, which synchronously induces genes in response to a particular stress. While Mycoplasma sp. strains (obligate intracellular pathogens) only contain the housekeeping σ and no alternative σ factors, Escherichia coli and S. coelicolor have six and 62 alternative σ factors, respectively. A. aurescens TC1 appears particularly poised for stress, encoding 17 σ70 family σ factors and one RNA polymerase σ70 factor RpoD (AAur_1761). Overall, the TC1 chromosome and the pTC2 plasmid encode 34 transcription factors, all but one (AAur_pTC20242) of which is located on the chromosome. In contrast, the genome of P. putida KT2440 encodes for 30 transcription factors, of which 18 belong to the σ70 family of σ factors. While the number of one- and two-component regulators is related to genome size, organisms with complex lifestyles or that need to contend with fluctuations in environmental conditions have been reported to have a disproportionate number of regulatory genes [53]. The genome of strain TC1 contains 331 ORFs encoding regulatory proteins, and TetR (44 proteins), MarR (25 proteins), LysR (20 proteins), IclR (17 proteins), and AraC (16 proteins) comprise the largest class of one-component systems.

#### Surviving constant internal oxidative stress.

One clue to the survival capacity of *Arthrobacter* is its ability to survive continuously generated reactive oxygen radicals produced by its intense aerobic metabolism. This derives, in part, from 14 genes encoding oxidases that use molecular oxygen to metabolize amino groups (EC numbers 1.4.3.- and 1.5.3.-; Table S6). This is more than any other bacterium for which a genome sequence has been published. Our analyses of the genomes of M. tuberculosis 210, Bacillus subtilis BS0001, M. avium 104, P. putida KT2440, *S. avermitilis* MA-4680, and *S. coelicolor A3(2)* revealed only ten, six, eight, seven, two, and three amine oxidases, respectively. Moreover, there are over 30 other oxidase genes in the TC1 genome (Table S6). Oxidases generate H_2_O_2_ that can generate other reactive oxygen species, such as hyperreactive hydroxyl radical, which can cause extensive damage leading to cell death. To this end, the genome of A. aurescens TC1 contains one superoxide dismutase gene, *sodA* (AAur_2087), four catalase genes (a manganese-containing catalase [AAur_0634], an organic halide–resistance protein *ohr* [AAur_1251], and two iron catalases [AAur_1864 and AAur_3059]), and an uncharacterized peroxidase-related enzyme (AAur_2025). While A. aurescens contains a SoxR homolog (AAur_3550), which may play a regulatory role in resistance to oxidative stress, no SoxS homolog is present. The lack of SoxS has been reported in many other environmental bacteria [54], suggesting that SoxR, which is induced by H_2_O_2_ and other superoxide compounds, directly interacts with other proteins to control expression of environmentally relevant genes [55].

A. aurescens TC1 is likely to sequester significant levels of manganese, which might be important for its resistance to oxidative stress [24]. A. aurescens TC1 contains a homolog (AAur_3914) to MndD from A. globiformis and other arthrobacteria, a manganese-dependent dioxygenase [56] showing resistance to H_2_O_2_ inactivation [56–58]. Other enzymes, which contain alternative metals, also contain manganese (II), based on genome annotation evidence for A. aurescens TC1. *Arthrobacter* species were found to be the most numerically prevalent bacteria isolated from beneath leaking radionuclide storage tanks [14], and preliminary studies indicate that A. aurescens TC1 is significantly resistant to ionizing radiation in the laboratory (M. Daly, unpublished data).

#### Genes involved in trehalose, glycogen, osmoticums, and other protective polysaccharides.

Based on the genome sequence, *A. aurescens* TC1 produces glycogen and trehalose, both of which have been found in A. globiformis [34]. Moreover, we have used in vivo nuclear magnetic resonance and observed the formation of trehalose in osmotically stressed cells of A. aurescens TC1 (data not shown). Trehalose has been shown to accumulate under extreme water stress conditions in bacteria and affords cell desiccation tolerance [59]. The genes for both the biosynthesis and catabolism of trehalose (AAur_0306, AAur_0909, AAur_2895, AAur_2896, AAur_4069, AAur_0930, AAur_0931, and AAur_3722) and glycogen (AAur_2137) are present in A. aurescens TC1, which is expected for an osmoprotectant that would be formed transiently and degraded when not needed. The glycogen synthetic branching enzyme (AAur_2897, AAur_0691, AAur_0904) is most commonly found in fungi and soil bacteria.

Bacteria exposed to osmotic stress also maintain equilibrium by the accumulation of organic osmolytes, such as glycine betaine (N,N,N-trimethylglycine) [60]. A. aurescens TC1 contains both *betA* (choline dehydrogenase) and *betB* (betaine aldehyde dehydrogenase) genes (AAur_0512 and AAur_0513, respectively), located most likely as an operon. An helix–turn–helix transcriptional regulator, *betI* (AAur_0516), is also present near this operon. In addition, a second copy of *betA* (AAur_3606), and two clusters of ABC-type glycine betaine/choline transport genes (similar to *proX*, *proZ,* and *proW*) were also identified in the A. aurescens genome (AAur_2814–AAur_2817 and AAur_0644–AAur_0647). A potential *proP*-like proline–betaine–ectoine transporter (AAur_0280) was also present, suggesting that exogenous choline can serve as substrate for glycine betaine synthesis. A. pascens and A. globiformis have been reported to use a soluble choline oxidase to catalyze both steps of glycine betaine sysnthesis [61]. Osmotic stress in this bacterium may also be modulated by the control of water movement into the cell via an aquaporin Z (*aqpZ*) (AAur_2559) homolog, having about 61% amino acid similarity to *aqpZ* from *Sinorhizobium meliloti.*

#### Cupins in *Arthrobacter* and relation to stress, manganese accumulation, and morphogenesis.

Cupins, a superfamily of β-barrel structural domains, are thought to be involved in stress responses, cell morphogenesis and development, cell wall structure, and desiccation tolerance [62]. Cupin superfamily enzymes include several dioxygenases and plant-associated germins [62] that bind a single manganese ion, similar to manganese superoxide dismutase (MnSOD) [63]. The A. aurescens TC1 contains 14 cupin domain–containing proteins, 11 of which are located on the chromosome, and one on each of the two plasmids (Table S6). While several microbial genomes have been reported to contain from two to seven cupin genes, the stress-responsive *A. aurescens, B. subtilis,* and *Synechocystis* genomes contain 15–20 copies. The majority (81%), of A. aurescens cupin-containing genes contain a single cupin domain, while gentisate dioxygenase (AAur_0331) and AAur_3409 have a two-domain cupin composition (Table S6). Four of the mono domain cupin-containing proteins in A. aurescens (AAur_3964, AAur_1055, AAur_0978 and AAur_1082) have a C-terminal cupin and are most likely helix–turn–helix regulatory proteins. A. aurescens also contains a single cupin domain, pirin-like gene (AAur_2822) (Table S6), a homolog of which in Synechocystis sp. PCC 6803 is induced under salt and other stress conditions [64].

#### Other stress-responsive genes.

A. aurescens TC1 appears to be well poised to respond to a variety of environmental stresses. The TC1 chromosome was found to encode universal stress-related proteins (USPs), heat- and cold-shock proteins, general stress proteins, starvation-inducible proteins, and proteins involved in osmotic sensing and response (Table S6). The USPs represent a superfamily of proteins (accession number listed in Supporting Information) that are induced in cells in response to carbon, nitrogen, and phosphate starvation, exposure to heat, entering stationary phase, and UV exposure [65]. Genome analyses indicate that organisms exposed to stress conditions have a greater number of USPs than intracellular parasites. Halobacterium sp. strain NRC-1 has eight *usp* genes, while *Ricketsia*, *Mycoplasma,* and Chlamydia sp. strains have only one. The A. aurescens genome contains eight ORFs (AAur_0044, AAur_0235, AAur_0410, AAur_0506, AAur_0701, AAur_2837, AAur_3886, and AAur_4058) encoding members of the USP superfamily (Table S6). In addition, this bacterium contains several ORFs encoding heat- and cold-shock proteins, a gene region containing a dnaJ–dnaK–grpE operon (AAur_1876–AAur_1878), a putative HspR homolog (AAur_1879), and the chaperonins ClpB (AAur_1880) and groEL/ES (AAur_1001, AAur_2874, and AAur_2875). Interestingly, the genome of Arthrobacter sp. FB24 indicates the presence of approximately 15 potential USP superfamily members (Table 2). Since Arthrobacter sp. strains are subjected to daily fluctuation in temperature, osmotic potential, oxygen concentration, and other stresses, these USPs and other stress-related proteins may be involved in the survival of this bacterium under soil conditions.

#### Starvation-responsive genes.

The survival of A. aurescens TC1, and other autochthonous soil bacteria, under conditions of nutrient and other stresses most likely requires the presence of genes regulated, in part, by *rpoS* or σ^B^, alternative σ factors of RNA polymerase [66–69]. Expression of *rpoS* is repressed by RpsA and is regulated by homoserine lactones (HSLs) or a derivative [70]. A. aurescens has an *rpsA* homolog, AAur_0529 (encoding for a manganese-containing mandalate racemase family protein), which is most likely involved in starvation or stationary phase responses, and appears to synthesize HSLs from homoserine via the threonine biosynthetic pathway beginning with L-aspartate, in which AAur_0661, AAur_2995, and AAur_2612 encode aspartate kinase, aspartate semialdehyde dehydrogenase, and homoserine dehydrogenase, respectively. Quorum sensing has also been shown to regulate the expression of catalase and superoxide dismutase genes [71], further linking A. aurescens HSL synthesis genes to oxygen and starvation stress. A. aurescens also possesses several other genes involved in starvation survival responses, including the carbon-starvation protein CstA (AAur_0848), which has been shown to positively regulate the cAMP-CRP-dependent carbon starvation response [72].

#### Biodegradative capabilities.

Of the 326 microorganisms (encompassing 83 bacterial genera) in the University of Minnesota Biocatalysis/Biodegradation Database (http://umbbd.ahc.umn.edu/cgi-bin/micro.cgi), Arthrobacter sp. strains are the third most abundant in catalogued biotransformation reactions, only less than *Pseudomonas* and Rhodococcus sp. strains. *Arthrobacter* strains are metabolically diverse and are capable of catabolizing a variety of chemical compounds. The present genome project revealed that A. aurescens TC1 is particularly well-endowed genetically to metabolize amines [45]. It contains on the order of a dozen amine oxidases (Table S6). Indeed, we have shown that the extraordinary amine metabolism of A. aurescens TC1, coupled with plasmid enzymes that metabolize secondary amines to primary amines, can together provide for the metabolism of more than 500 s-triazine ring compounds [45].

While members of the genus *Arthrobacter* have been noted for their ability to catabolize various environmentally relevant compounds, including pollutants such as glyphosate, methyl *tert*-butyl ether, 2,4-dichlorophenoxyacetate (2,4-D), nictotine, 4-nitrophenol, dimethylsilanediol, endoxohexahydrophthalate (endothal), fluorene, phthalate, and nitroglycerine [25–31,73–75], A. aurescens TC1 does not contain genes or pathways for the catabolism of any of these compounds. In contrast, A. aurescens TC1 appears very specialized with respect to its ability to use a large variety of nitrogenous compounds as a source of nitrogen for growth. About 1.85% of the A. aurescens genome is dedicated to the degradation of proteins, peptides, and glycopeptides, further extending its ability to catabolize a large array of nitrogenous compounds for growth.

#### Catabolism of naturally occurring polymers.

A significant metabolic niche of A. aurescens TC1 and Arthrobacter sp. FB24 is in the decomposition of carbohydrate polymers. For example, these strains may express on the order of two dozen amylase family proteins, which are putatively excreted. They also make enzymes for oligomeric carbohydrate degradation, as well as for the hydrolysis of pectin, glucoside, and xylan. A. aurescens TC1 makes multiple inulinases. By contrast, there are no annotated inulinases produced by P. putida KT2440. In this context, Arthrobacter sp. likely occupy an important niche in nature biodegrading carbohydrate polymers and humic substances.

### Conclusions

A. aurescens strain TC1 is an autochthonous soil bacterium that has the ability to survive for long periods of time in a variety of environmental conditions. Its ability to survive is intimately tied to its genomic versatility, especially with respect to nitrogen metabolism and the ability to grow on polymeric substrates that are often not used by many soil microbes. This most likely gives this bacterium a competitive advantage in oligotrophic soil environments. In addition, this bacterium's impressive array of genes and mechanisms allowing for survival in stressful soil conditions, coupled with its ability to produce a temperature-tolerant “cyst”-like resting cell, makes *Arthrobacter* a truly ubiquitous soil microorganism that is well poised to survive and prosper in a great variety of environmental conditions.

## Materials and Methods

### A. aurescens strain TC1 isolation and propagation.

A. aurescens strain TC1 was previously isolated, by direct plating and without enrichment, from a South Dakota spill-site soil containing high concentrations (up to 29,000 μg/g) of atrazine as described [31]. The bacterium was routinely grown at 30 °C in modified R minimal medium [76] containing 500 μg/g atrazine as the sole nitrogen and carbon source. Total genomic DNA was isolated and twice purified by CsCl density gradient centrifugation as previously described [77].

### Sequencing, assembly, and gap closure.

Cloning, sequencing, and assembly were performed as described previously for genomes sequenced at TIGR [78]. In brief, one small-insert plasmid library (1–2 kb) and one medium-insert plasmid library (10–12 kb) were generated by random nebulization and cloning of genomic DNA. In the random sequencing phase, 8.7-fold coverage was achieved from the two libraries. The sequences were assembled using TIGR Assembler (www.tigr.org/software/sequencing.shtml) or Celera Assembler (http://sourceforge.net/projects/wgs-assembler), and the scaffolds constructed using TIGR BAMBUS (www.tigr.org/software/sequencing.shtml). All sequence and physical gaps were closed by editing the ends of sequence traces, primer walking or transposon-primed sequencing on plasmid clones, and combinatorial PCR followed by sequencing of the PCR product. A bacterial artificial chromosome (BAC) library (∼180 kb inserts) made in pCUGIBAC1 [46] was used in the genome closure process.

The *Arthrobacter* genome contained a significant number of areas difficult to sequence because of DNA secondary structures (“hard-stops”). Clones from the large insert libraries spanning the area of interest were initially amplified using the TempliPhi kit (Amersham Biosciences, http://www5.amershambiosciences.com) to generate a large amount of plasmid DNA. These clones were then sequenced using reaction mixes containing different cocktails of dGTP (ABI), BigDye Terminator V3.1 (Applied Biosystems, https://products.appliedbiosystems.com) and betaine (Sigma-Aldrich http://www.sigmaaldrich.com). In addition, some of the clones were amplified by PCR with the nucleotide analog 7-deaza-dGTP (Roche Diagnostics, http://www.roche-diagnostics.com) and sequenced. Implementation of these techniques helped in destabilizing the hard-stop regions and obtaining a sequence through it. An example of such hard-stops, the longest in the TC1 genome, is shown in Figure S3. This hairpin loop is located approximately 430 bp upstream of the predicted origin of replication and is 66 bp long.

A remarkable feature of the pTC1 plasmid was the presence of six identical tandem repeats of about 16 kb. Because of the large size of each repeat unit, the size of the whole repeated region (∼96 kb), the fact that each repeat unit was perfectly identical to its neighbors, and the absence of unique sequences between each unit, this repeated region of pTC1 could not be resolved at the time of publication of this manuscript and is therefore still collapsed in the pTC1 sequence assembly deposited in GenBank. However, several lines of evidence allowed us to determine the exact number of repeat copies, as well as the approximate coordinates of the repeat region in the pTC1 sequence. First, the average coverage of the area containing the collapsed repeat was 58.9 + 7.6, whereas the regions before and after the collapsed repeat region had an average coverage of 18.8 + 4.2 and 13.3 + 3.1, respectively. Second, a BAC clone containing an insert spanning the entire ∼96-kb repeat region was shotgun sequenced (a total of 1,566 reads were sequenced) and assembled into two contigs that matched and confirmed the plasmid pTC1 assembly. The coverage in the unique area of the BAC assembly was between 8- to 12-fold, as expected, whereas the coverage in the repeat area was about 70-fold. The size of the assembled BAC was about 45 kb, and the whole BAC size was estimated by PFGE to be 77.2 + 5 kb, further confirming the presence of six identical repeat units of approximately 16 kb each. The corrected size of pTC1 inclusive of the six identical repeat units of about 16 kb is approximately 408,237 bp.

### ORF prediction and gene family identification.

An initial set of ORFs likely to encode proteins was identified by GLIMMER (www.tigr.org/software/genefinding.shtml), and those shorter than 30 codons (90 nucleotides) were eliminated. ORFs that overlapped were inspected visually and, in some cases, removed. ORFs were searched against a nonredundant protein database as described previously for all TIGR genomes. Frameshifts and point mutations were detected and corrected where appropriate as described previously [78]. Remaining frameshifts and point mutations are considered authentic, and corresponding regions were annotated as “authentic frameshift” or “authentic point mutation,” respectively. Two sets of hidden Markov models (HMMs) were used to determine ORF membership in families and superfamilies. These included 8,163 HMMs from PFAM version 2.0 (www.sanger.ac.uk/Software/Pfam/index.shtml) and 2,998 HMMs from the TIGR orthologue resource (www.tigr.org/TIGRFAMs/index.shtml). TOPPRED was used to identify membrane-spanning domains in proteins.

### Comparative genomics.

All genes and predicted proteins from the A. aurescens TC1 genome, as well as from all other published completed genomes (see http://cmr.tigr.org/tigr-scripts/CMR/CmrHomePage.cgi), were compared using BLAST. For the identification of recent gene duplications, all genes from the A. aurescens TC1 genome were searched against a nonredundant database of completed microbial genomes, to which the A. aurescens TC1 genome was added. A gene was considered to be recently duplicated if the most similar gene (as measured by *p*-value) was another gene within the TC1 genome (relative to genes from other genomes).

### GenBank submission.

The nucleotide sequence of the whole genome of A. aurescens strain TC1 was submitted to GenBank (accession numbers listed in Supporting Information). The complete genome sequence is also available through the TIGR Comprehensive Microbial Resource web site (http://cmr.tigr.org/tigr-scripts/CMR/CmrHomePage.cgi).

## Supporting Information

Figure S1 Whole Genome Alignments between *A. aurescens TC1* and Arthrobacter sp. FB24 (A) Nucmer alignment comparing the nucleotide sequences of the TC1 genome (x-axis) to the FB24 genome (y-axis). The Nucmer algorithm was used to calculate and plot the nucleotide percentage of similarity (scale on the right side) of maximally matching sequences of at least 20 nucleotides between the two genomes.
(B) Promer alignment comparing the amino-acid sequences of the TC1 genome (x-axis) to the FB24 genome (y-axis). The Promer algorithm was used to calculate and plot the amino-acid percentage similarity (scale on the right side) of maximally matching subsequences of at least five amino acids between the two genomes.
(777 KB PDF)

Figure S2 Functional Role Category Distribution of A. aurescens TC1 Genes with BLASTP Best Matches to *L. xyli, S. coelicolor, S. avermitilis, Nocardia farcinica*, and Thermobifida fusca
(56 KB PDF)

Figure S3 Secondary Structure of the Longest Hairpin Loop in A. aurescens TC1 Chromosomal DNA. The following ionic conditions were used for the computation: [Na^+^] = 1.0 M, [Mg^++^] = 0.0 M. The calculated ΔG0 was −34.3 kcal/mole at 37 °C, and the calculated Tm was 91.1 °C. The folding of the DNA sequence (coordinates: 4597663–4597743) was computed using the mfold (version 3.1) web server at http://www.bioinfo.rpi.edu/applications/mfold/dna/form1.cgi [79].
(301 KB PDF)

Table S1 List of the A. aurescens TC1 Genes with BLAST Best Matches to Other TC1 Genes (787 KB DOC)

Table S2 List of the A. aurescens Chromosomally Encoded Genes with Atypical Nucleotide Composition Regions of atypical composition were analyzed by χ^2^ analysis of the nucleotide composition along the chromosome. In brief, the distribution of all 64 trinucleotides (3-mers) was computed for the complete chromosome, followed by the 3-mer distribution in 2,000-bp windows across the chromosome (the window was shifted by 1,000 bp during the computation). For each window, the χ^2^ statistic was computed on the difference between its 3-mer content and that of the whole chromosome. A large value of this statistic means that the composition within the window is different from the rest of the genome, based on the assumption that the DNA composition is relatively uniform throughout the genome.
(329 KB DOC)

Table S3 List of the A. aurescens pTC1 and pTC2 Plasmid Genes Matching Chromosomal Genes by BLASTP Searches (415 KB DOC)

Table S4 List of the Genes Shared among A. aurescens pTC1, Pseudomonas sp. pADP1, and A. nicotinivorans pAO1 Plasmids (124 KB DOC)

Table S5 List of the A. aurescens TC1 Unique Genes, Compared with the Genome of Arthrobacter sp. FB24 (964 KB DOC)

Table S6 Genes in the A. aurescens TC1 Genome Involved in Stress Survival (242 KB DOC)

### Accession Numbers

The GenBank (http://www.ncbi.nlm.nih.gov) accession numbers for the genomes discussed in this paper are Arthrobacter sp. FB24 (NC_008541, NC_008537, NC_008538, and NC_008539), A. aurescens (TC1 chromosome [CP000474], pTC1 plasmid [CP000475], and pTC2 plasmid [CP000476]), S. coelicolor A3(2) chromosome (NC_003888), and L. xyli subsp. xyli str. CTCB07 (NC_006087).

The PFAM (http://www.sanger.ac.uk/Software/Pfam/index.shtml) accession number for the USP family is PF00582.

A. aurescens strain TC1 has been deposited in the American Type Culture Collection (http://www.atcc.org) under the accession number BAA-1386.

## Abbreviations

BAC: bacterial artificial chromosome
HMM: hidden Markov model
HSL: homoserine lactone
IS: insertion sequence
ORF: open reading frame
TIGR: The Institute for Genomic Research
USP: universal stress-related proteins

## References

[pgen-0020214-b001] Koch C, Schumann P, Stackebrandt E (1995). Reclassification of Micrococcus agilis (Ali-Cohen 1889) to the genus *Arthrobacter* as Arthrobacter agilis comb. nov. and emendation of the genus *Arthrobacter*. Int J Syst Bacteriol.

[pgen-0020214-b002] Hagedorn C, Holt JG (1975). A nutritional and taxonomic survey of *Arthrobacter* soil isolates. Can J Microbiol.

[pgen-0020214-b003] Lowe WE, Gray TRG (1972). Ecological studies on coccoid bacteria in a pine forest soil. I. Classification. Soil Biol Biochem.

[pgen-0020214-b004] Paul EA, Clark FE (1989). Soil microbiology and biochemistry.

[pgen-0020214-b005] Skyring GW, Quadling C (1969). Soil bacteria: Comparisons of rhizosphere and nonrhizosphere populations. Can J Microbiol.

[pgen-0020214-b006] Soumare S, Blondeau R (1972). [Microbiological properties of soils in Northern France: Arthrobacter extent]. Ann Inst Pasteur (Paris).

[pgen-0020214-b007] Keddie RM, Jones D, Starr MP, Stolp H, Truper HG, Balows A, Schlegel HG (1981). Saprophytic, aerobic coryneform bacteria. The Prokaryotes: A handbook on habitats, isolation and identification of bacteria.

[pgen-0020214-b008] Jones D, Keddie RM, Balows A, Truper HG, Dworkin M, Harder W, Schleifer KH (1992). The genus Arthrobacter. The Prokaryotes.

[pgen-0020214-b009] Fong NJ, Burgess ML, Barrow KD, Glenn DR (2001). Carotenoid accumulation in the psychrotrophic bacterium Arthrobacter agilis in response to thermal and salt stress. Appl Microbiol Biotechnol.

[pgen-0020214-b010] Hirsch P (1986). Microbial life at extremely low nutrient levels. Adv Space Res.

[pgen-0020214-b011] Ryan KR, Shapiro L (2003). Temporal and spatial regulation in prokaryotic cell cycle progression and development. Annu Rev Biochem.

[pgen-0020214-b012] Smyk B (1970). Fixation of atmospheric nitrogen by the strains of *Arthrobacter*. Zentralbl Bakteriol Parasitenkd Infektionskr Hyg.

[pgen-0020214-b013] Smyk B, Ettlinger L (1963). Research on various species of nitrogen-fixing *Arthrobacter* isolated from Karstic alpine rocks. Ann Inst Pasteur (Paris).

[pgen-0020214-b014] Fredrickson JK, Zachara JM, Balkwill DL, Kennedy D, Li SM (2004). Geomicrobiology of high-level nuclear waste-contaminated vadose sediments at the Hanford site, Washington state. Appl Environ Microbiol.

[pgen-0020214-b015] Boylen CW (1973). Survival of Arthrobacter crystallopoietes during prolonged periods of extreme desiccation. J Bacteriol.

[pgen-0020214-b016] Boylen CW, Ensign JC (1970). Intracellular substrates for endogenous metabolism during long-term starvation of rod and spherical cells of Arthrobacter crystallopoietes. J Bacteriol.

[pgen-0020214-b017] Labeda DP, Liu KC, Casida LE (1976). Colonization of soil by *Arthrobacter* and *Pseudomonas* under varying conditions of water and nutrient availability as studied by plate counts and transmission electron microscopy. Appl Environ Microbiol.

[pgen-0020214-b018] Robinson JB, Salonius PO, Chase FE (1965). A note on the differential response of Arthrobacter spp. and Pseudomonas spp. to drying in soil. Can J Microbiol.

[pgen-0020214-b019] Zevenhuizen LP (1966). Formation and function of the glycogen-like polysaccharide of *Arthrobacter*. Antonie Van Leeuwenhoek.

[pgen-0020214-b020] Cameron RE, Conrow HP (1971). Survival of antarctic desert soil bacteria exposed to various temperatures and three years of continuous medium high vacuum.

[pgen-0020214-b021] Ensign JC (1970). Long-term starvation survival of rod and spherical cells of Arthrobacter crystallopoietes. J Bacteriol.

[pgen-0020214-b022] Germida JJ, Casida LE (1980). Myceloid growth of Arthrobacter globiformis and other *Arthrobacter* species. J Bacteriol.

[pgen-0020214-b023] Meganathan R, Ensign JC (1976). Stability of enzymes in starving Arthrobacter crystallopoietes. J Gen Microbiol.

[pgen-0020214-b024] Daly MJ, Gaidamakova EK, Matrosova VY, Vasilenko A, Zhai M (2004). Accumulation of Mn(II) in Deinococcus radiodurans facilitates gamma-radiation resistance. Science.

[pgen-0020214-b025] Eaton RW (2001). Plasmid-encoded phthalate catabolic pathway in Arthrobacter keyseri 12B. J Bacteriol.

[pgen-0020214-b026] Gil M, Haidour A, Ramos JL (2000). Degradation of *o*-methoxybenzoate by a two-member consortium made up of a gram-positive *Arthrobacter* strain and a gram-negative *Pantotea* strain. Biodegradation.

[pgen-0020214-b027] Jensen HL (1964). Studies on soil bacteria (Arthrobacter globiformis) capable of decomposing the herbicide Endothal. Acta Agric Scand.

[pgen-0020214-b028] Liu CY, Speitel GE, Georgiou G (2001). Kinetics of methyl *t*-butyl ether cometabolism at low concentrations by pure cultures of butane-degrading bacteria. Appl Environ Microbiol.

[pgen-0020214-b029] Marshall SJ, White GF (2001). Complete denitration of nitroglycerin by bacteria isolated from a washwater soakaway. Appl Environ Microbiol.

[pgen-0020214-b030] Strong LC, McTavish H, Sadowsky MJ, Wackett LP (2000). Field-scale remediation of atrazine-contaminated soil using recombinant Escherichia coli expressing atrazine chlorohydrolase. Environ Microbiol.

[pgen-0020214-b031] Strong LC, Rosendahl C, Johnson G, Sadowsky MJ, Wackett LP (2002). Arthrobacter aurescens TC1 metabolizes diverse *s*-triazine ring compounds. Appl Environ Microbiol.

[pgen-0020214-b032] Fan F, Ghanem M, Gadda G (2004). Cloning, sequence analysis, and purification of choline oxidase from Arthrobacter globiformis: A bacterial enzyme involved in osmotic stress tolerance. Arch Biochem Biophys.

[pgen-0020214-b033] Sakamoto A, Murata N (2000). Genetic engineering of glycinebetaine synthesis in plants: Current status and implications for enhancement of stress tolerance. J Exp Bot.

[pgen-0020214-b034] Zevenhuizen LP (1992). Levels of trehalose and glycogen in Arthrobacter globiformis under conditions of nutrient starvation and osmotic stress. Antonie Van Leeuwenhoek.

[pgen-0020214-b035] Igloi GL, Brandsch R (2003). Sequence of the 165-kilobase catabolic plasmid pAO1 from Arthrobacter nicotinovorans and identification of a pAO1-dependent nicotine uptake system. J Bacteriol.

[pgen-0020214-b036] Makino K, Amemura M, Kim SK, Nakata A, Shinagawa H (1993). Role of the sigma 70 subunit of RNA polymerase in transcriptional activation by activator protein PhoB in Escherichia coli. Genes Dev.

[pgen-0020214-b037] Gevers D, Vandepoele K, Simillon C, Van de Peer Y (2004). Gene duplication and biased functional retention of paralogs in bacterial genomes. Trends Microbiol.

[pgen-0020214-b038] Nelson KE, Weinel C, Paulsen IT, Dodson RJ, Hilbert H (2002). Complete genome sequence and comparative analysis of the metabolically versatile Pseudomonas putida KT2440. Environ Microbiol.

[pgen-0020214-b039] Weinel C, Nelson KE, Tummler B (2002). Global features of the Pseudomonas putida KT2440 genome sequence. Environ Microbiol.

[pgen-0020214-b040] Reva ON, Tummler B (2005). Differentiation of regions with atypical oligonucleotide composition in bacterial genomes. BMC Bioinformatics.

[pgen-0020214-b041] Kranz RG, Gabbert KK, Locke TA, Madigan MT (1997). Polyhydroxyalkanoate production in Rhodobacter capsulatus: Genes, mutants, expression, and physiology. Appl Environ Microbiol.

[pgen-0020214-b042] Brazilian National Genome Project (BNGP) Consortium (2003). The complete genome sequence of Chromobacterium violaceum reveals remarkable and exploitable bacterial adaptability. Proc Natl Acad Sci U S A.

[pgen-0020214-b043] Lyi SM, Jafri S, Winans SC (1999). Mannopinic acid and agropinic acid catabolism region of the octopine-type Ti plasmid pTi15955. Mol Microbiol.

[pgen-0020214-b044] Kenna DT, Yesilkaya H, Forbes KJ, Barcus VA, Vandamme P (2006). Distribution and genomic location of active insertion sequences in the Burkholderia cepacia complex. J Med Microbiol.

[pgen-0020214-b045] Shapir N, Mongodin EF, Sadowsky MJ, Daugherty S, Nelson KE (2006). Evolution of catabolic pathways: Genomic insights into microbial *s*-triazine metabolism. J Bacteriol.

[pgen-0020214-b046] Sajjaphan K, Shapir N, Wackett LP, Palmer M, Blackmon B (2004). Arthrobacter aurescens TC1 atrazine catabolism genes *trzN*, *atzB*, and *atzC* are linked on a 160-kilobase region and are functional in Escherichia coli. Appl Environ Microbiol.

[pgen-0020214-b047] de AzevWasch SI, van der Ploeg JR, Maire T, Lebreton A, Kiener A (2002). Transformation of isopropylamine to L-alaninol by Pseudomonas sp. strain KIE171 involves N-glutamylated intermediates. Appl Environ Microbiol.

[pgen-0020214-b048] Martinez B, Tomkins J, Wackett LP, Wing R, Sadowsky MJ (2001). Complete nucleotide sequence and organization of the atrazine catabolic plasmid pADP-1 from Pseudomonas sp. strain ADP. J Bacteriol.

[pgen-0020214-b049] Barbour WM, Mathis JN, Elkan GH (1985). Evidence for plasmid- and chromosome-borne multiple *nif* genes in Rhizobium fredii. Appl Environ Microbiol.

[pgen-0020214-b050] Guerrero G, Peralta H, Aguilar A, Diaz R, Villalobos MA (2005). Evolutionary, structural and functional relationships revealed by comparative analysis of syntenic genes in *Rhizobiales*. BMC Evol Biol.

[pgen-0020214-b051] Alice AF, Lopez CS, Crosa JH (2005). Plasmid- and chromosome-encoded redundant and specific functions are involved in biosynthesis of the siderophore anguibactin in Vibrio anguillarum 775: A case of chance and necessity?. J Bacteriol.

[pgen-0020214-b052] Erauso G, Stedman KM, van de Werken HJ, Zillig W, van der Oost J (2006). Two novel conjugative plasmids from a single strain of *Sulfolobus*. Microbiology.

[pgen-0020214-b053] Ulrich LE, Koonin EV, Zhulin IB (2005). One-component systems dominate signal transduction in prokaryotes. Trends Microbiol.

[pgen-0020214-b054] Hungria M, Nicolas MF, Guimaraes CT, Jardim SN, Gomes EA (2004). Tolerance to stress and environmental adaptability of Chromobacterium violaceum. Genet Mol Res.

[pgen-0020214-b055] Nachin L, El Hassouni M, Loiseau L, Expert D, Barras F (2001). SoxR-dependent response to oxidative stress and virulence of Erwinia chrysanthemi: The key role of SufC, an orphan ABC ATPase. Mol Microbiol.

[pgen-0020214-b056] Vetting MW, Wackett LP, Que L, Lipscomb JD, Ohlendorf DH (2004). Crystallographic comparison of manganese- and iron-dependent homoprotocatechuate 2,3-dioxygenases. J Bacteriol.

[pgen-0020214-b057] Emerson JP, Wagner ML, Reynolds MF, Que L, Sadowsky MJ (2005). The role of histidine 200 in MndD, the Mn(II)-dependent 3,4-dihydroxyphenylacetate 2,3-dioxygenase from Arthrobacter globiformis CM-2, a site-directed mutagenesis study. J Biol Inorg Chem.

[pgen-0020214-b058] Olson PE, Qi B, Que L, Wackett LP (1992). Immunological demonstration of a unique 3,4-dihydroxyphenylacetate 2,3-dioxygenase in soil *Arthrobacter* strains. Appl Environ Microbiol.

[pgen-0020214-b059] Potts M (1994). Desiccation tolerance of prokaryotes. Microbiol Rev.

[pgen-0020214-b060] Boscari A, Mandon K, Poggi MC, Le Rudulier D (2004). Functional expression of Sinorhizobium meliloti BetS, a high-affinity betaine transporter, in Bradyrhizobium japonicum USDA110. Appl Environ Microbiol.

[pgen-0020214-b061] Osteras M, Boncompagni E, Vincent N, Poggi MC, Le Rudulier D (1998). Presence of a gene encoding choline sulfatase in *Sinorhizobium meliloti bet* operon: Choline-*O*-sulfate is metabolized into glycine betaine. Proc Natl Acad Sci U S A.

[pgen-0020214-b062] Dunwell JM, Khuri S, Gane PJ (2000). Microbial relatives of the seed storage proteins of higher plants: Conservation of structure and diversification of function during evolution of the cupin superfamily. Microbiol Mol Biol Rev.

[pgen-0020214-b063] Woo EJ, Dunwell JM, Goodenough PW, Pickersgill RW (1998). Barley oxalate oxidase is a hexameric protein related to seed storage proteins: Evidence from X-ray crystallography. FEBS Lett.

[pgen-0020214-b064] Hihara Y, Muramatsu M, Nakamura K, Sonoike K (2004). A cyanobacterial gene encoding an ortholog of Pirin is induced under stress conditions. FEBS Lett.

[pgen-0020214-b065] Kvint K, Nachin L, Diez A, Nystrom T (2003). The bacterial universal stress protein: Function and regulation. Curr Opin Microbiol.

[pgen-0020214-b066] Mulvey MR, Loewen PC (1989). Nucleotide sequence of *katF* of Escherichia coli suggests KatF protein is a novel sigma transcription factor. Nucleic Acids Res.

[pgen-0020214-b067] Hecker M, Volker U (1998). Non-specific, general and multiple stress resistance of growth-restricted Bacillus subtilis cells by the expression of the sigmaB regulon. Mol Microbiol.

[pgen-0020214-b068] Pragai Z, Harwood CR (2002). Regulatory interactions between the Pho and sigma(B)-dependent general stress regulons of Bacillus subtilis. Microbiology.

[pgen-0020214-b069] Venturi V (2003). Control of *rpoS* transcription in Escherichia coli and *Pseudomonas*: Why so different?. Mol Microbiol.

[pgen-0020214-b070] Huisman GW, Kolter R (1994). Sensing starvation: A homoserine lactone-dependent signaling pathway in Escherichia coli. Science.

[pgen-0020214-b071] Hassett DJ, Ma JF, Elkins JG, McDermott TR, Ochsner UA (1999). Quorum sensing in Pseudomonas aeruginosa controls expression of catalase and superoxide dismutase genes and mediates biofilm susceptibility to hydrogen peroxide. Mol Microbiol.

[pgen-0020214-b072] Schultz JE, Matin A (1991). Molecular and functional characterization of a carbon starvation gene of Escherichia coli. J Mol Biol.

[pgen-0020214-b073] Cacciari I, Giovannozzi-Sermanni G, Grappelli A, Lippi D (1971). Nitrogen fixation by Arthrobacter sp. I. Taxonomic study and evidence of nitrogenase activity of two new strains. Ann Microbiol Enzimol.

[pgen-0020214-b074] Loos MA, Roberts RN, Alexander M (1967). Phenols as intermediates in the decomposition of phenoxyacetates by an *Arthrobacter* species. Can J Microbiol.

[pgen-0020214-b075] Smalla K, Wieland G, Buchner A, Zock A, Parzy J (2001). Bulk and rhizosphere soil bacterial communities studied by denaturing gradient gel electrophoresis: Plant-dependent enrichment and seasonal shifts revealed. Appl Environ Microbiol.

[pgen-0020214-b076] Selifonova O, Burlage R, Barkay T (1993). Bioluminescent sensors for detection of bioavailable Hg(II) in the environment. Appl Environ Microbiol.

[pgen-0020214-b077] Sadowsky MJ, Tully RE, Cregan PB, Keyser HH (1987). Genetic diversity in Bradyrhizobium japonicum serogroup 123 and its relation to genotype-specific nodulation of soybean. Appl Environ Microbiol.

[pgen-0020214-b078] Fouts DE, Mongodin EF, Mandrell RE, Miller WG, Rasko DA (2005). Major structural differences and novel potential virulence mechanisms from the genomes of multiple *Campylobacter* species. PLoS Biol.

[pgen-0020214-b079] Zuker M (2003). Mfold web server for nucleic acid folding and hybridization prediction. Nucleic Acids Res.

