# Supplementary material for: Secrets of Soil Survival Revealed by the Genome Sequence of Arthrobacter aurescens TC1
Source: PLoS Genet. 2006 Dec 22;2(12):e214. doi: 10.1371/journal.pgen.0020214 (PMC1713258; doi:10.1371/journal.pgen.0020214)
Supplement: Supplementary file 4 [file pgen.0020214.st001.doc]

**Supplemental Table 1**. List of the *Arthrobacter aurescens* TC1 genes having their best BLAST match to other TC1 genes.

| Query (Q) | Match (M) | Length Q | Length M | %M /  Length Q | % Identity | % Similarity | E-value | Predicted Protein Product |
| --- | --- | --- | --- | --- | --- | --- | --- | --- |
| AAur_0045 | AAur_0973 | 1503 bp | 1539 | 96.61 | 68.84 | 68.84 | 3.40E-129 | amino acid permease putative |
| AAur_0073 | AAur_pTC10104 | 1122 bp | 1146 | 99.55 | 81.79 | 81.79 | 4.90E-163 | Conserved hypothetical protein |
| AAur_0074 | AAur_pTC10103 | 858 bp | 606 | 70.51 | 85.97 | 85.97 | 3.90E-96 | Hypothetical protein |
| AAur_0075 | AAur_pTC10099 | 447 bp | 450 | 98.88 | 90.77 | 90.77 | 9.10E-77 | Hypothetical protein |
| AAur_0076 | AAur_pTC10098 | 450 bp | 453 | 99.11 | 88.37 | 88.37 | 1.60E-73 | Hypothetical protein |
| AAur_0081 | AAur_3839 | 2082 bp | 1146 | 49.71 | 57.57 | 57.57 | 1.00E-30 | FG-GAP repeat domain protein |
| AAur_0083 | AAur_0713 | 1089 bp | 1023 | 84.39 | 59.50 | 59.50 | 2.70E-35 | oxidoreductase family NAD-binding Rossmann fold domain protein |
| AAur_0089 | AAur_3136 | 1467 bp | 2526 | 41.92 | 69.81 | 69.81 | 4.00E-52 | S-layer domain protein putative |
| AAur_0104 | AAur_0137 | 1029 bp | 978 | 37.80 | 62.37 | 62.37 | 1.80E-21 | conserved hypothetical protein |
| AAur_0108 | AAur_0299 | 741 bp | 774 | 90.15 | 62.95 | 62.95 | 5.30E-34 | ANTAR domain protein |
| AAur_0137 | AAur_0104 | 978 bp | 1029 | 39.37 | 62.37 | 62.37 | 1.70E-21 | conserved hypothetical protein |
| AAur_0149 | AAur_1645 | 123 bp | 129 | 65.04 | 97.53 | 97.53 | 1.20E-10 | hypothetical protein |
| AAur_0181 | AAur_3219 | 153 bp | 165 | 90.85 | 68.97 | 68.97 | 4.20E-06 | hypothetical protein |
| AAur_0208 | AAur_pTC20242 | 792 bp | 744 | 86.87 | 60.37 | 60.37 | 2.80E-30 | ANTAR domain protein |
| AAur_0210 | AAur_0108 | 738 bp | 741 | 90.24 | 58.27 | 58.27 | 1.90E-19 | ANTAR domain protein |
| AAur_0235 | AAur_3886 | 432 bp | 429 | 96.99 | 64.79 | 64.79 | 1.20E-25 | universal stress family domain protein putative |
| AAur_0236 | AAur_0237 | 1452 bp | 1983 | 87.26 | 63.85 | 63.85 | 2.40E-80 | pectinesterase/pectate lyase protein putative |
| AAur_0237 | AAur_0236 | 1983 bp | 1452 | 64.30 | 63.85 | 63.85 | 3.30E-80 | pectate lyase putative |
| AAur_0263 | AAur_0759 | 1605 bp | 1647 | 79.94 | 58.58 | 58.58 | 3.00E-47 | Sigma-70 region 2 domain protein putative |
| AAur_0276 | AAur_1391 | 402 bp | 405 | 91.79 | 65.07 | 65.07 | 2.20E-22 | glyoxalase family protein putative |
| AAur_0293 | AAur_3862 | 276 bp | 279 | 99.64 | 77.70 | 77.70 | 5.40E-30 | phosphocarrier protein HPr |
| AAur_0299 | AAur_0108 | 774 bp | 741 | 86.30 | 62.95 | 62.95 | 5.50E-34 | ANTAR domain protein |
| AAur_0313 | AAur_0975 | 1518 bp | 1539 | 50.79 | 77.89 | 77.89 | 6.50E-137 | HNH endonuclease domain protein putative |
| AAur_0316 | AAur_3995 | 573 bp | 540 | 92.84 | 68.99 | 68.99 | 1.20E-41 | transcriptional regulator MarR family putative |
| AAur_0325 | AAur_0327 | 1305 bp | 1389 | 67.97 | 65.10 | 65.10 | 9.30E-85 | integral membrane protein putative |
| AAur_0326 | AAur_2404 | 1608 bp | 1572 | 97.26 | 62.06 | 62.06 | 4.90E-85 | integral membrane protein putative |
| AAur_0327 | AAur_0325 | 1389 bp | 1305 | 63.79 | 65.10 | 65.10 | 9.90E-85 | integral membrane protein putative |
| AAur_0328 | AAur_1478 | 873 bp | 771 | 87.63 | 69.24 | 69.24 | 4.10E-67 | GDSL-like Lipase/Acylhydrolase domain protein |
| AAur_0330 | AAur_4033 | 837 bp | 855 | 94.27 | 64.79 | 64.79 | 1.90E-49 | 2-hydroxyhepta-2 4-diene-1 7-dioate isomerase putative |
| AAur_0339 | AAur_0350 | 807 bp | 810 | 96.41 | 66.96 | 66.96 | 1.80E-62 | transcriptional regulator DeoR family putative |
| AAur_0340 | AAur_3798 | 1053 bp | 939 | 59.16 | 57.28 | 57.28 | 1.30E-12 | D-threo-aldose 1-dehydrogenase putative |
| AAur_0350 | AAur_0339 | 810 bp | 807 | 96.42 | 66.96 | 66.96 | 1.80E-62 | transcriptional regulator DeoR family putative |
| AAur_0352 | AAur_1469 | 852 bp | 840 | 97.77 | 63.30 | 63.30 | 8.60E-45 | fructose/tagatose bisphosphate aldolase putative |
| AAur_0353 | AAur_3712 | 906 bp | 897 | 99.23 | 62.66 | 62.66 | 4.50E-48 | aldose-1-epimerase putative |
| AAur_0357 | AAur_0299 | 774 bp | 774 | 87.86 | 60.89 | 60.89 | 1.20E-31 | ANTAR domain protein |
| AAur_0387 | AAur_0559 | 1113 bp | 1128 | 96.95 | 64.46 | 64.46 | 3.40E-72 | fepD ferric enterobactin transport protein |
| AAur_0388 | AAur_0558 | 1089 bp | 1080 | 96.97 | 66.14 | 66.14 | 9.20E-81 | fepG ferric enterobactin transport system permease component |
| AAur_0389 | AAur_0557 | 843 bp | 822 | 90.75 | 75.26 | 75.26 | 1.50E-86 | fepC ferric enterobactin transport ATP-binding protein |
| AAur_0393 | AAur_0313 | 1446 bp | 1518 | 86.31 | 73.26 | 73.26 | 3.70E-134 | conserved hypothetical protein |
| AAur_0407 | AAur_pTC10196 | 633 bp | 630 | 86.73 | 59.00 | 59.00 | 4.90E-15 | Copper resistance protein CopC-family putative |
| AAur_0408 | AAur_4070 | 795 bp | 807 | 35.85 | 68.17 | 68.17 | 2.20E-19 | LysM domain protein putative |
| AAur_0414 | AAur_1960 | 924 bp | 945 | 95.89 | 65.75 | 65.75 | 1.90E-63 | fructokinase |
| AAur_0420 | AAur_pTC20008 | 603 bp | 630 | 99.83 | 81.05 | 81.05 | 2.70E-82 | site-specific recombinase resolvase family |
| AAur_0446 | AAur_3788 | 1032 bp | 939 | 83.82 | 90.87 | 90.87 | 5.30E-155 | hypothetical protein |
| AAur_0448 | AAur_3931 | 447 bp | 489 | 86.13 | 61.83 | 61.83 | 3.50E-17 | stress-like protein (gls24) putative |
| AAur_0479 | AAur_1022 | 1281 bp | 1359 | 69.79 | 61.76 | 61.76 | 4.10E-42 | molybdopterin biosynthesis protein (MoeA) putative |
| AAur_0486 | AAur_3824 | 681 bp | 672 | 96.92 | 63.76 | 63.76 | 1.20E-39 | transcription regulator gntR family |
| AAur_0487 | AAur_2306 | 915 bp | 1002 | 98.03 | 96.33 | 96.33 | 2.20E-183 | pyridoxine biosynthesis protein |
| AAur_0495 | AAur_0691 | 2154 bp | 2136 | 98.38 | 64.42 | 64.42 | 7.40E-148 | glycogen debranching enzyme family protein putative |
| AAur_0503 | AAur_3149 | 789 bp | 789 | 99.87 | 78.06 | 78.06 | 3.90E-100 | hypothetical protein |
| AAur_0507 | AAur_0605 | 1197 bp | 1539 | 65.41 | 76.97 | 76.97 | 8.20E-138 | HNH endonuclease domain protein putative |
| AAur_0510 | AAur_1502 | 1218 bp | 1764 | -97.21 | 61.98 | 61.98 | 5.10E-67 | M23 peptidase domain protein |
| AAur_0534 | AAur_3708 | 984 bp | 1023 | 99.80 | 68.17 | 68.17 | 9.90E-82 | transcriptional regulator lacI family |
| AAur_0542 | AAur_0543 | 174 bp | 174 | 98.85 | 75.57 | 75.57 | 6.20E-14 | hypothetical protein |
| AAur_0543 | AAur_0542 | 174 bp | 174 | 98.85 | 75.57 | 75.57 | 6.20E-14 | hypothetical protein |
| AAur_0557 | AAur_0389 | 822 bp | 843 | 93.43 | 75.26 | 75.26 | 1.40E-86 | fepC Ferric enterobactin transport ATP-binding protein |
| AAur_0558 | AAur_0388 | 1080 bp | 1089 | 97.87 | 66.14 | 66.14 | 9.10E-81 | fepG ferric enterobactin transport system permease component |
| AAur_0559 | AAur_0387 | 1128 bp | 1113 | 95.92 | 64.46 | 64.46 | 3.40E-72 | fepD ferric enterobactin transport system permease protein |
| AAur_0567 | AAur_3866 | 234 bp | 285 | 48.72 | 71.55 | 71.55 | 9.50E-06 | hypothetical protein |
| AAur_0578 | AAur_pTC20083 | 654 bp | 594 | 88.69 | 60.13 | 60.13 | 1.80E-19 | transcription regulator putative |
| AAur_0584 | AAur_3467 | 834 bp | 837 | 99.76 | 74.61 | 74.61 | 5.00E-93 | haloperoxidase |
| AAur_0599 | AAur_2845 | 681 bp | 681 | 99.71 | 99.56 | 99.56 | 3.10E-146 | hypothetical protein |
| AAur_0601 | AAur_1282 | 1137 bp | 1281 | 91.38 | 58.62 | 58.62 | 2.30E-41 | permease putative |
| AAur_0605 | AAur_0507 | 1539 bp | 1197 | 50.88 | 76.97 | 76.97 | 1.10E-137 | HNH endonuclease domain protein putative |
| AAur_0606 | AAur_3872 | 555 bp | 573 | 97.12 | 62.11 | 62.11 | 4.30E-28 | RNA polymerase sigma (70) factor putative |
| AAur_0626 | AAur_4053 | 1179 bp | 1203 | 96.27 | 66.55 | 66.55 | 2.50E-91 | GGDEF domain protein putative |
| AAur_0628 | AAur_3242 | 1419 bp | 1554 | 99.15 | 65.54 | 65.54 | 1.90E-106 | GGDEF domain/EAL domain protein putative |
| AAur_0653 | AAur_1331 | 1500 bp | 1482 | 98.00 | 72.18 | 72.18 | 1.10E-151 | succinate-semialdehyde dehydrogenase putative |
| AAur_0657 | AAur_pTC10151 | 300 bp | 303 | 99.67 | 73.03 | 73.03 | 1.70E-27 | Conserved hypothetical protein |
| AAur_0693 | AAur_1401 | 1503 bp | 1674 | 51.16 | 64.64 | 64.64 | 7.50E-47 | inulinase putative |
| AAur_0700 | AAur_0696 | 1352 bp | 1398 | 54.51 | 64.01 | 64.01 | 2.50E-50 | inulin fructotransferase (DFA-III-forming) putative |
| AAur_0715 | AAur_1893 | 1002 bp | 1014 | 99.60 | 97.70 | 97.70 | 2.60E-210 | conserved hypothetical protein |
| AAur_0756 | AAur_1502 | 1308 bp | 1764 | -41.90 | 65.30 | 65.30 | 8.10E-59 | M23 peptidase domain protein |
| AAur_0759 | AAur_0263 | 1647 bp | 1605 | 77.47 | 58.58 | 58.58 | 3.10E-47 | RNA polymerase sigma (70) factor putative |
| AAur_0766 | AAur_1259 | 645 bp | 651 | 96.59 | 56.44 | 56.44 | 8.20E-08 | membrane protein DedA family |
| AAur_0776 | AAur_0091 | 1221 bp | 2490 | 69.86 | 57.32 | 57.32 | 8.20E-22 | Acyltransferase family domain protein |
| AAur_0778 | AAur_3514 | 1944 bp | 1977 | 47.69 | 65.13 | 65.13 | 2.10E-111 | glycosyl transferase group 2 family protein |
| AAur_0842 | AAur_3852 | 1467 bp | 1494 | 95.64 | 79.76 | 79.76 | 5.20E-193 | D-serine/D-alanine/glycine transporter putative |
| AAur_0859 | AAur_1331 | 1494 bp | 1482 | 97.12 | 74.23 | 74.23 | 4.50E-167 | succinate-semialdehyde dehydrogenase putative |
| AAur_0860 | AAur_3583 | 888 bp | 894 | 99.89 | 70.54 | 70.54 | 6.10E-82 | transketolase subunit A putative |
| AAur_0861 | AAur_3584 | 942 bp | 933 | 97.98 | 64.97 | 64.97 | 4.00E-63 | transketolase subunit B putative |
| AAur_0864 | AAur_0868 | 966 bp | 888 | 92.24 | 59.93 | 59.93 | 1.90E-39 | NAD dependent epimerase/dehydratase family protein putative |
| AAur_0868 | AAur_0864 | 888 bp | 966 | 99.66 | 59.93 | 59.93 | 1.80E-39 | NAD dependent epimerase/dehydratase family protein putative |
| AAur_0880 | AAur_1678 | 459 bp | 447 | 94.34 | 68.41 | 68.41 | 2.60E-33 | transcriptional regulator MarR family putative |
| AAur_0973 | AAur_0045 | 1539 bp | 1503 | 93.96 | 68.84 | 68.84 | 3.40E-129 | amino acid permease putative |
| AAur_0975 | AAur_0313 | 1539 bp | 1518 | 50.10 | 77.89 | 77.89 | 6.60E-137 | conserved hypothetical protein |
| AAur_0987 | AAur_4007 | 2847 bp | 2865 | 99.89 | 81.21 | 81.21 | 0 | gcvP glycine dehydrogenase |
| AAur_0988 | AAur_4006 | 1134 bp | 1122 | 58.99 | 74.59 | 74.59 | 5.80E-146 | gcvT glycine cleavage system T protein |
| AAur_0989 | AAur_4005 | 387 bp | 384 | 98.45 | 77.98 | 77.98 | 4.30E-44 | gcvH glycine cleavage system H protein |
| AAur_0990 | AAur_4003 | 1413 bp | 1413 | 99.93 | 99.65 | 99.65 | 0 | sdaA L-serine ammonia-lyase |
| AAur_1018 | AAur_0675 | 837 bp | 1548 | 47.43 | 65.01 | 65.01 | 8.90E-25 | M23 peptidase domain protein |
| AAur_1019 | AAur_0408 | 789 bp | 795 | 84.54 | 58.42 | 58.42 | 5.50E-18 | hypothetical protein |
| AAur_1022 | AAur_0479 | 1359 bp | 1281 | 66.30 | 61.76 | 61.76 | 4.40E-42 | molybdopterin biosynthesis protein MoeA putative |
| AAur_1049 | AAur_1050 | 936 bp | 837 | 85.36 | 61.51 | 61.51 | 3.60E-41 | hypothetical protein |
| AAur_1050 | AAur_1049 | 837 bp | 936 | 96.18 | 61.51 | 61.51 | 3.20E-41 | hypothetical protein |
| AAur_1073 | AAur_2797 | 321 bp | 399 | 95.95 | 75.48 | 75.48 | 3.20E-32 | ferredoxin |
| AAur_1074 | AAur_pTC20226 | 744 bp | 780 | 50.94 | 63.82 | 63.82 | 1.20E-45 | two-component system response regulator putative |
| AAur_1118 | AAur_0224 | 912 bp | 2790 | 81.80 | 56.59 | 56.59 | 6.00E-12 | alpha-amylase family protein |
| AAur_1133 | AAur_1525 | 1896 bp | 1617 | 79.85 | 74.87 | 74.87 | 2.00E-161 | HNH endonuclease domain protein putative |
| AAur_1140 | AAur_pTC20131 | 1107 bp | 1128 | 94.76 | 65.60 | 65.60 | 7.40E-76 | oxidoreductase family NAD-binding Rossmann fold domain protein |
| AAur_1141 | AAur_pTC20130 | 963 bp | 930 | 90.03 | 61.67 | 61.67 | 7.90E-46 | conserved hypothetical protein |
| AAur_1145 | AAur_pTC20242 | 909 bp | 744 | 77.67 | 62.60 | 62.60 | 6.60E-40 | ANTAR domain protein |
| AAur_1171 | AAur_1324 | 648 bp | 567 | 77.93 | 61.58 | 61.58 | 2.20E-23 | hypothetical protein |
| AAur_1178 | AAur_4108 | 2604 bp | 2577 | 71.66 | 56.94 | 56.94 | 2.70E-90 | transcriptional regulator LuxR family domain protein putative |
| AAur_1185 | AAur_3505 | 1335 bp | 1437 | 67.34 | 57.39 | 57.39 | 8.70E-37 | hypothetical protein |
| AAur_1230 | AAur_1231 | 798 bp | 834 | 88.35 | 55.60 | 55.60 | 4.30E-11 | ABC-type multidrug transport system permease component putative |
| AAur_1231 | AAur_1230 | 834 bp | 798 | 84.53 | 55.60 | 55.60 | 4.50E-11 | ABC-2 type transporter putative |
| AAur_1259 | AAur_1260 | 651 bp | 693 | 82.03 | 61.31 | 61.31 | 2.00E-24 | membrane protein DedA family |
| AAur_1260 | AAur_1259 | 693 bp | 651 | 77.06 | 61.31 | 61.31 | 2.10E-24 | membrane protein DedA family |
| AAur_1288 | AAur_pTC10184 | 384 bp | 318 | 70.83 | 62.01 | 62.01 | 1.30E-08 | Hypothetical protein |
| AAur_1324 | AAur_1171 | 567 bp | 648 | 89.07 | 61.58 | 61.58 | 1.90E-23 | hypothetical protein |
| AAur_1328 | AAur_1687 | 1833 bp | 1812 | 48.99 | 63.23 | 63.23 | 2.40E-83 | long-chain-fatty-acid-CoA ligase putative |
| AAur_1331 | AAur_pTC20095 | 1482 bp | 1521 | 98.92 | 74.97 | 74.97 | 1.20E-171 | succinate-semialdehyde dehydrogenase putative |
| AAur_1365 | AAur_pTC20045 | 669 bp | 693 | 92.83 | 73.92 | 73.92 | 3.70E-67 | thioredoxin domain protein (DSBA) putative |
| AAur_1374 | AAur_pTC20149 | 425 bp | 579 | 92.94 | 65.35 | 65.35 | 4.40E-24 | adk Adenylate kinase (ATP-AMP transphosphorylase) |
| AAur_1375 | AAur_3353 | 2013 bp | 2058 | 98.71 | 70.95 | 70.95 | 2.00E-190 | acsA acetate--CoA ligase |
| AAur_1378 | AAur_pTC20192 | 1941 bp | 1935 | 99.38 | 80.03 | 80.03 | 6.80E-271 | amine oxidase (copper-containing) |
| AAur_1379 | AAur_3062 | 432 bp | 417 | 49.54 | 71.89 | 71.89 | 4.40E-31 | conserved hypothetical protein |
| AAur_1380 | AAur_pTC20196 | 1524 bp | 1524 | 99.87 | 80.75 | 80.75 | 2.10E-217 | aldehyde dehydrogenase |
| AAur_1383 | AAur_pTC20193 | 840 bp | 840 | 99.88 | 100.00 | 100.00 | 1.10E-183 | ISAau1 transposase orfB |
| AAur_1391 | AAur_0276 | 405 bp | 402 | 91.11 | 65.07 | 65.07 | 2.20E-22 | conserved hypothetical protein |
| AAur_1397 | AAur_3076 | 1035 bp | 1356 | 72.08 | 62.08 | 62.08 | 1.50E-35 | cobA uroporphyrin-III C-methyltransferase |
| AAur_1423 | AAur_1424 | 6135 bp | 6183 | 69.05 | 62.19 | 62.19 | 4.50E-244 | Unknown fibronectin like domains protein putative |
| AAur_1424 | AAur_1423 | 6183 bp | 6135 | 68.62 | 62.19 | 62.19 | 4.50E-244 | Unknown fibronectin like domains protein putative |
| AAur_1458 | AAur_2511 | 924 bp | 933 | 95.78 | 56.46 | 56.46 | 4.80E-26 | transcriptional regulator LysR family putative |
| AAur_1461 | AAur_1384 | 279 bp | 279 | 99.64 | 100.00 | 100.00 | 1.70E-56 | ISAau1 transposase orfA |
| AAur_1462 | AAur_1383 | 840 bp | 840 | 99.88 | 100.00 | 100.00 | 1.10E-183 | ISAau1 transposase orfB |
| AAur_1493 | AAur_2591 | 2499 bp | 2517 | 52.22 | 61.45 | 61.45 | 7.20E-72 | membrane protein putative |
| AAur_1525 | AAur_1133 | 1617 bp | 1896 | 93.69 | 74.87 | 74.87 | 1.70E-161 | HNH endonuclease domain protein putative |
| AAur_1548 | AAur_4138 | 729 bp | 723 | 99.04 | 59.21 | 59.21 | 4.70E-21 | lipoprotein putative |
| AAur_1563 | AAur_3398 | 483 bp | 495 | 84.06 | 67.39 | 67.39 | 2.70E-28 | cytidine/deoxycytidylate deaminase putative |
| AAur_1583 | AAur_pTC10147 | 1236 bp | 1344 | 80.10 | 62.20 | 62.20 | 8.50E-55 | Membrane protein putative |
| AAur_1645 | AAur_0149 | 129 bp | 123 | 62.02 | 97.53 | 97.53 | 1.30E-10 | hypothetical protein |
| AAur_1660 | AAur_pTC20150 | 1140 bp | 1143 | 97.81 | 80.98 | 80.98 | 2.70E-158 | gltA citrate synthase |
| AAur_1661 | AAur_pTC20151 | 906 bp | 906 | 99.89 | 81.92 | 81.92 | 1.20E-131 | prpB methylisocitrate lyase |
| AAur_1662 | AAur_pTC20152 | 1521 bp | 1521 | 99.93 | 83.32 | 83.32 | 7.60E-234 | 2-methylcitrate dehydratase putative |
| AAur_1663 | AAur_pTC20153 | 642 bp | 657 | 99.84 | 77.73 | 77.73 | 1.00E-80 | transcription regulator GntR family |
| AAur_1695 | AAur_3228 | 1338 bp | 1212 | 57.62 | 56.23 | 56.23 | 1.50E-07 | LysM domain protein putative |
| AAur_1740 | AAur_1383 | 840 bp | 840 | 99.88 | 100.00 | 100.00 | 1.10E-183 | ISAau1 transposase orfB |
| AAur_1741 | AAur_1384 | 279 bp | 279 | 99.64 | 100.00 | 100.00 | 1.70E-56 | ISAau1 transposase orfA |
| AAur_1747 | AAur_4002 | 1008 bp | 1008 | 99.70 | 98.71 | 98.71 | 5.80E-216 | lipA lipoic acid synthetase |
| AAur_1781 | AAur_pTC10181 | 471 bp | 525 | 89.17 | 64.00 | 64.00 | 2.20E-25 | Hypothetical protein |
| AAur_1850 | AAur_pTC10228 | 1581 bp | 1503 | 72.99 | 77.16 | 77.16 | 1.30E-199 | Multicopper oxidase family protein putative |
| AAur_1851 | AAur_pTC10230 | 414 bp | 1407 | 64.49 | 75.65 | 75.65 | 6.80E-27 | Metallo-beta-lactamase superfamily protein |
| AAur_1852 | AAur_1881 | 429 bp | 429 | 99.77 | 94.64 | 94.64 | 3.20E-81 | trx thioredoxin |
| AAur_1854 | AAur_1912 | 852 bp | 2385 | 98.24 | 65.11 | 65.11 | 2.00E-54 | aceE pyruvate dehydrogenase E1 component |
| AAur_1870 | AAur_1884 | 1044 bp | 1029 | 87.74 | 57.42 | 57.42 | 7.80E-31 | transcriptional regulator AraC family domain |
| AAur_1881 | AAur_1852 | 429 bp | 429 | 99.77 | 94.64 | 94.64 | 3.20E-81 | trx thioredoxin |
| AAur_1884 | AAur_1870 | 1029 bp | 1044 | 89.60 | 57.42 | 57.42 | 7.70E-31 | hypothetical protein |
| AAur_1885 | AAur_0719 | 1269 bp | 1236 | 89.05 | 62.61 | 62.61 | 4.90E-69 | transcriptional regulator ROK family putative |
| AAur_1893 | AAur_0715 | 1014 bp | 1002 | 98.42 | 97.70 | 97.70 | 2.60E-210 | conserved hypothetical protein |
| AAur_1902 | AAur_4154 | 903 bp | 900 | 90.37 | 59.45 | 59.45 | 1.40E-32 | diacylglycerol kinase catalytic domain putative |
| AAur_1911 | AAur_0263 | 1671 bp | 1605 | 74.63 | 58.90 | 58.90 | 1.30E-46 | RNA polymerase sigma (70) factor putative |
| AAur_1960 | AAur_0414 | 945 bp | 924 | 94.07 | 65.75 | 65.75 | 1.90E-63 | fructokinase |
| AAur_1992 | AAur_0713 | 1059 bp | 1023 | 93.48 | 58.67 | 58.67 | 7.80E-37 | oxidoreductase family NAD-binding Rossmann fold domain protein |
| AAur_2004 | AAur_pTC20183 | 1197 bp | 1197 | 99.75 | 62.43 | 62.43 | 3.10E-70 | pyridine nucleotide-disulphide oxidoreductase putative |
| AAur_2032 | AAur_pTC20088 | 1935 bp | 1917 | 98.66 | 78.71 | 78.71 | 2.20E-257 | FAD monooxygenase PheA/TfdB family putative |
| AAur_2093 | AAur_pTC20174 | 942 bp | 1094 | 97.77 | 78.34 | 78.34 | 1.90E-118 | opcA protein FRAMESHIFT |
| AAur_2117 | AAur_pTC20038 | 867 bp | 858 | 83.51 | 60.70 | 60.70 | 1.90E-35 | conserved hypothetical protein |
| AAur_2164 | AAur_3161 | 1410 bp | 1434 | 98.37 | 66.81 | 66.81 | 6.30E-110 | rfbC / rfbD dTDP-4-keto-L-rhamnose reductase and dTDP-4-keto-6-deoxyglucose-3 5-epimerase |
| AAur_2165 | AAur_3160 | 1005 bp | 1068 | 99.90 | 70.80 | 70.80 | 9.10E-94 | rfbB dTDP-glucose 4 6-dehydratase |
| AAur_2166 | AAur_3159 | 867 bp | 897 | 99.77 | 70.18 | 70.18 | 1.00E-81 | rfbA glucose-1-phosphate thymidylyltransferase |
| AAur_2207 | AAur_pTC10179 | 675 bp | 648 | 89.04 | 74.09 | 74.09 | 9.90E-65 | ctaE Cytochrome c oxidase subunit III |
| AAur_2221 | AAur_1114 | 1020 bp | 1032 | 94.02 | 57.83 | 57.83 | 9.40E-29 | hydrolase alpha/beta fold family domain putative |
| AAur_2306 | AAur_0487 | 1002 bp | 915 | 89.62 | 96.33 | 96.33 | 2.40E-183 | pyridoxine biosynthesis protein |
| AAur_2370 | AAur_pTC20035 | 879 bp | 672 | 74.18 | 71.92 | 71.92 | 3.20E-61 | integral membrane protein putative |
| AAur_2404 | AAur_0326 | 1572 bp | 1608 | 98.92 | 62.06 | 62.06 | 4.80E-85 | integral membrane protein putative |
| AAur_2425 | AAur_2869 | 2085 bp | 2103 | 98.90 | 65.98 | 65.98 | 9.90E-160 | Acyltransferase family domain protein |
| AAur_2433 | AAur_3391 | 2799 bp | 2778 | 99.21 | 98.88 | 98.88 | 0 | pyruvate dehydrogenase E1 component |
| AAur_2457 | AAur_pTC10266 | 2037 bp | 1737 | 80.61 | 79.21 | 79.21 | 1.10E-223 | thiC thiamine biosynthesis |
| AAur_2461 | AAur_3419 | 1422 bp | 1386 | 93.46 | 71.99 | 71.99 | 1.30E-134 | amt ammonium transporter |
| AAur_2465 | AAur_pTC20020 | 4914 bp | 4905 | 99.98 | 73.50 | 73.50 | 0 | hypothetical protein |
| AAur_2466 | AAur_pTC20019 | 1029 bp | 1047 | 99.90 | 73.96 | 73.96 | 1.90E-114 | conserved hypothetical protein |
| AAur_2516 | AAur_3204 | 468 bp | 417 | 81.62 | 64.36 | 64.36 | 1.90E-21 | transcriptional regulator MarR family putative |
| AAur_2540 | AAur_3154 | 360 bp | 1482 | 71.39 | 62.45 | 62.45 | 1.30E-10 | pyruvate dehydrogenase E2 (dihydrolipoamide acetyltransferase) |
| AAur_2550 | AAur_pTC10247 | 972 bp | 816 | 72.12 | 66.95 | 66.95 | 8.60E-50 | ABC transporter ATP-binding protein putative |
| AAur_2585 | AAur_3988 | 1293 bp | 1065 | 79.20 | 59.10 | 59.10 | 1.40E-38 | permease putative |
| AAur_2591 | AAur_1493 | 2517 bp | 2499 | 51.49 | 61.45 | 61.45 | 7.20E-72 | integral membrane protein (DUF470 DUF471 DUF472) putative |
| AAur_2728 | AAur_2729 | 273 bp | 810 | 76.19 | 98.56 | 98.56 | 1.10E-39 | transcription regulator GntR family |
| AAur_2823 | AAur_2054 | 936 bp | 909 | 91.03 | 57.34 | 57.34 | 2.30E-24 | BadF/BadG/BcrA/BcrD ATPase family protein putative |
| AAur_2845 | AAur_0599 | 681 bp | 681 | 99.71 | 99.56 | 99.56 | 3.10E-146 | hypothetical protein |
| AAur_2846 | AAur_3983 | 747 bp | 693 | 88.22 | 97.73 | 97.73 | 1.60E-136 | hypothetical protein |
| AAur_2847 | AAur_3982 | 375 bp | 417 | 95.47 | 65.22 | 65.22 | 2.50E-19 | hypothetical protein |
| AAur_2868 | AAur_2869 | 2085 bp | 2103 | 90.94 | 59.58 | 59.58 | 2.10E-76 | acyltransferase family domain protein |
| AAur_2869 | AAur_2425 | 2103 bp | 2085 | 97.77 | 65.98 | 65.98 | 1.00E-159 | acyltransferase family domain protein putative |
| AAur_2873 | AAur_1665 | 1635 bp | 1656 | 73.76 | 54.93 | 54.93 | 2.20E-12 | transcriptional regulator LuxR family putative |
| AAur_2928 | AAur_pTC20149 | 579 bp | 579 | 94.47 | 79.05 | 79.05 | 1.60E-70 | adk Adenylate kinase (ATP-AMP transphosphorylase) |
| AAur_2990 | AAur_3456 | 321 bp | 645 | 82.24 | 67.78 | 67.78 | 4.90E-17 | hypothetical protein |
| AAur_2996 | AAur_3401 | 1104 bp | 939 | 75.09 | 57.96 | 57.96 | 1.60E-24 | conserved hypothetical protein |
| AAur_3000 | AAur_4177 | 1344 bp | 1188 | 46.43 | 60.65 | 60.65 | 5.80E-26 | major facilitator superfamily (MFS) transporter putative |
| AAur_3027 | AAur_0973 | 1527 bp | 1539 | 59.53 | 60.17 | 60.17 | 5.40E-61 | amino acid permease putative |
| AAur_3045 | AAur_pTC10232 | 378 bp | 363 | 90.48 | 73.91 | 73.91 | 7.40E-34 | trx Thioredoxin |
| AAur_3062 | AAur_1379 | 417 bp | 432 | 51.32 | 71.89 | 71.89 | 4.20E-31 | protein of unknown function (DUF779) putative |
| AAur_3064 | AAur_pTC20196 | 1524 bp | 1524 | 99.93 | 83.75 | 83.75 | 5.80E-237 | aldehyde dehydrogenase |
| AAur_3066 | AAur_3075 | 1440 bp | 1467 | 97.64 | 76.28 | 76.28 | 1.70E-170 | ferredoxin/ferredoxin-NADP reductase putative |
| AAur_3075 | AAur_3066 | 1467 bp | 1440 | 96.25 | 76.28 | 76.28 | 1.70E-170 | ferredoxin/ferredoxin-NADP reductase putative |
| AAur_3084 | AAur_pTC20026 | 636 bp | 612 | 94.97 | 69.23 | 69.23 | 4.90E-48 | secreted protein with unknown function DUF305-family putative |
| AAur_3094 | AAur_3095 | 537 bp | 477 | 62.94 | 72.29 | 72.29 | 2.20E-27 | hypothetical protein |
| AAur_3095 | AAur_3094 | 477 bp | 537 | 70.86 | 72.29 | 72.29 | 2.00E-27 | hypothetical protein |
| AAur_3110 | AAur_1383 | 840 bp | 840 | 99.88 | 100.00 | 100.00 | 1.10E-183 | ISAau1 transposase orfB |
| AAur_3111 | AAur_1384 | 279 bp | 279 | 99.64 | 100.00 | 100.00 | 1.70E-56 | ISAau1 transposase orfA |
| AAur_3112 | AAur_1384 | 279 bp | 279 | 99.64 | 100.00 | 100.00 | 1.70E-56 | ISAau1 transposase orfA |
| AAur_3113 | AAur_1383 | 840 bp | 840 | 99.88 | 100.00 | 100.00 | 1.10E-183 | ISAau1 transposase orfB |
| AAur_3119 | AAur_1958 | 1398 bp | 1551 | 45.49 | 59.25 | 59.25 | 1.10E-14 | glycosyl hydrolases family 32 protein putative |
| AAur_3126 | AAur_3209 | 2820 bp | 2709 | 40.00 | 66.52 | 66.52 | 1.10E-194 | serine protease subtilase family putative |
| AAur_3132 | AAur_3144 | 2946 bp | 2979 | 56.14 | 93.20 | 93.20 | 0 | S-layer domain protein putative |
| AAur_3133 | AAur_3141 | 240 bp | 237 | 99.58 | 97.08 | 97.08 | 5.70E-44 | hypothetical protein |
| AAur_3141 | AAur_3133 | 237 bp | 240 | 99.58 | 97.08 | 97.08 | 5.60E-44 | hypothetical protein |
| AAur_3144 | AAur_3132 | 2979 bp | 2946 | 55.59 | 93.20 | 93.20 | 0 | S-layer domain protein putative |
| AAur_3149 | AAur_0503 | 789 bp | 789 | 99.87 | 78.06 | 78.06 | 3.90E-100 | hypothetical protein |
| AAur_3152 | AAur_1383 | 840 bp | 840 | 99.88 | 100.00 | 100.00 | 1.10E-183 | ISAau1 transposase orfB |
| AAur_3153 | AAur_1384 | 279 bp | 279 | 99.64 | 100.00 | 100.00 | 1.70E-56 | ISAau1 transposase orfA |
| AAur_3161 | AAur_2164 | 1434 bp | 1410 | 96.93 | 66.81 | 66.81 | 6.40E-110 | rfbC / rfbD dTDP-4-keto-L-rhamnose reductase /dTDP-4-keto-6-deoxyglucose-3 5-epimerase |
| AAur_3169 | AAur_4125 | 1365 bp | 1491 | 92.89 | 61.44 | 61.44 | 2.60E-66 | glucosyltransferase putative |
| AAur_3173 | AAur_1383 | 840 bp | 840 | 99.88 | 100.00 | 100.00 | 1.10E-183 | ISAau1 transposase orfB |
| AAur_3175 | AAur_1384 | 306 bp | 279 | 90.85 | 100.00 | 100.00 | 1.70E-56 | ISAau1 transposase orfA |
| AAur_3203 | AAur_3840 | 1221 bp | 1218 | 76.09 | 71.05 | 71.05 | 5.60E-91 | FG-GAP repeat domain protein |
| AAur_3204 | AAur_2516 | 417 bp | 468 | 91.61 | 64.36 | 64.36 | 1.70E-21 | transcriptional regulator MarR family putative |
| AAur_3209 | AAur_3126 | 2709 bp | 2820 | 41.42 | 66.52 | 66.52 | 1.10E-194 | serine protease subtilase family putative |
| AAur_3210 | AAur_3247 | 2160 bp | 1911 | 48.66 | 72.24 | 72.24 | 1.50E-108 | S-layer domain protein putative |
| AAur_3212 | AAur_3136 | 1272 bp | 2526 | 43.55 | 70.28 | 70.28 | 6.60E-49 | S-layer domain protein putative |
| AAur_3214 | AAur_3134 | 1191 bp | 2958 | 51.30 | 64.08 | 64.08 | 1.00E-34 | S-layer domain protein putative |
| AAur_3215 | AAur_3220 | 1527 bp | 1551 | 96.20 | 60.29 | 60.29 | 1.70E-75 | lipoprotein putative |
| AAur_3219 | AAur_0181 | 165 bp | 153 | 86.67 | 68.97 | 68.97 | 4.60E-06 | hypothetical protein |
| AAur_3220 | AAur_3215 | 1551 bp | 1527 | 95.04 | 60.29 | 60.29 | 1.70E-75 | lipoprotein putative |
| AAur_3240 | AAur_3382 | 1131 bp | 1266 | 89.83 | 59.31 | 59.31 | 1.90E-41 | Helix-turn-helix domain protein putative |
| AAur_3242 | AAur_0628 | 1554 bp | 1419 | 90.35 | 65.54 | 65.54 | 2.10E-106 | GGDEF domain/EAL domain protein putative |
| AAur_3247 | AAur_3210 | 1911 bp | 2160 | 54.68 | 72.24 | 72.24 | 1.30E-108 | S-layer domain protein putative |
| AAur_3287 | AAur_pTC20039 | 1695 bp | 1632 | 93.45 | 72.23 | 72.23 | 1.10E-168 | cytochrome c biogenesis membrane protein |
| AAur_3289 | AAur_pTC10198 | 606 bp | 630 | 94.22 | 68.43 | 68.43 | 9.30E-45 | Thiol disulfide interchange protein thioredoxin family |
| AAur_3298 | AAur_1384 | 279 bp | 279 | 99.64 | 100.00 | 100.00 | 1.70E-56 | ISAau1 transposase orfA |
| AAur_3299 | AAur_1383 | 840 bp | 840 | 99.88 | 100.00 | 100.00 | 1.10E-183 | ISAau1 transposase orfB |
| AAur_3304 | AAur_3315 | 2100 bp | 2049 | 61.52 | 59.22 | 59.22 | 1.40E-82 | oxidoreductase family NAD-binding Rossmann fold domain protein |
| AAur_3312 | AAur_pTC20133 | 942 bp | 933 | 93.42 | 73.57 | 73.57 | 1.20E-94 | sugar ABC transporter permease protein putative |
| AAur_3315 | AAur_3304 | 2049 bp | 2100 | 62.66 | 59.22 | 59.22 | 1.30E-82 | oxidoreductase family NAD-binding Rossmann fold domain protein |
| AAur_3353 | AAur_pTC20189 | 2058 bp | 2031 | 35.42 | 82.90 | 82.90 | 7.20E-207 | acsA acetate--CoA ligase |
| AAur_3369 | AAur_3416 | 2289 bp | 2190 | 73.26 | 63.23 | 63.23 | 3.10E-143 | penicillin-binding protein |
| AAur_3382 | AAur_3240 | 1266 bp | 1131 | 80.33 | 59.31 | 59.31 | 2.10E-41 | hypothetical protein |
| AAur_3391 | AAur_2433 | 2778 bp | 2799 | 99.96 | 98.88 | 98.88 | 0 | pyruvate dehydrogenase E1 component |
| AAur_3400 | AAur_0587 | 1539 bp | 1149 | -41.98 | 58.47 | 58.47 | 3.80E-17 | lipoprotein putative |
| AAur_3401 | AAur_3563 | 939 bp | 948 | 97.02 | 62.69 | 62.69 | 3.70E-52 | conserved hypothetical protein |
| AAur_3416 | AAur_3369 | 2190 bp | 2289 | 76.90 | 63.23 | 63.23 | 3.00E-143 | penicillin-binding protein |
| AAur_3419 | AAur_2461 | 1386 bp | 1422 | 95.45 | 71.99 | 71.99 | 1.20E-134 | amt ammonium transporter |
| AAur_3422 | AAur_3839 | 378 bp | 1146 | 62.70 | 66.67 | 66.67 | 1.10E-13 | FG-GAP repeat domain protein |
| AAur_3426 | AAur_2928 | 642 bp | 579 | 90.03 | 65.71 | 65.71 | 6.10E-38 | adk Adenylate kinase (ATP-AMP transphosphorylase) |
| AAur_3456 | AAur_2990 | 645 bp | 321 | 41.09 | 67.78 | 67.78 | 9.90E-17 | hypothetical protein |
| AAur_3457 | AAur_3455 | 156 bp | 921 | -93.59 | 74.50 | 74.50 | 4.20E-09 | conserved hypothetical protein |
| AAur_3467 | AAur_0584 | 837 bp | 834 | 99.88 | 74.61 | 74.61 | 5.00E-93 | haloperoxidase |
| AAur_3505 | AAur_1185 | 1437 bp | 1335 | 62.42 | 57.39 | 57.39 | 9.40E-37 | hypothetical protein |
| AAur_3514 | AAur_0778 | 1977 bp | 1944 | 47.14 | 65.13 | 65.13 | 2.10E-111 | glycosyl transferase group 2 family domain protein |
| AAur_3545 | AAur_3544 | 369 bp | 315 | 84.01 | 63.29 | 63.29 | 4.70E-15 | multidrug resistance protein (sugE) putative |
| AAur_3563 | AAur_3401 | 948 bp | 939 | 95.68 | 62.69 | 62.69 | 3.70E-52 | conserved hypothetical protein |
| AAur_3583 | AAur_0860 | 894 bp | 888 | 99.33 | 70.54 | 70.54 | 6.10E-82 | transketolase subunit A putative |
| AAur_3584 | AAur_0861 | 933 bp | 942 | 99.04 | 64.97 | 64.97 | 3.90E-63 | transketolase subunit B putative |
| AAur_3588 | AAur_3554 | 1302 bp | 1293 | 89.48 | 58.94 | 58.94 | 4.60E-46 | major facilitator superfamily (MFS) transporter putative |
| AAur_3602 | AAur_pTC20259 | 459 bp | 798 | 65.80 | 80.26 | 80.26 | 6.00E-38 | Protein of unknown function (DUF1016) |
| AAur_3618 | AAur_2813 | 1266 bp | 1221 | 94.63 | 71.02 | 71.02 | 1.70E-116 | Aspartate aminotransferase putative |
| AAur_3624 | AAur_pTC20083 | 591 bp | 594 | 98.98 | 69.65 | 69.65 | 4.70E-51 | transcription regulator putative |
| AAur_3626 | AAur_pTC20102 | 1413 bp | 1410 | 99.93 | 77.28 | 77.28 | 2.50E-180 | Aldehyde dehydrogenase family protein putative |
| AAur_3628 | AAur_pTC20060 | 1380 bp | 1530 | 98.70 | 76.06 | 76.06 | 1.70E-166 | Secreted protein putative |
| AAur_3629 | AAur_pTC20059 | 978 bp | 990 | 99.28 | 81.07 | 81.07 | 9.20E-139 | trxB thioredoxin-disulfide reductase |
| AAur_3630 | AAur_pTC20058 | 423 bp | 423 | 99.76 | 83.73 | 83.73 | 7.60E-61 | arsC Arsenate reductase |
| AAur_3631 | AAur_pTC20057 | 375 bp | 675 | 99.20 | 68.63 | 68.63 | 1.50E-30 | transcriptional regulator ArsR family/phosphatase family protein |
| AAur_3634 | AAur_pTC20055 | 378 bp | 348 | 92.59 | 75.42 | 75.42 | 9.80E-36 | transcriptional regulator ArsR family putative |
| AAur_3635 | AAur_pTC20054 | 510 bp | 489 | 95.10 | 69.72 | 69.72 | 3.20E-40 | phosphinothricin N-acetyltransferase putative |
| AAur_3658 | AAur_3038 | 1074 bp | 1386 | 93.85 | 60.66 | 60.66 | 4.00E-48 | cystathionine beta-synthase |
| AAur_3687 | AAur_2665 | 1179 bp | 2748 | 64.04 | 56.37 | 56.37 | 1.10E-10 | lysozyme like protein putative |
| AAur_3708 | AAur_0534 | 1023 bp | 984 | 95.99 | 68.17 | 68.17 | 1.00E-81 | transcriptional regulator lacI family putative |
| AAur_3712 | AAur_0353 | 897 bp | 906 | 99.67 | 62.66 | 62.66 | 4.50E-48 | aldose-1-epimerase putative |
| AAur_3720 | AAur_pTC20125 | 432 bp | 426 | 97.22 | 77.73 | 77.73 | 3.10E-50 | panD aspartate 1-decarboxylase |
| AAur_3727 | AAur_3203 | 1206 bp | 1221 | 69.32 | 64.84 | 64.84 | 4.50E-55 | FG-GAP repeat domain protein |
| AAur_3730 | AAur_3440 | 1068 bp | 1152 | 78.93 | 59.52 | 59.52 | 1.30E-31 | monooxygenase putative |
| AAur_3733 | AAur_3724 | 993 bp | 993 | 96.58 | 63.71 | 63.71 | 2.10E-58 | ABC transporter solute binding protein putative |
| AAur_3742 | AAur_pTC20042 | 2256 bp | 2106 | 91.93 | 58.43 | 58.43 | 7.50E-77 | copper resistance membrane protein (CopD) putative |
| AAur_3757 | AAur_3759 | 765 bp | 819 | 96.73 | 63.32 | 63.32 | 3.40E-43 | GDSL-like Lipase/Acylhydrolase domain protein |
| AAur_3759 | AAur_3761 | 819 bp | 837 | 91.82 | 63.49 | 63.49 | 1.60E-45 | GDSL-like Lipase/Acylhydrolase domain protein |
| AAur_3761 | AAur_3759 | 837 bp | 819 | 89.73 | 63.49 | 63.49 | 1.70E-45 | GDSL-like Lipase/Acylhydrolase domain protein |
| AAur_3776 | AAur_1437 | 1035 bp | 1035 | 99.03 | 63.73 | 63.73 | 6.90E-65 | oxidoreductase aldo/keto reductase family |
| AAur_3788 | AAur_0446 | 939 bp | 1032 | 92.55 | 90.87 | 90.87 | 4.80E-155 | hypothetical protein |
| AAur_3804 | AAur_3839 | 1074 bp | 1146 | 55.96 | 62.46 | 62.46 | 8.00E-33 | FG-GAP repeat domain protein |
| AAur_3824 | AAur_0486 | 672 bp | 681 | 98.66 | 63.76 | 63.76 | 1.20E-39 | transcriptional regulator GntR family |
| AAur_3825 | AAur_4004 | 1389 bp | 1320 | 86.61 | 79.60 | 79.60 | 1.90E-163 | glyA serine hydroxymethyltransferase |
| AAur_3838 | AAur_3839 | 1203 bp | 1146 | 92.44 | 64.30 | 64.30 | 1.70E-71 | FG-GAP repeat domain protein |
| AAur_3839 | AAur_3838 | 1146 bp | 1203 | 97.03 | 64.30 | 64.30 | 1.70E-71 | FG-GAP repeat domain protein |
| AAur_3840 | AAur_3203 | 1218 bp | 1221 | 76.85 | 71.05 | 71.05 | 5.60E-91 | FG-GAP repeat domain protein |
| AAur_3852 | AAur_0842 | 1494 bp | 1467 | 94.11 | 79.76 | 79.76 | 5.30E-193 | D-serine/D-alanine/glycine transporter putative |
| AAur_3862 | AAur_0293 | 279 bp | 276 | 98.57 | 77.70 | 77.70 | 5.50E-30 | phosphocarrier protein HPr |
| AAur_3870 | AAur_pTC20204 | 1119 bp | 1116 | 99.91 | 80.30 | 80.30 | 3.30E-155 | ald Alanine dehydrogenase |
| AAur_3872 | AAur_0606 | 573 bp | 555 | 93.89 | 62.11 | 62.11 | 4.40E-28 | RNA polymerase sigma (70) factor putative |
| AAur_3873 | AAur_0596 | 1050 bp | 1062 | 92.95 | 61.92 | 61.92 | 2.00E-54 | oxidoreductase family NAD-binding Rossmann fold domain protein |
| AAur_3886 | AAur_0235 | 429 bp | 432 | 97.67 | 64.79 | 64.79 | 1.10E-25 | universal stress family domain protein putative |
| AAur_3893 | AAur_1521 | 1191 bp | 1098 | 53.74 | 64.35 | 64.35 | 6.80E-61 | pyruvate dehydrogenase E1 component alpha subunit |
| AAur_3912 | AAur_pTC20094 | 804 bp | 804 | 97.39 | 71.83 | 71.83 | 1.70E-79 | 2 4-dihydroxyhept-2-ene-1 7-dioic acid aldolase (HpaI) putative |
| AAur_3913 | AAur_pTC20093 | 786 bp | 687 | 86.51 | 84.48 | 84.48 | 5.80E-104 | 2-oxo-hepta-3-ene-1 7-dioic acid hydratase putative |
| AAur_3916 | AAur_pTC20090 | 678 bp | 681 | 96.76 | 80.18 | 80.18 | 1.50E-88 | Transcriptional regulator gntR-family putative |
| AAur_3917 | AAur_pTC20089 | 1470 bp | 1413 | 94.08 | 78.16 | 78.16 | 1.20E-175 | Dimethylmenaquinone methyltransferase putative |
| AAur_3922 | AAur_3992 | 270 bp | 261 | 95.93 | 68.58 | 68.58 | 1.50E-18 | integral membrane protein putative |
| AAur_3926 | AAur_1094 | 936 bp | 924 | 92.31 | 64.95 | 64.95 | 2.30E-61 | oxidoreductase short chain dehydrogenase/reductase family |
| AAur_3959 | AAur_0973 | 1563 bp | 1539 | 93.73 | 56.47 | 56.47 | 2.30E-31 | amino acid permease putative |
| AAur_3974 | AAur_2039 | 885 bp | 918 | 94.92 | 57.54 | 57.54 | 2.80E-24 | transcriptional regulator putative |
| AAur_3978 | AAur_1525 | 1947 bp | 1617 | 67.23 | 75.50 | 75.50 | 3.90E-143 | HNH endonuclease domain protein putative |
| AAur_3982 | AAur_2847 | 417 bp | 375 | 85.37 | 65.22 | 65.22 | 2.80E-19 | hypothetical protein |
| AAur_3983 | AAur_2846 | 693 bp | 747 | 95.09 | 97.73 | 97.73 | 1.50E-136 | hypothetical protein |
| AAur_3984 | AAur_0599 | 636 bp | 681 | 99.84 | 99.37 | 99.37 | 6.80E-136 | hypothetical protein |
| AAur_3988 | AAur_2585 | 1065 bp | 1293 | 96.43 | 59.10 | 59.10 | 1.20E-38 | permease putative |
| AAur_3992 | AAur_3922 | 261 bp | 270 | 99.23 | 68.58 | 68.58 | 1.50E-18 | integral membrane protein putative |
| AAur_3993 | AAur_3950 | 831 bp | 1344 | 79.42 | 58.13 | 58.13 | 7.90E-18 | serine/threonine protein kinase domain protein putative |
| AAur_3995 | AAur_0316 | 540 bp | 573 | 98.52 | 68.99 | 68.99 | 1.10E-41 | transcriptional regulator MarR family putative |
| AAur_4002 | AAur_1747 | 1008 bp | 1008 | 99.70 | 98.71 | 98.71 | 5.80E-216 | lipA lipoic acid synthetase |
| AAur_4003 | AAur_0990 | 1413 bp | 1413 | 99.93 | 99.65 | 99.65 | 2.8e-311 | sdaA L-serine ammonia-lyase |
| AAur_4004 | AAur_3825 | 1320 bp | 1389 | 91.14 | 79.60 | 79.60 | 1.80E-163 | glyA serine hydroxymethyltransferase |
| AAur_4005 | AAur_0989 | 384 bp | 387 | 99.22 | 77.98 | 77.98 | 4.30E-44 | gcvH glycine cleavage system H protein |
| AAur_4006 | AAur_0988 | 1122 bp | 1134 | 59.63 | 74.59 | 74.59 | 5.70E-146 | gcvT glycine cleavage system T protein |
| AAur_4007 | AAur_0987 | 2865 bp | 2847 | 99.48 | 81.21 | 81.21 | 0 | gcvP glycine dehydrogenase |
| AAur_4033 | AAur_0330 | 855 bp | 837 | 92.98 | 64.79 | 64.79 | 1.90E-49 | 2-hydroxyhepta-2 4-diene-1 7-dioate isomerase putative |
| AAur_4053 | AAur_0626 | 1203 bp | 1179 | 94.10 | 66.55 | 66.55 | 2.50E-91 | GGDEF domain protein putative |
| AAur_4094 | AAur_pTC10138 | 234 bp | 336 | 78.63 | 66.84 | 66.84 | 3.60E-08 | COP associated protein (Copper ion binding protein) |
| AAur_4108 | AAur_1178 | 2577 bp | 2604 | 72.37 | 56.94 | 56.94 | 2.60E-90 | transcriptional regulator LuxR family putative |
| AAur_4125 | AAur_3169 | 1491 bp | 1365 | 85.04 | 61.44 | 61.44 | 2.80E-66 | glucosyltransferase putative |
| AAur_4135 | AAur_1102 | 1737 bp | 1755 | 91.83 | 64.12 | 64.12 | 1.50E-97 | dihydroxyacetone kinase L subunit putative |
| AAur_4138 | AAur_1548 | 723 bp | 729 | 99.45 | 59.21 | 59.21 | 4.60E-21 | lipoprotein putative |
| AAur_4154 | AAur_1902 | 900 bp | 903 | 90.78 | 59.45 | 59.45 | 1.40E-32 | diacylglycerol kinase catalytic domain putative |
| AAur_4164 | AAur_pTC20073 | 582 bp | 516 | 85.05 | 74.30 | 74.30 | 5.50E-52 | ssb single-strand binding protein |
| AAur_pTC10001 | AAur_pTC10093 | 1122 bp | 942 | 55.79 | 57.53 | 57.53 | 1.10E-20 | Putative Soj/ParA family protein |
| AAur_pTC10018 | AAur_0827 | 447 bp | 2112 | -85.46 | 61.76 | 61.76 | 3.40E-08 | ABC-type dipeptide/oligopeptide transport system ATPase component putative |
| AAur_pTC10058 | AAur_pTC20218 | 1116 bp | 1002 | 87.46 | 67.81 | 67.81 | 3.10E-79 | Alcohol dehydrogenase putative |
| AAur_pTC10061 | AAur_pTC20219 | 1473 bp | 1497 | 97.42 | 60.50 | 60.50 | 1.10E-65 | aldehyde dehydrogenase (NAD) family protein |
| AAur_pTC10062 | AAur_pTC20206 | 732 bp | 765 | 93.99 | 61.74 | 61.74 | 5.20E-32 | glutamine amidotransferase putative |
| AAur_pTC10063 | AAur_pTC20215 | 1353 bp | 1368 | 99.93 | 66.79 | 66.79 | 1.30E-105 | aldehyde dehydrogenase |
| AAur_pTC10064 | AAur_pTC20212 | 774 bp | 774 | 99.87 | 69.64 | 69.64 | 7.90E-69 | oxidoreductase short chain dehydrogenase/reductase family |
| AAur_pTC10065 | AAur_pTC20211 | 726 bp | 741 | 95.87 | 64.76 | 64.76 | 2.60E-41 | glutamine amidotransferase putative |
| AAur_pTC10066 | AAur_pTC20210 | 1164 bp | 1194 | 85.31 | 63.54 | 63.54 | 6.40E-56 | Cytochrome P450 putative |
| AAur_pTC10068 | AAur_pTC20208 | 1167 bp | 1107 | 93.32 | 58.16 | 58.16 | 3.60E-33 | ferredoxin reductase putative |
| AAur_pTC10069 | AAur_pTC20213 | 1419 bp | 1392 | 83.02 | 69.26 | 69.26 | 8.50E-107 | ipuC gamma-glutamylisopropylamide synthetase |
| AAur_pTC10071 | AAur_pTC20207 | 1905 bp | 1785 | 52.70 | 57.84 | 57.84 | 2.20E-27 | GAF domain protein |
| AAur_pTC10082 | AAur_pTC10089 | 1131 bp | 1131 | 100.00 | 100.00 | 100.00 | 1.60E-249 | hypothetical protein |
| AAur_pTC10083 | AAur_pTC10090 | 897 bp | 873 | 97.21 | 100.00 | 100.00 | 3.80E-191 | Hypothetical protein |
| AAur_pTC10084 | AAur_pTC10091 | 6330 bp | 5127 | 79.00 | 99.46 | 99.46 | 0 | Toprim domain protein |
| AAur_pTC10089 | AAur_pTC10082 | 1131 bp | 1131 | 99.91 | 100.00 | 100.00 | 1.60E-249 | hypothetical protein |
| AAur_pTC10090 | AAur_pTC10083 | 873 bp | 873 | 99.89 | 100.00 | 100.00 | 3.80E-191 | Hypothetical protein |
| AAur_pTC10091 | AAur_pTC10084 | 5127 bp | 6330 | 97.50 | 99.46 | 99.46 | 0 | Toprim domain |
| AAur_pTC10093 | AAur_pTC10001 | 942 bp | 1122 | 66.45 | 57.53 | 57.53 | 8.90E-21 | ATPase ParA-family putative |
| AAur_pTC10095 | AAur_pTC20273 | 1926 bp | 1878 | 83.23 | 55.96 | 55.96 | 9.60E-29 | conserved hypothetical protein |
| AAur_pTC10098 | AAur_0076 | 453 bp | 450 | 98.45 | 88.37 | 88.37 | 1.60E-73 | conserved hypothetical protein |
| AAur_pTC10099 | AAur_0075 | 450 bp | 447 | 98.22 | 90.77 | 90.77 | 9.10E-77 | conserved hypothetical protein |
| AAur_pTC10100 | AAur_1383 | 840 bp | 840 | 99.88 | 100.00 | 100.00 | 1.10E-183 | ISAau1 transposase orfB |
| AAur_pTC10101 | AAur_0074 | 321 bp | 858 | 77.57 | 83.00 | 83.00 | 5.10E-32 | integral membrane protein putative |
| AAur_pTC10102 | AAur_1384 | 279 bp | 279 | 99.64 | 100.00 | 100.00 | 1.70E-56 | ISAau1 transposase orfA |
| AAur_pTC10103 | AAur_0074 | 606 bp | 858 | 99.84 | 85.97 | 85.97 | 2.70E-96 | integral membrane protein putative |
| AAur_pTC10104 | AAur_0073 | 1146 bp | 1122 | 97.47 | 81.79 | 81.79 | 5.00E-163 | conserved hypothetical protein |
| AAur_pTC10105 | AAur_pTC20271 | 585 bp | 1077 | 97.27 | 77.78 | 77.78 | 1.10E-69 | CHAP domain family protein |
| AAur_pTC10106 | AAur_pTC20271 | 1077 bp | 1077 | 53.48 | 63.68 | 63.68 | 1.50E-29 | CHAP domain family protein |
| AAur_pTC10108 | AAur_pTC20269 | 1194 bp | 1524 | 90.45 | 54.79 | 54.79 | 2.20E-14 | conserved hypothetical protein |
| AAur_pTC10121 | AAur_pTC10080 | 4452 bp | 3477 | 71.07 | 57.19 | 57.19 | 5.20E-82 | TraA-like conjugal transfer protein putative |
| AAur_pTC10124 | AAur_pTC20069 | 1686 bp | 1341 | 79.06 | 77.44 | 77.44 | 8.40E-168 | partitioning protein parB-family putative |
| AAur_pTC10125 | AAur_pTC20068 | 1266 bp | 1266 | 99.92 | 100.00 | 100.00 | 4.60E-280 | IS110 family transposase |
| AAur_pTC10126 | AAur_pTC20067 | 366 bp | 285 | 77.60 | 100.00 | 100.00 | 7.20E-58 | conserved hypothetical protein |
| AAur_pTC10132 | AAur_pTC20165 | 2322 bp | 2313 | 99.61 | 78.92 | 78.92 | 4.6e-314 | copper-translocating P-type ATPase |
| AAur_pTC10139 | AAur_pTC20028 | 2157 bp | 2142 | 98.66 | 74.28 | 74.28 | 4.00E-242 | copper-translocating P-type ATPase |
| AAur_pTC10141 | AAur_pTC20026 | 600 bp | 612 | 99.50 | 67.10 | 67.10 | 6.90E-44 | secreted protein with unknown function DUF305-family putative |
| AAur_pTC10142 | AAur_pTC20025 | 891 bp | 879 | 89.90 | 64.69 | 64.69 | 1.10E-47 | BNR/Asp-box repeat domain protein |
| AAur_pTC10143 | AAur_pTC10175 | 720 bp | 879 | 79.44 | 59.59 | 59.59 | 5.70E-20 | Hypothetical protein |
| AAur_pTC10144 | AAur_pTC10197 | 723 bp | 750 | 99.72 | 79.67 | 79.67 | 6.60E-97 | ccdA cytochrome c biogenesis membrane protein |
| AAur_pTC10145 | AAur_pTC20034 | 522 bp | 597 | 94.06 | 67.54 | 67.54 | 1.10E-37 | lipoprotein putative |
| AAur_pTC10147 | AAur_1583 | 1344 bp | 1236 | 73.88 | 62.20 | 62.20 | 9.30E-55 | integral membrane protein putative |
| AAur_pTC10151 | AAur_0657 | 303 bp | 300 | 99.67 | 73.03 | 73.03 | 1.70E-27 | conserved hypothetical protein |
| AAur_pTC10158 | AAur_pTC10171 | 321 bp | 1581 | 84.42 | 66.06 | 66.06 | 7.80E-15 | Tat domain protein |
| AAur_pTC10159 | AAur_pTC10171 | 297 bp | 1581 | 98.99 | 65.66 | 65.66 | 8.60E-18 | Tat domain protein |
| AAur_pTC10168 | AAur_pTC20050 | 1251 bp | 1254 | 99.76 | 75.54 | 75.54 | 4.80E-147 | mntH H+-stimulated Mn2+/Fe2+ transporter NRAMP family |
| AAur_pTC10174 | AAur_pTC20039 | 1626 bp | 1632 | 96.43 | 68.97 | 68.97 | 5.70E-140 | cytochrome c biogenesis membrane protein |
| AAur_pTC10175 | AAur_pTC10143 | 879 bp | 720 | 64.39 | 59.59 | 59.59 | 7.00E-20 | acetyltransferase GNAT family protein |
| AAur_pTC10178 | AAur_pTC20036 | 825 bp | 837 | 99.39 | 58.59 | 58.59 | 7.40E-27 | M23 peptidase domain protein |
| AAur_pTC10179 | AAur_2207 | 648 bp | 675 | 92.75 | 74.09 | 74.09 | 9.50E-65 | Cytochrome c oxidase subunit III |
| AAur_pTC10181 | AAur_1781 | 525 bp | 471 | 80.00 | 64.00 | 64.00 | 2.40E-25 | conserved hypothetical protein |
| AAur_pTC10184 | AAur_1288 | 318 bp | 384 | 83.65 | 62.01 | 62.01 | 1.10E-08 | conserved hypothetical protein |
| AAur_pTC10187 | AAur_pTC20032 | 513 bp | 516 | 96.10 | 70.48 | 70.48 | 4.90E-44 | lspA lipoprotein signal peptidase |
| AAur_pTC10188 | AAur_pTC20031 | 1959 bp | 1956 | 99.95 | 79.62 | 79.62 | 4.20E-268 | cadA cadmium-translocating P-type ATPase |
| AAur_pTC10189 | AAur_pTC20030 | 402 bp | 387 | 92.54 | 76.05 | 76.05 | 5.00E-40 | transcriptional regulator ArsR family putative |
| AAur_pTC10190 | AAur_pTC20043 | 441 bp | 378 | 75.74 | 73.29 | 73.29 | 4.60E-32 | transcriptional regulator ArsR family putative |
| AAur_pTC10191 | AAur_pTC20044 | 807 bp | 918 | 99.01 | 76.53 | 76.53 | 6.00E-98 | czc cobalt-zinc-cadmium efflux permease |
| AAur_pTC10192 | AAur_pTC20045 | 657 bp | 693 | 92.69 | 69.03 | 69.03 | 1.20E-49 | thioredoxin domain protein (DSBA) putative |
| AAur_pTC10193 | AAur_pTC20040 | 966 bp | 969 | 95.34 | 72.91 | 72.91 | 2.00E-96 | integral membrane protein putative |
| AAur_pTC10194 | AAur_pTC20042 | 1863 bp | 2106 | 89.05 | 59.15 | 59.15 | 4.20E-66 | copper resistance membrane protein (CopD) putative |
| AAur_pTC10196 | AAur_0407 | 630 bp | 633 | 88.41 | 59.00 | 59.00 | 4.90E-15 | copper resistance protein CopC family putative |
| AAur_pTC10197 | AAur_pTC10144 | 750 bp | 723 | 96.13 | 79.67 | 79.67 | 6.80E-97 | ccdA cytochrome c biogenesis membrane protein |
| AAur_pTC10198 | AAur_pTC20034 | 630 bp | 597 | 91.43 | 70.60 | 70.60 | 7.10E-51 | lipoprotein putative |
| AAur_pTC10199 | AAur_pTC20035 | 585 bp | 672 | 98.97 | 67.97 | 67.97 | 3.10E-46 | integral membrane protein putative |
| AAur_pTC10215 | AAur_pTC10223 | 1884 bp | 1203 | 51.70 | 98.77 | 98.77 | 3.60E-209 | dihydrolipoamide dehydrogenase truncation |
| AAur_pTC10222 | AAur_pTC10215 | 1548 bp | 1884 | 81.40 | 86.14 | 86.14 | 2.80E-201 | degenerate dihydrolipoamide dehydrogenase/degenerate ISPps1 transposase fusion |
| AAur_pTC10223 | AAur_pTC10215 | 1203 bp | 1884 | 80.88 | 98.77 | 98.77 | 2.30E-209 | degenerate dihydrolipoamide dehydrogenase/degenerate ISPps1 transposase fusion |
| AAur_pTC10228 | AAur_1850 | 1503 bp | 1581 | 76.78 | 77.16 | 77.16 | 1.20E-199 | multicopper oxidase family protein putative |
| AAur_pTC10230 | AAur_pTC20184 | 1407 bp | 1398 | 97.51 | 73.73 | 73.73 | 7.30E-154 | metallo-beta-lactamase superfamily protein |
| AAur_pTC10231 | AAur_pTC10233 | 318 bp | 297 | -72.01 | 62.34 | 62.34 | 9.10E-06 | rhodanese-like domain protein |
| AAur_pTC10232 | AAur_3045 | 363 bp | 378 | 94.21 | 73.91 | 73.91 | 7.10E-34 | trx thioredoxin |
| AAur_pTC10242 | AAur_pTC20008 | 630 bp | 630 | 95.40 | 67.48 | 67.48 | 1.20E-44 | site-specific recombinase resolvase family |
| AAur_pTC10247 | AAur_2550 | 816 bp | 972 | 85.17 | 66.95 | 66.95 | 7.20E-50 | ABC-type nitrate/sulfonate/taurine/bicarbonate transport systems ATPase component putative |
| AAur_pTC10261 | AAur_1384 | 279 bp | 279 | 99.64 | 100.00 | 100.00 | 1.70E-56 | ISAau1 transposase orfA |
| AAur_pTC10262 | AAur_1383 | 840 bp | 840 | 99.88 | 100.00 | 100.00 | 1.10E-183 | ISAau1 transposase orfB |
| AAur_pTC10266 | AAur_2457 | 1737 bp | 2037 | 94.53 | 79.21 | 79.21 | 9.20E-224 | thiC thiamine biosynthesis |
| AAur_pTC20008 | AAur_0420 | 630 bp | 603 | 95.71 | 81.05 | 81.05 | 2.90E-82 | site-specific recombinase resolvase family |
| AAur_pTC20019 | AAur_2466 | 1047 bp | 1029 | 98.28 | 73.96 | 73.96 | 2.00E-114 | conserved hypothetical protein |
| AAur_pTC20020 | AAur_2465 | 4905 bp | 4914 | 99.98 | 73.50 | 73.50 | 0 | hypothetical protein |
| AAur_pTC20025 | AAur_pTC10142 | 879 bp | 891 | 90.79 | 64.69 | 64.69 | 1.00E-47 | BNR/Asp-box repeat domain protein |
| AAur_pTC20026 | AAur_3084 | 612 bp | 636 | 99.67 | 69.23 | 69.23 | 4.70E-48 | secreted protein with unknown function DUF305-family putative |
| AAur_pTC20028 | AAur_pTC10139 | 2142 bp | 2157 | 99.07 | 74.28 | 74.28 | 4.00E-242 | copper-translocating P-type ATPase |
| AAur_pTC20030 | AAur_pTC10189 | 387 bp | 402 | 97.42 | 76.05 | 76.05 | 4.80E-40 | transcriptional regulator ArsR family putative |
| AAur_pTC20031 | AAur_pTC10188 | 1956 bp | 1959 | 99.95 | 79.62 | 79.62 | 4.20E-268 | cadA cadmium-translocating P-type ATPase |
| AAur_pTC20032 | AAur_pTC10187 | 516 bp | 513 | 95.35 | 70.48 | 70.48 | 4.90E-44 | lspA Lipoprotein signal peptidase |
| AAur_pTC20033 | AAur_pTC10197 | 666 bp | 750 | 99.70 | 74.44 | 74.44 | 1.20E-71 | ccdA cytochrome c biogenesis membrane protein |
| AAur_pTC20034 | AAur_pTC10198 | 597 bp | 630 | 95.98 | 70.60 | 70.60 | 6.70E-51 | Thiol disulfide interchange protein thioredoxin family |
| AAur_pTC20035 | AAur_2370 | 672 bp | 879 | 97.02 | 71.92 | 71.92 | 2.40E-61 | integral membrane protein putative |
| AAur_pTC20036 | AAur_pTC10178 | 837 bp | 825 | 97.85 | 58.59 | 58.59 | 7.50E-27 | M23 peptidase domain protein |
| AAur_pTC20038 | AAur_2117 | 858 bp | 867 | 84.27 | 60.70 | 60.70 | 1.90E-35 | lipoprotein putative |
| AAur_pTC20039 | AAur_3287 | 1632 bp | 1695 | 97.00 | 72.23 | 72.23 | 1.00E-168 | cytochrome c biogenesis membrane protein ResB-like family putative |
| AAur_pTC20040 | AAur_pTC10193 | 969 bp | 966 | 95.36 | 72.91 | 72.91 | 2.00E-96 | Membrane protein putative |
| AAur_pTC20043 | AAur_pTC10190 | 378 bp | 441 | 88.36 | 73.29 | 73.29 | 3.90E-32 | transcriptional regulator ArsR family putative |
| AAur_pTC20044 | AAur_pTC10191 | 918 bp | 807 | 87.04 | 76.53 | 76.53 | 6.90E-98 | czc cobalt-zinc-cadmium efflux permease |
| AAur_pTC20045 | AAur_1365 | 693 bp | 669 | 89.61 | 73.92 | 73.92 | 3.90E-67 | DSBA-like thioredoxin domain protein putative |
| AAur_pTC20047 | AAur_pTC10196 | 663 bp | 630 | 88.84 | 57.51 | 57.51 | 1.50E-11 | Copper resistance protein CopC-family putative |
| AAur_pTC20050 | AAur_pTC10168 | 1254 bp | 1251 | 99.28 | 75.54 | 75.54 | 4.90E-147 | mntH H(+)-stimulated manganese uptake protein NRAMP-family Mn2+/Fe2+ transporters |
| AAur_pTC20054 | AAur_3635 | 489 bp | 510 | 98.57 | 69.72 | 69.72 | 3.10E-40 | phosphinothricin N-acetyltransferase putative |
| AAur_pTC20055 | AAur_3634 | 348 bp | 378 | 99.71 | 75.42 | 75.42 | 9.00E-36 | transcriptional regulator ArsR family putative |
| AAur_pTC20057 | AAur_3631 | 675 bp | 375 | 55.11 | 68.63 | 68.63 | 2.60E-30 | arsenate reductase putative |
| AAur_pTC20058 | AAur_3630 | 423 bp | 423 | 99.76 | 83.73 | 83.73 | 7.60E-61 | arsC arsenate reductase |
| AAur_pTC20059 | AAur_3629 | 990 bp | 978 | 98.08 | 81.07 | 81.07 | 9.30E-139 | trxB thioredoxin-disulfide reductase |
| AAur_pTC20060 | AAur_3628 | 1530 bp | 1380 | 89.02 | 76.06 | 76.06 | 1.80E-166 | FAD dependent oxidoreductase domain protein putative |
| AAur_pTC20067 | AAur_pTC10126 | 285 bp | 366 | 99.64 | 100.00 | 100.00 | 5.60E-58 | conserved hypothetical protein |
| AAur_pTC20068 | AAur_pTC10125 | 1266 bp | 1266 | 99.92 | 100.00 | 100.00 | 4.60E-280 | IS110 family transposase |
| AAur_pTC20069 | AAur_pTC10124 | 1341 bp | 1686 | 98.96 | 77.44 | 77.44 | 6.70E-168 | Partitioning protein parB-family putative |
| AAur_pTC20073 | AAur_4164 | 516 bp | 582 | 96.32 | 74.30 | 74.30 | 4.90E-52 | ssb single-strand binding protein |
| AAur_pTC20083 | AAur_3624 | 594 bp | 591 | 98.65 | 69.65 | 69.65 | 4.70E-51 | transcriptional regulator TetR family putative |
| AAur_pTC20087 | AAur_pTC20216 | 1455 bp | 1497 | 93.75 | 56.03 | 56.03 | 1.40E-32 | amino acid permease purative |
| AAur_pTC20088 | AAur_2032 | 1917 bp | 1935 | 99.48 | 78.71 | 78.71 | 2.20E-257 | FAD monooxygenase PheA/TfdB family putative |
| AAur_pTC20089 | AAur_3917 | 1413 bp | 1470 | 97.52 | 78.16 | 78.16 | 1.20E-175 | fumarylacetoacetate hydrolase family protein |
| AAur_pTC20090 | AAur_3916 | 681 bp | 678 | 96.77 | 80.18 | 80.18 | 1.50E-88 | transcriptional regulater GntR family |
| AAur_pTC20093 | AAur_3913 | 687 bp | 786 | 98.98 | 84.48 | 84.48 | 5.10E-104 | hpaH 2-oxo-hepta-3-ene-1 7-dioic acid hydratase |
| AAur_pTC20094 | AAur_3912 | 804 bp | 804 | 97.39 | 71.83 | 71.83 | 1.70E-79 | 2 4-dihydroxyhept-2-ene-1 7-dioic acid aldolase (HpaI) putative |
| AAur_pTC20095 | AAur_1331 | 1521 bp | 1482 | 96.38 | 74.97 | 74.97 | 1.20E-171 | succinate-semialdehyde dehydrogenase putative |
| AAur_pTC20096 | AAur_3626 | 1410 bp | 1413 | 99.93 | 77.13 | 77.13 | 1.60E-179 | maoB/feaB phenylacetaldehyde dehydrogenase |
| AAur_pTC20102 | AAur_3626 | 1410 bp | 1413 | 99.93 | 77.28 | 77.28 | 2.50E-180 | maoB/feaB phenylacetaldehyde dehydrogenase |
| AAur_pTC20107 | AAur_pTC20102 | 345 bp | 1410 | 99.42 | 99.42 | 99.42 | 4.50E-71 | Aldehyde dehydrogenase family protein putative |
| AAur_pTC20125 | AAur_3720 | 426 bp | 432 | 98.59 | 77.73 | 77.73 | 3.10E-50 | panD aspartate 1-decarboxylase |
| AAur_pTC20128 | AAur_pTC20163 | 4353 bp | 1863 | 42.61 | 78.30 | 78.30 | 1.40E-247 | conserved hypothetical protein |
| AAur_pTC20130 | AAur_1141 | 930 bp | 963 | 93.23 | 61.67 | 61.67 | 7.60E-46 | conserved hypothetical protein |
| AAur_pTC20131 | AAur_1140 | 1128 bp | 1107 | 92.73 | 65.60 | 65.60 | 7.50E-76 | oxidoreductase family NAD-binding Rossmann fold domain protein |
| AAur_pTC20132 | AAur_3311 | 1281 bp | 1371 | 96.49 | 60.61 | 60.61 | 5.90E-60 | extracellular solute-binding domain protein |
| AAur_pTC20133 | AAur_3312 | 933 bp | 942 | 94.21 | 73.57 | 73.57 | 1.20E-94 | ABC-type sugar transport system permease component putative |
| AAur_pTC20137 | AAur_pTC20225 | 567 bp | 567 | 99.82 | 94.53 | 94.53 | 3.00E-109 | transposon resolvase |
| AAur_pTC20149 | AAur_2928 | 579 bp | 579 | 94.47 | 79.05 | 79.05 | 1.60E-70 | adk Adenylate kinase (ATP-AMP transphosphorylase) |
| AAur_pTC20150 | AAur_1660 | 1143 bp | 1140 | 97.55 | 80.98 | 80.98 | 2.70E-158 | gltA citrate synthase |
| AAur_pTC20151 | AAur_1661 | 906 bp | 906 | 99.89 | 81.92 | 81.92 | 1.20E-131 | prpB methylisocitrate lyase |
| AAur_pTC20152 | AAur_1662 | 1521 bp | 1521 | 99.93 | 83.32 | 83.32 | 7.60E-234 | 2-methylcitrate dehydratase putative |
| AAur_pTC20153 | AAur_1663 | 657 bp | 642 | 97.56 | 77.73 | 77.73 | 1.00E-80 | transcriptional regulator GntR family |
| AAur_pTC20163 | AAur_pTC20128 | 1863 bp | 4353 | 99.95 | 78.30 | 78.30 | 6.10E-248 | hypothetical protein |
| AAur_pTC20165 | AAur_pTC10132 | 2313 bp | 2322 | 99.96 | 78.92 | 78.92 | 4.6e-314 | copper-translocating P-type ATPase |
| AAur_pTC20173 | AAur_2094 | 282 bp | 1557 | 99.65 | 85.87 | 85.87 | 2.40E-41 | zwf glucose-6-phosphate 1-dehydrogenase |
| AAur_pTC20174 | AAur_2093 | 1094 bp | 942 | 84.10 | 78.34 | 78.34 | 2.30E-118 | opcA protein putative |
| AAur_pTC20184 | AAur_pTC10230 | 1398 bp | 1407 | 98.14 | 73.73 | 73.73 | 7.20E-154 | Metallo-beta-lactamase superfamily protein |
| AAur_pTC20188 | AAur_0495 | 402 bp | 2154 | 71.89 | 75.77 | 75.77 | 8.00E-30 | conserved hypothetical protein |
| AAur_pTC20192 | AAur_1378 | 1935 bp | 1941 | 99.79 | 80.03 | 80.03 | 6.70E-271 | amine oxidase (copper-containing) |
| AAur_pTC20193 | AAur_1383 | 840 bp | 840 | 99.88 | 100.00 | 100.00 | 1.10E-183 | ISAau1 transposase orfB |
| AAur_pTC20194 | AAur_1384 | 306 bp | 279 | 90.85 | 100.00 | 100.00 | 1.70E-56 | ISAau1 transposase orfA |
| AAur_pTC20195 | AAur_1379 | 525 bp | 432 | 78.48 | 65.88 | 65.88 | 7.60E-26 | protein of unknown function (DUF779) putative |
| AAur_pTC20196 | AAur_3064 | 1524 bp | 1524 | 99.93 | 83.75 | 83.75 | 5.80E-237 | aldehyde dehydrogenase (NAD) family protein |
| AAur_pTC20199 | AAur_pTC20112 | 213 bp | 654 | 87.32 | 72.34 | 72.34 | 4.20E-14 | Isochorismatase family protein putative |
| AAur_pTC20204 | AAur_3870 | 1116 bp | 1119 | 99.91 | 80.30 | 80.30 | 3.30E-155 | ald alanine dehydrogenase |
| AAur_pTC20206 | AAur_pTC10062 | 765 bp | 732 | 89.93 | 61.74 | 61.74 | 5.40E-32 | glutamine amidotransferase putative |
| AAur_pTC20207 | AAur_pTC10071 | 1785 bp | 1905 | 55.91 | 57.84 | 57.84 | 2.10E-27 | GAF domain protein |
| AAur_pTC20208 | AAur_pTC10068 | 1107 bp | 1167 | 98.10 | 58.16 | 58.16 | 3.40E-33 | ferredoxin reductase putative |
| AAur_pTC20210 | AAur_pTC10066 | 1194 bp | 1164 | 82.58 | 63.54 | 63.54 | 6.60E-56 | Cytochrome P450 putative |
| AAur_pTC20211 | AAur_pTC10065 | 741 bp | 726 | 92.98 | 64.76 | 64.76 | 2.70E-41 | glutamine amidotransferase putative |
| AAur_pTC20212 | AAur_pTC10064 | 774 bp | 774 | 99.87 | 69.64 | 69.64 | 7.90E-69 | oxidoreductase short chain dehydrogenase/reductase family |
| AAur_pTC20213 | AAur_pTC10069 | 1392 bp | 1419 | 85.06 | 69.26 | 69.26 | 8.30E-107 | ipuC gamma-glutamylisopropylamide synthetase |
| AAur_pTC20215 | AAur_pTC10063 | 1368 bp | 1353 | 99.27 | 66.79 | 66.79 | 1.30E-105 | Aldehyde dehydrogenase (NAD) family protein |
| AAur_pTC20218 | AAur_pTC10058 | 1002 bp | 1116 | 97.41 | 67.81 | 67.81 | 2.80E-79 | alcohol dehydrogenase putative |
| AAur_pTC20219 | AAur_pTC10061 | 1497 bp | 1473 | 95.66 | 60.50 | 60.50 | 1.10E-65 | maoB/feaB phenylacetaldehyde dehydrogenase |
| AAur_pTC20220 | AAur_3553 | 1272 bp | 1218 | 89.47 | 62.18 | 62.18 | 2.00E-57 | Metal-dependent amidase/aminoacylase/carboxypeptidase putative |
| AAur_pTC20221 | AAur_pTC20225 | 409 bp | 567 | 69.44 | 85.96 | 85.96 | 1.20E-41 | transposon resolvase |
| AAur_pTC20225 | AAur_pTC20137 | 567 bp | 567 | 99.82 | 94.53 | 94.53 | 3.00E-109 | transposon resolvase authentic point mutation |
| AAur_pTC20226 | AAur_1074 | 780 bp | 744 | 48.97 | 63.82 | 63.82 | 1.30E-45 | two-component system response regulator |
| AAur_pTC20227 | AAur_2852 | 801 bp | 933 | 97.25 | 72.17 | 72.17 | 6.20E-79 | galU UTP-glucose-1-phosphate uridylyltransferase |
| AAur_pTC20242 | AAur_1145 | 744 bp | 909 | 95.30 | 62.60 | 62.60 | 5.40E-40 | ANTAR domain protein |
| AAur_pTC20243 | AAur_0316 | 570 bp | 573 | 44.39 | 62.02 | 62.02 | 4.50E-10 | transcriptional regulator MarR family putative |
| AAur_pTC20271 | AAur_pTC10105 | 1077 bp | 585 | 53.11 | 77.78 | 77.78 | 2.00E-69 | Conserved hypothetical protein |
| AAur_pTC20273 | AAur_pTC10095 | 1878 bp | 1926 | 85.25 | 55.96 | 55.96 | 9.40E-29 | Hypothetical protein |
