# Supplementary material for: Secrets of Soil Survival Revealed by the Genome Sequence of Arthrobacter aurescens TC1
Source: PLoS Genet. 2006 Dec 22;2(12):e214. doi: 10.1371/journal.pgen.0020214 (PMC1713258; doi:10.1371/journal.pgen.0020214)
Supplement: Supplementary file 5 [file pgen.0020214.st002.doc]

**Supplemental Table 2.** *Arthrobacter aurescens* chromosomally-encoded genes with atypical composition.

| **ORF** | **End5** | **End3** | **Predicted Protein Product** | **Organism of Top BLASTP hit** | **Protein accession of Top BLASTP hit** |
| --- | --- | --- | --- | --- | --- |
| **Region 1: position 145000 - 169000 , Mean Chi^2^: 683.2 , Average GC: 56.06** | | | | |  |
| AAur_0145 | 144825 | 146396 | putative bacterial membrane flanked domain protein | *Leifsonia xyli* subsp. xyli str. CTCB07 | YP_062942.1 |
| AAur_0146 | 146389 | 147297 | putative NADP oxidoreductase coenzyme F420-dependent family protein | *Streptomyces coelicolor* A3(2) | NP_627591.1 |
| AAur_0147 | 147303 | 148205 | pantoate--beta-alanine ligase | *Corynebacterium efficiens* YS-314 | NP_736725.1 |
| AAur_0148 | 148311 | 149828 | lincomycin resistance protein | *Corynebacterium efficiens* YS-314 | NP_739141.1 |
| AAur_0149 | 150038 | 149916 | hypothetical protein | -- | -- |
| AAur_0153 | 156696 | 156151 | hypothetical protein | -- | -- |
| AAur_0154 | 157326 | 156742 | putative HhH-GPD superfamily base excision DNA repair protein | *Mycobacterium avium* subsp. paratuberculosis K-10 | NP_961562.1 |
| AAur_0158 | 164294 | 163440 | putative transcriptional regulator, LysR family | *Pseudomonas aeruginosa* PAO1 | NP_250261.1 |
| AAur_0159 | 164321 | 165289 | Type I phosphodiesterase / nucleotide pyrophosphatase family protein | *Rhodopirellula baltica* SH 1 | NP_868571.1 |
| AAur_0160 | 165286 | 166143 | putative Integral membrane protein DUF6 | *Streptomyces avermitilis* MA-4680 | NP_824790.1 |
| AAur_0161 | 167001 | 166168 | putative hydrolase, alpha/beta fold family protein | *Mesorhizobium loti* MAFF303099 | NP_103647.1 |
| AAur_0162 | 167919 | 167065 | putative hydrolase | *Mesorhizobium loti* MAFF303099 | NP_103647.1 |
| AAur_0163 | 168825 | 167929 | conserved hypothetical protein | *Mesorhizobium loti* MAFF303099 | NP_103647.1 |
| AAur_0164 | 168901 | 170430 | lysyl-tRNA synthetase | *Propionibacterium acnes* KPA171202 | YP_054897.1 |
| **Region 2: position 959000 - 974000, Mean Chi^2^: 674.4, Average GC: 56.22** | | | |  |  |
| AAur_0910 | 960134 | 962344 | 1,4-alpha-glucan branching enzyme | *Leifsonia xyli* subsp. xyli | Q6AEU4 |
| AAur_0915 | 968985 | 969464 | acetyltransferase, GNAT family protein | *Streptomyces avermitilis* MA-4680 | NP_825281.1 |
| AAur_0916 | 970494 | 969472 | putative transcriptional regulator, lacI family | *Frankia* sp. CcI3 | YP_481263.1 |
| AAur_0917 | 972368 | 970515 | alpha-amylase family protein | *Thermobifida fusca* YX | YP_288894.1 |
| AAur_0918 | 972471 | 973769 | maltose-binding protein | *Propionibacterium acnes* KPA171202 | YP_056254.1 |
| AAur_0919 | 973867 | 975525 | maltose ABC transporter, permease protein | *Propionibacterium acnes* KPA171202 | YP_056255.1 |
| **Region 3: position 1232000 – 1271000, Mean Chi^2^: 903, Average GC: 53.17** | | | |  |  |
| AAur_1158 | 1233558 | 1231912 | putative RNA polymerase sigma (70) factor | *Streptomyces avermitilis* MA-4680 | NP_827253.1 |
| AAur_1159 | 1234186 | 1233683 | putative transcriptional regulator, MarR family | *Nonomuraea recticatena* | BAE78753.1 |
| AAur_1160 | 1234274 | 1235200 | putative transmembrane protein | *Shewanella denitrificans* OS217 | YP_562596.1 |
| AAur_1164 | 1240447 | 1237619 | conserved domain protein | *Azoarcus sp*. EbN1 | YP_160657.1 |
| AAur_1165 | 1242338 | 1240431 | putative protein of unknown function DUF262 family | *Acidobacteria bacterium* Ellin345 | YP_593354.1 |
| AAur_1163 | 1237604 | 1236528 | hypothetical protein | -- | -- |
| AAur_1162 | 1236227 | 1235937 | hypothetical protein | -- | -- |
| AAur_1161 | 1235838 | 1235434 | hypothetical protein | -- | -- |
| AAur_1166 | 1244453 | 1244551 | hypothetical protein | -- | -- |
| AAur_1167 | 1244627 | 1246018 | putative amino acid permease | *Pseudomonas aeruginosa* PAO1 | NP_249013.1 |
| AAur_1168 | 1246684 | 1246049 | putative oxidoreductase, short chain dehydrogenase/reductase family protein | *Rhodopseudomonas palustris* HaA2 | YP_486795.1 |
| AAur_1169 | 1247146 | 1246772 | conserved hypothetical protein | *Methanococcoides burtonii* DSM 6242 | YP_565818.1 |
| AAur_1170 | 1248436 | 1247192 | putative flavin-binding monooxygenase-like | *Synechococcus elongatus* PCC 6301 | YP_171409.1 |
| AAur_1171 | 1249751 | 1249104 | hypothetical protein | -- | -- |
| AAur_1172 | 1250056 | 1249712 | hypothetical protein | -- | -- |
| AAur_1173 | 1250112 | 1250495 | hypothetical protein | -- | -- |
| AAur_1174 | 1250886 | 1251089 | putative cold shock protein | *Streptomyces avermitilis* MA-4680 | NP_825997.1 |
| AAur_1175 | 1252108 | 1251167 | putative integral membrane protein | *Bacillus licheniformis* ATCC 14580 | YP_092108.1 |
| AAur_1176 | 1252727 | 1252101 | putative ABC transporter, ATP-binding protein | *Bacillus licheniformis* ATCC 14580 | YP_092109.1 |
| AAur_1178 | 1253887 | 1256490 | putative transcriptional regulator, LuxR family | *Leifsonia xyli* subsp. xyli str. CTCB07 | YP_062708.1 |
| AAur_1180 | 1256512 | 1257765 | putative amino acid permease | *Mycobacterium bovis* | P63350 |
| AAur_1179 | 1259048 | 1257762 | putative transmembrane efflux protein (MFS) | *Erwinia carotovora* subsp. atroseptica SCRI1043 | YP_049652.1 |
| AAur_1181 | 1259979 | 1259413 | putative Alpha/Beta hydrolase family of unknown function (DUF1234) | *Burkholderia* sp. 383 | YP_373263.1 |
| AAur_1182 | 1261415 | 1260030 | UDP-glucose 6-dehydrogenase | *Leifsonia xyli* subsp. xyli str. CTCB07 | YP_061472.1 |
| AAur_1183 | 1262581 | 1261412 | putative glycosyl transferase, group 1 family protein | *Propionibacterium* acnes KPA171202 | YP_056479.1 |
| AAur_1185 | 1265217 | 1263883 | hypothetical protein | -- | -- |
| AAur_1186 | 1265714 | 1265322 | putative transcriptional regulator, MerR family | *Mycobacterium avium* subsp. paratuberculosis K-10 | NP_962201.1 |
| AAur_1187 | 1265868 | 1266290 | putative heat shock protein | *Streptomyces avermitilis* MA-4680 | NP_821867.1 |
| AAur_1188 | 1266148 | 1266900 | putative DnaJ domain protein | *Mycobacterium avium* subsp. paratuberculosis K-10 | NP_960629.1 |
| AAur_1189 | 1266897 | 1267274 | hypothetical protein | -- | -- |
| AAur_1190 | 1267422 | 1268459 | glyoxalase family protein | *Streptomyces lividans* | AAB38012.1 |
| AAur_1191 | 1268934 | 1270265 | putative integral membrane protein | *Thermobifida fusca* YX | YP_290631.1 |
| AAur_1192 | 1270769 | 1270320 | hypothetical protein | -- | -- |
| AAur_1193 | 1270781 | 1271350 | phosphoribosylglycinamide formyltransferase | *Streptomyces avermitilis* MA-4680 | NP_824622.1 |
| **Region 4: position 1465000 – 1500000, Mean Chi^2^: 532, Average GC: 69.5** | | | |  |  |
| AAur_1366 | 1465136 | 1464126 | putative transcription regulator, LacI family | *Streptomyces avermitilis* MA-4680 | NP_828332.1 |
| AAur_1368 | 1465280 | 1466746 | putative sugar MFS transporter | *Corynebacterium glutamicum* ATCC 13032 | YP_224478.1 |
| AAur_1369 | 1468457 | 1471594 | hypothetical protein | *Streptomyces coelicolor* A3(2) | NP_639883.1 |
| AAur_1370 | 1471594 | 1472700 | Tn554-related transposase A | *Mycobacterium gordonae* | AAB54012.1 |
| AAur_1371 | 1472697 | 1474931 | Tn554-related transposase B | *Arthrobacter nicotinovorans* | CAD47908.1 |
| AAur_1372 | 1474921 | 1475436 | Tn554-related transposase C | *Arthrobacter nicotinovorans* | CAD47909.1 |
| AAur_1373 | 1475495 | 1475671 | IS256 family transposase, truncation | *Mycobacterium ulcerans* Agy99 | YP_025583.1 |
| AAur_1374 | 1476151 | 1475727 | adenylate kinase, putative, authentic FRAMESHIFT | *Micrococcus luteus* | P33107 |
| AAur_1375 | 1478570 | 1476558 | acetate--CoA ligase | *Streptomyces avermitilis* | Q82EL5 |
| AAur_1376 | 1479208 | 1478651 | hypothetical protein | -- | -- |
| AAur_1377 | 1479761 | 1479528 | putative membrane protein | *Streptomyces avermitilis* MA-4680 | NP_825720.1 |
| AAur_1378 | 1481885 | 1479945 | amine oxidase (copper-containing) | *Arthrobacter* sp. P1 | Q07123 |
| AAur_1379 | 1482412 | 1481981 | putative protein of unknown function (DUF779) | *Streptomyces avermitilis* MA-4680 | NP_828321.1 |
| AAur_1380 | 1484068 | 1482545 | aldehyde dehydrogenase (NAD) family protein | *Rhodococcus erythropolis* | AAZ14956.1 |
| AAur_1381 | 1485635 | 1484352 | putative major facilitator superfamily (MFS) transporter | *Archaeoglobus fulgidus* DSM 4304 | NP_069740.1 |
| AAur_1382 | 1486284 | 1485871 | Tyramine oxidase, interruption-C | *Aspergillus oryzae* | BAE64779.1 |
| AAur_1383 | 1487235 | 1486396 | ISAau1, transposase orfB | *Arthrobacter aurescens* | AAS20166.1 |
| AAur_1384 | 1487543 | 1487265 | ISAau1, transposase orfA | *Arthrobacter aurescens* | AAS20165.1 |
| AAur_1385 | 1488472 | 1487594 | Tyramine oxidase, interruption-N | *Aspergillus fumigatus* Af293 | XP_747059.1 |
| AAur_1386 | 1489151 | 1488735 | putative protein of unknown function (DUF861) | -- | -- |
| AAur_1387 | 1489635 | 1490690 | IS110 family transposase, degenerate | *Streptomyces coelicolor* | P14707 |
| AAur_1388 | 1491636 | 1491028 | putative transcriptional regulator, TetR family | *Mycobacterium smegmatis* str. MC2 155 | GMSORF2050 |
| AAur_1389 | 1491810 | 1494881 | Tn3 family transposase | *Streptomyces tenjimariensis* | CAI59998.1 |
| AAur_1390 | 1494906 | 1495028 | hypothetical protein | -- | -- |
| AAur_1391 | 1495025 | 1495429 | putative glyoxalase family protein | *Leifsonia xyli* subsp. xyli str. CTCB07 | YP_062364.1 |
| AAur_1392 | 1498152 | 1496050 | phage integrase family domain protein | *Nocardia farcinica* IFM 10152 | YP_121978.1 |
| AAur_1393 | 1499939 | 1498149 | hypothetical protein | *Nocardia farcinica* IFM 10152 | YP_121979.1 |
| AAur_1394 | 1501507 | 1499942 | phage integrase family domain protein | *Nocardia farcinica* IFM 10152 | YP_121980.1 |
| **Region 5: position 1791000 – 1807000, Mean Chi^2^: 683.16, Average GC: 56.25** | | | |  |  |
| AAur_1641 | 1790665 | 1791291 | putative 3-methyladenine DNA glycosylase | *Leifsonia xyli* subsp. xyli str. CTCB07 | YP_061674.1 |
| AAur_1642 | 1791423 | 1793651 | putative DNA or RNA helicase, Superfamily I | *Leifsonia xyli* subsp. xyli str. CTCB07 | YP_062882.1 |
| AAur_1643 | 1793689 | 1794999 | tyrosyl-tRNA synthetase | *Leifsonia xyli* subsp. xyli str. CTCB07 | YP_061678.1 |
| AAur_1644 | 1795262 | 1795038 | hypothetical protein | -- | -- |
| AAur_1645 | 1795451 | 1795323 | hypothetical protein | -- | -- |
| AAur_1649 | 1801796 | 1803892 | conserved hypothetical protein | *Leifsonia xyli* subsp. xyli str. CTCB07 | YP_061320.1 |
| AAur_1650 | 1803885 | 1804871 | putative haloacid dehalogenase-like hydrolase | *Leifsonia xyli* subsp. xyli str. CTCB07 | YP_061321.1 |
| AAur_1651 | 1804881 | 1805141 | conserved hypothetical protein | -- | -- |
| AAur_1652 | 1805145 | 1805957 | hemolysin A | *Streptomyces coelicolor* A3(2) | NP_626053.1 |
| AAur_1653 | 1805971 | 1807011 | ATP-NAD kinase | *Micrococcus luteus* | BAB84189.1 |
| **Region 6: position 3386000 – 3398000, Mean Chi^2^: 656, Average GC: 68.6** | | | |  |  |
| AAur_3102 | 3386585 | 3385824 | ubiquinone/menaquinone biosynthesis methyltransferase | *Streptomyces avermitilis* | Q81ZX2 |
| AAur_3103 | 3386677 | 3388032 | isochorismate synthase | *Thermobifida fusca* YX | YP_289474.1 |
| AAur_3104 | 3388191 | 3388988 | putative amino acid ABC transporter | *Streptomyces avermitilis* MA-4680 | NP_827576.1 |
| AAur_3105 | 3389093 | 3389884 | putative amino acid ABC transporter, permease protein | *Streptomyces avermitilis* MA-4680 | NP_827577.1 |
| AAur_3106 | 3389896 | 3390690 | glutamine ABC transporter, ATP-binding protein | *Streptomyces avermitilis* MA-4680 | NP_827578.1 |
| AAur_3108 | 3390901 | 3391728 | hypothetical protein | *Mycoplasma pulmonis* UAB CTIP | NP_326356.1 |
| AAur_3107 | 3393424 | 3391715 | 2-succinyl-6-hydroxy-2,4-cyclohexadiene-1-carboxylic acid synthase/2-oxoglutarate decarboxylase | *Propionibacterium acnes* KPA171202 | YP_055614.1 |
| AAur_3109 | 3394061 | 3393672 | conserved hypothetical protein | *Chlorobium tepidum* TLS | NP_662010.1 |
| AAur_3110 | 3395009 | 3394170 | ISAau1, transposase orfB | *Arthrobacter aurescens* | AAS20166.1 |
| AAur_3111 | 3395317 | 3395039 | ISAau1, transposase orfA | *Arthrobacter aurescens* | AAS20165.1 |
| AAur_3112 | 3395599 | 3395877 | ISAau1, transposase orfA | *Arthrobacter aurescens* | AAS20165.1 |
| AAur_3113 | 3395907 | 3396746 | ISAau1, transposase orfB | *Arthrobacter aurescens* | AAS20166.1 |
| AAur_3114 | 3399122 | 3397035 | putative bacterial protein of unknown function (DUF839) | *Streptomyces coelicolor* A3(2) | NP_627980.1 |
| **Region 7: position 3412000 – 3423000, Mean Chi^2^: 506, Average GC: 63.6** | | | |  |  |
| AAur_3126 | 3414225 | 3411406 | putative serine protease, subtilase family | *Stenotrophomonas maltophilia* | AAP13815.1 |
| AAur_3127 | 3415590 | 3414373 | putative major facilitator superfamily (MFS) transporter | *Bordetella pertussis* Tohama I | NP_878946.1 |
| AAur_3128 | 3418523 | 3416391 | putative LPXTG-motif cell wall anchor domain protein | *Anopheles gambiae* str. PEST | XP_559447.1 |
| AAur_3129 | 3419707 | 3418622 | conserved hypothetical protein | *Methanospirillum hungatei* JF-1 | YP_502900.1 |
| AAur_3130 | 3420510 | 3419821 | conserved hypothetical protein | *Streptomyces avermitilis* MA-4680 | NP_826745.1 |
| AAur_3131 | 3422483 | 3420786 | putative polysaccharide pyruvyl transferase family protein | *Thiobacillus denitrificans* ATCC 25259 | YP_315234.1 |
| AAur_3132 | 3425364 | 3422419 | putative S-layer domain protein | *Streptomyces avermitilis* MA-4680 | NP_823888.1 |
| **Region 8: position 3459000 – 3479000, Mean Chi^2^: 649, Average GC: 54.5** | | | |  |  |
| AAur_3158 | 3458799 | 3459347 | putative uncharacterized DUF218 domain protein | *Corynebacterium glutamicum* ATCC 13032 | YP_227253.1 |
| AAur_3159 | 3460640 | 3459744 | glucose-1-phosphate thymidylyltransferase | *Nocardia farcinica* IFM 10152 | YP_116413.1 |
| AAur_3160 | 3460627 | 3461694 | dTDP-glucose 4,6-dehydratase | *Enterococcus faecalis* V583 | NP_815854.1 |
| AAur_3161 | 3461696 | 3463129 | dTDP-4-keto-L-rhamnose reductase and dTDP-4-keto-6-deoxyglucose-3,5-epimerase | *Corynebacterium glutamicum* ATCC 13032 | YP_224633.1 |
| AAur_3162 | 3465524 | 3464010 | putative cell surface polysaccharide biosynthesis | *Corynebacterium glutamicum* ATCC 13032 | YP_224644.1 |
| AAur_3163 | 3467827 | 3466805 | hypothetical protein | -- | -- |
| AAur_3165 | 3469840 | 3468614 | putative integral membrane protein | -- | -- |
| AAur_3166 | 3471136 | 3469850 | putative integral membrane protein | *Citrobacter freundii* | AAV34496.1 |
| AAur_3167 | 3473128 | 3472031 | hypothetical protein | *Bacteroides fragilis* NCTC 9343 | YP_211682.1 |
| AAur_3168 | 3474266 | 3473112 | putative glycosyl transferase, group 1 family protein | *Desulfovibrio vulgaris* subsp. vulgaris str. Hildenborough | YP_009504.1 |
| AAur_3169 | 3474844 | 3476208 | putative glucosyltransferase | *Frankia* sp. CcI3 | YP_480444.1 |
| AAur_3170 | 3476261 | 3477574 | UDP-glucose 6-dehydrogenase | *Streptomyces coelicolor* A3(2) | NP_627273.1 |
| AAur_3171 | 3477578 | 3479443 | hypothetical protein | *Frankia* sp. CcI3 | YP_482553.1 |
| **Region 9: position 3850000 - end3: 3862000, Mean Chi^2^: 852.5, Average GC: 52.5** | | | |  |  |
| AAur_3501 | 3850506 | 3849199 | extracellular solute-binding domain protein | *Pyrococcus furiosus* DSM 3638 | NP_579468.1 |
| AAur_3502 | 3851403 | 3850705 | conserved hypothetical protein | *Bacillus anthracis* str. 'Ames Ancestor' | YP_019711.1 |
| AAur_3503 | 3852491 | 3851493 | putative transcriptional regulator, lacI family | *Thermoanaerobacter tengcongensis* MB4 | NP_623527.1 |
| AAur_3504 | 3853484 | 3854014 | hypothetical protein | -- | -- |
| AAur_3505 | 3856584 | 3855148 | hypothetical protein | -- | -- |
| AAur_3506 | 3857018 | 3857821 | putative hydroxypyruvate isomerase | *Streptomyces coelicolor* | Q9Z596 |
| AAur_3507 | 3857911 | 3858789 | 2-hydroxy-3-oxopropionate reductase | *Streptomyces avermitilis* MA-4680 | NP_823201.1 |
| AAur_3508 | 3858915 | 3860690 | glyoxylate carboligase | *Burkholderia xenovorans* LB400 | YP_558565.1 |
| AAur_3509 | 3860716 | 3861882 | glycerate kinase | *Streptomyces coelicolor* A3(2) | NP_630549.1 |
| AAur_3510 | 3861875 | 3863224 | allantoinase | *Streptomyces coelicolor* | Q9RKU5 |
| **Region 10: position 4103000 – 4127000, Mean Chi^2^: 718.6, Average GC: 56.04** | | | |  |  |
| AAur_3742 | 4104793 | 4102538 | putative copper resistance protein D family | *Leifsonia xyli* subsp. xyli str. CTCB07 | YP_063209.1 |
| AAur_3743 | 4105211 | 4104924 | DNA-binding protein HU | *Leifsonia xyli* subsp. xyli str. CTCB07 | YP_063210.1 |
| AAur_3744 | 4106004 | 4105441 | putative ribosomal-protein-alanine acetyltransferase | *Clostridium perfringens* str. 13 | NP_560975.1 |
| AAur_3745 | 4106208 | 4107179 | conserved hypothetical protein | *Thermobifida fusca* YX | YP_288530.1 |
| AAur_3749 | 4114104 | 4113223 | putative epoxide hydrolase | *Streptomyces coelicolor* A3(2) | NP_625198.1 |
| AAur_3750 | 4114226 | 4116148 | putative afsR/dnrI/redD regulatory family protein | *Streptomyces coelicolor* A3(2) | NP_625197.1 |
| AAur_3754 | 4123060 | 4122755 | ribosomal protein S14p/S29e | *Leifsonia xyli* subsp. xyli | Q6ABY6 |
| AAur_3755 | 4123232 | 4123065 | ribosomal protein L33 | *Leifsonia xyli* subsp. xyli | Q6ABY5 |
| AAur_3756 | 4123468 | 4123232 | ribosomal protein L28 | *Leifsonia xyli* subsp. xyli | Q6ABY4 |
| AAur_3757 | 4124625 | 4123861 | GDSL-like Lipase/Acylhydrolase domain protein | *Leifsonia xyli* subsp. xyli str. CTCB07 | YP_062143.1 |
| AAur_3758 | 4125158 | 4124679 | putative transcriptional regulator, MarR family | *Corynebacterium efficiens* YS-314 | NP_739015.1 |
| AAur_3760 | 4125227 | 4125514 | putative YCII-related domain protein | *Chromobacterium violaceum* ATCC 12472 | NP_903820.1 |
| AAur_3759 | 4126308 | 4125490 | GDSL-like Lipase/Acylhydrolase domain protein | *Leifsonia xyli* subsp. xyli str. CTCB07 | YP_062143.1 |
| AAur_3761 | 4127141 | 4126305 | GDSL-like Lipase/Acylhydrolase domain protein | *Leifsonia xyli* subsp. xyli str. CTCB07 | YP_062143.1 |
| **Region 11: position 4520000 - 4532000, Mean Chi^2^: 524, Average GC: 54.95** | | | |  |  |
| AAur_4137 | 4519897 | 4520280 | hypothetical protein | *Nocardia farcinica* IFM 10152 | YP_119929.1 |
| AAur_4138 | 4521261 | 4520539 | putative lipoprotein | -- | -- |
| AAur_4139 | 4522749 | 4521361 | replicative DNA helicase | *Streptomyces avermitilis* MA-4680 | NP_825461.1 |
| AAur_4140 | 4523336 | 4523815 | hypothetical protein | -- | -- |
| AAur_4141 | 4523823 | 4524398 | hypothetical protein | -- | -- |
| AAur_4142 | 4526431 | 4524581 | putative Tat pathway signal sequence domain protein | -- | -- |
| AAur_4146 | 4529826 | 4531337 | putative gluconate transporter | *Bacillus cereus* E33L | YP_081772.1 |
| AAur_4143 | 4527514 | 4527146 | conserved hypothetical protein | *Thiomicrospira denitrificans* ATCC 33889 | YP_393880.1 |
| AAur_4144 | 4528178 | 4527552 | putative NADH-dependent FMN reductase | *Thermobifida fusca* YX | YP_289987.1 |
| AAur_4145 | 4529666 | 4528590 | putative Lipase/esterase protein | *Acidithiobacillus ferrooxidans* ATCC 23270 | AFE_2911 |
| AAur_4147 | 4531381 | 4532631 | putative acyl-CoA transferase, CAIB/BAIF family | *Burkholderia xenovorans* LB400 | YP_558286.1 |
