# Supplementary material for: Secrets of Soil Survival Revealed by the Genome Sequence of Arthrobacter aurescens TC1
Source: PLoS Genet. 2006 Dec 22;2(12):e214. doi: 10.1371/journal.pgen.0020214 (PMC1713258; doi:10.1371/journal.pgen.0020214)
Supplement: Supplementary file 6 [file pgen.0020214.st003.doc]

**Supplemental Table 3.** Genes on *Arthrobacter aurescens* strain TC1 plasmids pTC1 and pTC2 matching chromosomal genes by BLASTP searches.

| Plasmid ORF | Length (bp) | Chromosomal Match | Length (bp) | % identity | % length match / total length | Functional Name |
| --- | --- | --- | --- | --- | --- | --- |
| **pTC1** | | | | | | |
| AAur_pTC10098 | 150 | AAur_0076 | 149 | 93.24 | 100.00 | conserved hypothetical protein |
| AAur_pTC10099 | 149 | AAur_0075 | 148 | 89.86 | 100.00 | conserved hypothetical protein |
| AAur_pTC10101 | 106 | AAur_0074 | 285 | 80.46 | 101.19 | putative membrane protein |
| AAur_pTC10262 | 279 | AAur_1383 | 279 | 100.00 | 100.00 | ISAau1, transposase orfB |
| AAur_pTC10261 | 92 | AAur_1384 | 92 | 100.00 | 100.00 | ISAau1, transposase orfA |
| AAur_pTC10103 | 201 | AAur_0074 | 285 | 85.07 | 100.00 | putative membrane protein |
| AAur_pTC10104 | 381 | AAur_0073 | 373 | 82.04 | 100.00 | conserved hypothetical protein |
| AAur_pTC10126 | 121 | AAur_3237 | 127 | 57.94 | 100.00 | hypothetical protein |
| AAur_pTC10131 | 331 | AAur_4094 | 77 | 40.00 | 95.65 | heavy metal-associated domain protein |
| AAur_pTC10132 | 773 | AAur_4095 | 809 | 57.19 | 99.69 | copper-translocating P-type ATPase |
| AAur_pTC10134 | 109 | AAur_4093 | 121 | 49.52 | 95.19 | protein of unknown function, putative |
| AAur_pTC10138 | 111 | AAur_4094 | 77 | 61.67 | 100.00 | heavy metal-associated domain protein |
| AAur_pTC10141 | 199 | AAur_3084 | 211 | 52.00 | 100.51 | secreted protein with unknown function, DUF305-family, putative |
| AAur_pTC10143 | 239 | AAur_2075 | 170 | 43.64 | 100.00 | acetyltransferase, GNAT family protein |
| AAur_pTC10144 | 240 | AAur_3288 | 251 | 50.00 | 98.70 | cytochrome c biogenesis membrane protein |
| AAur_pTC10145 | 173 | AAur_3289 | 201 | 58.43 | 99.39 | Tat (twin-arginine translocation) domain protein, putative |
| AAur_pTC10147 | 447 | AAur_1583 | 411 | 49.61 | 98.44 | integral membrane protein, putative |
| AAur_pTC10149 | 358 | AAur_4070 | 268 | 35.23 | 108.20 | LysM domain protein, putative |
| AAur_pTC10151 | 100 | AAur_0657 | 99 | 74.00 | 101.02 | conserved hypothetical protein |
| AAur_pTC10162 | 405 | AAur_1583 | 411 | 37.69 | 96.10 | integral membrane protein, putative |
| AAur_pTC10173 | 337 | AAur_3286 | 355 | 61.54 | 99.67 | cytochrome C assembly protein, putative |
| AAur_pTC10174 | 541 | AAur_3287 | 564 | 61.46 | 99.81 | cytochrome c biogenesis membrane protein, ResB-like family, putative |
| AAur_pTC10178 | 274 | AAur_1018 | 278 | 41.40 | 98.16 | M23 peptidase domain protein |
| AAur_pTC10179 | 215 | AAur_2207 | 224 | 80.50 | 100.00 | Cytochrome c oxidase subunit III |
| AAur_pTC10181 | 174 | AAur_1781 | 156 | 54.73 | 101.38 | conserved hypothetical protein |
| AAur_pTC10184 | 105 | AAur_1288 | 127 | 50.65 | 98.68 | conserved hypothetical protein |
| AAur_pTC10190 | 146 | AAur_1363 | 108 | 54.63 | 100.00 | transcriptional regulator, ArsR family |
| AAur_pTC10191 | 268 | AAur_0412 | 306 | 48.13 | 100.76 | cobalt-zinc-cadmium efflux permease |
| AAur_pTC10192 | 218 | AAur_1365 | 222 | 57.48 | 98.58 | DSBA-like thioredoxin domain protein, putative |
| AAur_pTC10194 | 620 | AAur_3742 | 751 | 41.19 | 96.15 | copper resistance protein D family, putative |
| AAur_pTC10195 | 249 | AAur_0784 | 206 | 35.38 | 101.61 | hypothetical protein |
| AAur_pTC10196 | 209 | AAur_0407 | 210 | 40.78 | 102.53 | copper resistance protein, CopC family, putative |
| AAur_pTC10197 | 249 | AAur_3288 | 251 | 52.12 | 98.72 | cytochrome c biogenesis membrane protein |
| AAur_pTC10198 | 209 | AAur_3289 | 201 | 60.00 | 97.94 | Tat (twin-arginine translocation) domain protein, putative |
| AAur_pTC10199 | 194 | AAur_2370 | 292 | 57.81 | 100.00 | integral membrane protein, putative |
| AAur_pTC10202 | 264 | AAur_1047 | 263 | 36.84 | 100.42 | glutamine amidotransferases, putative |
| AAur_pTC10206 | 183 | AAur_3809 | 263 | 39.36 | 102.20 | oxidoreductase, short chain dehydrogenase/reductase family |
| AAur_pTC10218 | 490 | AAur_3399 | 448 | 35.53 | 101.70 | hydroxydechloroatrazine ethylaminohydrolase, putative |
| AAur_pTC10220 | 522 | AAur_0406 | 480 | 41.94 | 94.42 | xanthine/uracil permease family protein, putative |
| AAur_pTC10225 | 205 | AAur_0420 | 200 | 37.84 | 100.56 | site-specific recombinase, resolvase family |
| AAur_pTC10226 | 318 | AAur_1841 | 375 | 51.77 | 97.45 | prolipoprotein diacylglyceryl transferase |
| AAur_pTC10228 | 500 | AAur_1850 | 526 | 74.65 | 101.22 | multicopper oxidase family protein, putative |
| AAur_pTC10232 | 120 | AAur_3045 | 125 | 68.07 | 100.00 | thioredoxin |
| AAur_pTC10233 | 98 | AAur_0100 | 108 | 53.09 | 100.00 | rhodanese-related sulfurtransferase, putative |
| AAur_pTC10237 | 85 | AAur_4093 | 121 | 38.18 | 100.00 | protein of unknown function, putative |
| AAur_pTC10240 | 183 | AAur_3550 | 174 | 37.86 | 100.00 | redox-sensitive transcriptional activator SoxR |
| AAur_pTC10242 | 209 | AAur_0420 | 200 | 69.35 | 100.00 | site-specific recombinase, resolvase family |
| AAur_pTC10243 | 182 | AAur_0420 | 200 | 36.26 | 98.31 | site-specific recombinase, resolvase family |
| AAur_pTC10247 | 271 | AAur_2550 | 323 | 53.41 | 100.00 | ABC-type nitrate/sulfonate/taurine/bicarbonate transport systems, ATPase component, putative |
| AAur_pTC10248 | 257 | AAur_2551 | 259 | 35.66 | 100.00 | ABC-type nitrate/sulfonate transport system, permease component, putative |
| AAur_pTC10249 | 232 | AAur_2552 | 257 | 39.91 | 100.45 | ABC-type nitrate/sulfonate/bicarbonate transport system, permease component , putative |
| AAur_pTC10254 | 245 | AAur_4036 | 267 | 38.50 | 100.00 | transcriptional regulator, IclR family, putative |
| AAur_pTC10255 | 470 | AAur_1449 | 522 | 37.75 | 98.00 | glutamyl-tRNA(Gln) amidotransferase, A subunit |
| AAur_pTC10102 | 92 | AAur_1384 | 92 | 100.00 | 100.00 | ISAau1, transposase orfA |
| AAur_pTC10100 | 279 | AAur_1383 | 279 | 100.00 | 100.00 | ISAau1, transposase orfB |
| AAur_pTC10265 | 112 | AAur_0166 | 108 | 48.18 | 103.81 | conserved hypothetical protein |
| AAur_pTC10266 | 578 | AAur_2457 | 678 | 86.74 | 99.30 | thiamine biosynthesis |
| AAur_pTC10267 | 347 | AAur_2633 | 356 | 46.22 | 100.00 | membrane protein containing CBS domain |
| AAur_pTC10268 | 448 | AAur_2634 | 450 | 44.37 | 102.12 | integral membrane transporter with CBS domains |
| AAur_pTC10270 | 358 | AAur_2072 | 341 | 56.87 | 99.04 | membrane protein, tellurium resistance, putative |
| AAur_pTC10296 | 99 | AAur_0710 | 100 | 42.05 | 101.16 | Helix-turn-helix domain protein, putative |
| AAur_pTC10297 | 446 | AAur_0711 | 435 | 35.44 | 98.87 | HipA-like domains protein, putative |
| AAur_pTC10014 | 454 | AAur_2912 | 480 | 38.05 | 104.17 | Type II/IV secretion system protein, putative |
| AAur_pTC10034 | 333 | AAur_2440 | 311 | 35.04 | 108.80 | tyrosine recombinase XerC, putative |
| AAur_pTC10057 | 531 | AAur_3075 | 488 | 42.17 | 97.97 | ferredoxin/ferredoxin-NADP reductase, putative |
| AAur_pTC10058 | 371 | AAur_2317 | 384 | 36.36 | 101.41 | NAD/mycothiol-dependent formaldehyde dehydrogenase |
| AAur_pTC10063 | 450 | AAur_4011 | 477 | 38.11 | 98.23 | aldehyde dehydrogenase |
| AAur_pTC10064 | 257 | AAur_0095 | 251 | 35.25 | 102.42 | oxidoreductase, short chain dehydrogenase/reductase family |
| AAur_pTC10070 | 166 | AAur_2828 | 168 | 39.75 | 97.50 | transcriptional regulator, AsnC family |
| AAur_pTC10072 | 371 | AAur_3870 | 372 | 64.82 | 100.28 | alanine dehydrogenase |
| AAur_pTC10073 | 1126 | AAur_1686 | 1131 | 69.32 | 99.38 | pyruvate carboxylase |
| AAur_pTC10074 | 136 | AAur_2682 | 82 | 49.35 | 100.00 | transcription factor, WhiB family |
| AAur_pTC10078 | 363 | AAur_1488 | 627 | 36.45 | 97.35 | DNA primase |
| **pTC2** |  |  |  |  |  |  |
| AAur_pTC20036 | 278 | AAur_1018 | 278 | 41.50 | 99.64 | M23 peptidase domain protein |
| AAur_pTC20035 | 223 | AAur_2370 | 292 | 72.51 | 71.92 | integral membrane protein, putative |
| AAur_pTC20034 | 198 | AAur_3289 | 201 | 60.42 | 92.54 | Tat (twin-arginine translocation) domain protein, putative |
| AAur_pTC20033 | 221 | AAur_3288 | 251 | 52.56 | 83.67 | cytochrome c biogenesis membrane protein |
| AAur_pTC20031 | 651 | AAur_4095 | 809 | 38.19 | 43.39 | copper-translocating P-type ATPase |
| AAur_pTC20030 | 128 | AAur_3634 | 125 | 45.57 | 60.00 | transcriptional regulator, ArsR family, putative |
| AAur_pTC20028 | 713 | AAur_4095 | 809 | 35.99 | 86.65 | copper-translocating P-type ATPase |
| AAur_pTC20026 | 203 | AAur_3084 | 211 | 60.68 | 95.73 | secreted protein with unknown function, DUF305-family, putative |
| AAur_pTC20020 | 1634 | AAur_2465 | 1637 | 77.85 | 99.76 | hypothetical protein |
| AAur_pTC20019 | 348 | AAur_2466 | 342 | 78.89 | 99.42 | conserved hypothetical protein |
| AAur_pTC20008 | 209 | AAur_0420 | 200 | 89.42 | 94.00 | site-specific recombinase, resolvase family |
| AAur_pTC20259 | 265 | AAur_2326 | 379 | 47.66 | 61.74 | protein of unknown function (DUF1016), putative |
| AAur_pTC20258 | 764 | AAur_1657 | 319 | 38.00 | 30.72 | tyrosine recombinase XerD |
| AAur_pTC20256 | 349 | AAur_2326 | 379 | 39.52 | 86.81 | protein of unknown function (DUF1016), putative |
| AAur_pTC20245 | 270 | AAur_3317 | 483 | 38.96 | 50.93 | fatty aldehyde dehydrogenase |
| AAur_pTC20243 | 189 | AAur_3995 | 179 | 51.77 | 75.42 | transcriptional regulator, MarR family, putative |
| AAur_pTC20242 | 247 | AAur_1145 | 302 | 52.84 | 75.17 | ANTAR domain protein |
| AAur_pTC20241 | 85 | AAur_3349 | 96 | 38.71 | 63.54 | conserved hypothetical protein |
| AAur_pTC20237 | 447 | AAur_3513 | 373 | 41.25 | 68.63 | two-component system response regulator |
| AAur_pTC20233 | 323 | AAur_1083 | 126 | 46.39 | 76.19 | hypothetical protein |
| AAur_pTC20231 | 342 | AAur_2794 | 366 | 62.28 | 93.17 | UDP-glucose 4-epimerase |
| AAur_pTC20227 | 266 | AAur_2852 | 310 | 73.48 | 84.52 | UTP-glucose-1-phosphate uridylyltransferase |
| AAur_pTC20226 | 259 | AAur_1074 | 247 | 53.70 | 103.64 | two-component system response regulator |
| AAur_pTC20225 | 188 | AAur_0420 | 200 | 35.08 | 89.50 | site-specific recombinase, resolvase family |
| AAur_pTC20222 | 122 | AAur_4187 | 131 | 37.74 | 79.39 | transcriptional regulator protein, putative |
| AAur_pTC20220 | 423 | AAur_3553 | 405 | 48.08 | 95.80 | Metal-dependent amidase/aminoacylase/carboxypeptidase, putative |
| AAur_pTC20218 | 333 | AAur_2317 | 384 | 37.76 | 84.90 | NAD/mycothiol-dependent formaldehyde dehydrogenase |
| AAur_pTC20217 | 419 | AAur_1939 | 439 | 37.28 | 89.98 | aminotransferase class-III protein |
| AAur_pTC20216 | 498 | AAur_2547 | 590 | 37.23 | 77.46 | amino acid permease, putative |
| AAur_pTC20215 | 455 | AAur_0809 | 492 | 38.53 | 91.67 | aldehyde dehydrogenase (NAD) family protein |
| AAur_pTC20212 | 257 | AAur_2422 | 261 | 36.29 | 95.79 | Gluconate 5-dehydrogenase (5-keto-D-gluconate 5-reductase) |
| AAur_pTC20211 | 246 | AAur_1047 | 263 | 35.89 | 72.62 | glutamine amidotransferases, putative |
| AAur_pTC20204 | 371 | AAur_3870 | 372 | 85.44 | 99.19 | alanine dehydrogenase |
| AAur_pTC20203 | 160 | AAur_2828 | 168 | 37.93 | 85.71 | transcriptional regulator, AsnC family |
| AAur_pTC20201 | 315 | AAur_2621 | 322 | 36.94 | 95.96 | transcriptional regulator, AraC family |
| AAur_pTC20196 | 507 | AAur_3064 | 507 | 92.31 | 99.80 | aldehyde dehydrogenase (NAD) family protein |
| AAur_pTC20195 | 174 | AAur_3062 | 138 | 73.57 | 100.73 | conserved hypothetical protein |
| AAur_pTC20193 | 279 | AAur_1383 | 279 | 100.00 | 99.64 | ISAau1, transposase orfB |
| AAur_pTC20192 | 644 | AAur_1378 | 646 | 84.09 | 99.07 | amine oxidase (copper-containing) |
| AAur_pTC20189 | 676 | AAur_3353 | 685 | 85.60 | 92.12 | acetate--CoA ligase |
| AAur_pTC20188 | 133 | AAur_0495 | 717 | 65.71 | 14.23 | conserved hypothetical protein |
| AAur_pTC20183 | 398 | AAur_2004 | 398 | 51.76 | 99.75 | pyridine nucleotide-disulphide oxidoreductase domain protein |
| AAur_pTC20182 | 161 | AAur_3367 | 178 | 51.19 | 88.76 | endoribonuclease L-PSP family, putative |
| AAur_pTC20181 | 369 | AAur_2628 | 360 | 59.77 | 96.39 | Pyridoxal-phosphate dependent enzyme |
| AAur_pTC20180 | 452 | AAur_0577 | 452 | 36.28 | 92.48 | major facilitator superfamily (MFS) transporter, putative |
| AAur_pTC20177 | 227 | AAur_0050 | 232 | 40.91 | 84.91 | transcriptional regulator, GntR family |
| AAur_pTC20173 | 93 | AAur_2094 | 518 | 92.47 | 17.76 | glucose-6-phosphate 1-dehydrogenase |
| AAur_pTC20167 | 70 | AAur_4094 | 77 | 42.19 | 77.92 | heavy metal-associated domain protein |
| AAur_pTC20165 | 770 | AAur_4095 | 809 | 58.18 | 80.22 | copper-translocating P-type ATPase |
| AAur_pTC20161 | 133 | AAur_2561 | 133 | 41.12 | 79.70 | integral membrane protein, putative |
| AAur_pTC20158 | 213 | AAur_3028 | 255 | 35.39 | 69.02 | transcriptional regulator, TetR family, putative |
| AAur_pTC20157 | 827 | AAur_0658 | 831 | 42.19 | 96.63 | Beta-glucosidase |
| AAur_pTC20154 | 633 | AAur_3353 | 685 | 40.44 | 90.37 | acetate--CoA ligase |
| AAur_pTC20153 | 218 | AAur_1663 | 213 | 83.49 | 99.06 | transcriptional regulator, GntR family |
| AAur_pTC20152 | 506 | AAur_1662 | 506 | 88.74 | 99.80 | 2-methylcitrate dehydratase, putative |
| AAur_pTC20151 | 301 | AAur_1661 | 301 | 86.96 | 99.00 | methylisocitrate lyase |
| AAur_pTC20150 | 380 | AAur_1660 | 379 | 88.44 | 97.89 | citrate synthase |
| AAur_pTC20149 | 192 | AAur_2928 | 192 | 85.25 | 94.79 | Adenylate kinase (ATP-AMP transphosphorylase) |
| AAur_pTC20139 | 119 | AAur_1157 | 167 | 36.90 | 49.70 | transcriptional regulator, ArsR family, putative |
| AAur_pTC20134 | 311 | AAur_3313 | 314 | 68.79 | 98.73 | ABC-type sugar transport system, permease component, putative |
| AAur_pTC20133 | 310 | AAur_3312 | 313 | 66.13 | 98.72 | ABC-type sugar transport system, permease component, putative |
| AAur_pTC20132 | 426 | AAur_3311 | 456 | 44.52 | 92.76 | extracellular solute-binding domain protein |
| AAur_pTC20131 | 375 | AAur_1140 | 368 | 55.11 | 95.11 | oxidoreductase family, NAD-binding Rossmann fold domain protein |
| AAur_pTC20130 | 309 | AAur_1141 | 320 | 48.09 | 95.94 | conserved hypothetical protein |
| AAur_pTC20129 | 250 | AAur_1142 | 238 | 46.38 | 96.64 | cupin domain protein, putative |
| AAur_pTC20125 | 141 | AAur_3720 | 143 | 83.57 | 97.20 | aspartate 1-decarboxylase |
| AAur_pTC20124 | 160 | AAur_3775 | 163 | 43.83 | 97.55 | flavin reductase like domain, putative |
| AAur_pTC20115 | 503 | AAur_0308 | 490 | 36.70 | 93.67 | drug resistance transporter, EmrB/QacA subfamily, putative |
| AAur_pTC20113 | 362 | AAur_3723 | 329 | 46.74 | 79.03 | luciferase-like monooxygenase, putative |
| AAur_pTC20110 | 329 | AAur_2506 | 539 | 40.27 | 53.99 | D-3-phosphoglycerate dehydrogenase |
| AAur_pTC20109 | 438 | AAur_0577 | 452 | 39.41 | 88.72 | major facilitator superfamily (MFS) transporter, putative |
| AAur_pTC20107 | 114 | AAur_3626 | 470 | 90.27 | 23.83 | phenylacetaldehyde dehydrogenase |
| AAur_pTC20103 | 321 | AAur_2548 | 272 | 40.66 | 99.63 | transcriptional regulator, AraC family |
| AAur_pTC20102 | 469 | AAur_3626 | 470 | 84.89 | 99.57 | phenylacetaldehyde dehydrogenase |
| AAur_pTC20098 | 509 | AAur_2043 | 485 | 36.17 | 97.73 | amino acid permease, putative |
| AAur_pTC20096 | 469 | AAur_3626 | 470 | 81.70 | 99.57 | phenylacetaldehyde dehydrogenase |
| AAur_pTC20095 | 506 | AAur_1331 | 493 | 73.93 | 99.39 | succinate-semialdehyde dehydrogenase, putative |
| AAur_pTC20094 | 267 | AAur_3912 | 267 | 73.78 | 99.63 | 2,4-dihydroxyhept-2-ene-1,7-dioic acid aldolase (HpaI), putative |
| AAur_pTC20093 | 228 | AAur_3913 | 261 | 93.39 | 86.59 | 2-oxo-hepta-3-ene-1,7-dioic acid hydratase |
| AAur_pTC20092 | 360 | AAur_3914 | 365 | 91.39 | 98.08 | 3,4-dihydroxyphenylacetate 2,3-dioxygenase |
| AAur_pTC20091 | 513 | AAur_3915 | 503 | 74.10 | 99.60 | 5-carboxymethyl-2-hydroxymuconate semialdehyde dehydrogenase |
| AAur_pTC20090 | 226 | AAur_3916 | 225 | 85.84 | 100.00 | transcriptional regulater, GntR family |
| AAur_pTC20089 | 470 | AAur_3917 | 489 | 87.11 | 95.91 | fumarylacetoacetate hydrolase family protein |
| AAur_pTC20088 | 638 | AAur_2032 | 644 | 83.91 | 98.29 | FAD monooxygenase, PheA/TfdB family, putative |
| AAur_pTC20087 | 484 | AAur_2547 | 590 | 36.15 | 76.27 | amino acid permease, putative |
| AAur_pTC20083 | 197 | AAur_3624 | 196 | 68.04 | 98.47 | transcriptional regulator, TetR family, putative |
| AAur_pTC20082 | 654 | AAur_3623 | 660 | 89.69 | 96.67 | amine oxidase (copper-containing) |
| AAur_pTC20073 | 171 | AAur_4164 | 193 | 81.33 | 84.97 | single-strand binding protein |
| AAur_pTC20071 | 94 | AAur_2341 | 80 | 54.05 | 91.25 | Glutaredoxin-like protein NrdH |
| AAur_pTC20067 | 94 | AAur_3237 | 127 | 62.03 | 61.42 | hypothetical protein |
| AAur_pTC20062 | 138 | AAur_3630 | 140 | 40.30 | 95.00 | arsenate reductase |
| AAur_pTC20061 | 218 | AAur_3630 | 140 | 61.72 | 90.71 | arsenate reductase |
| AAur_pTC20060 | 509 | AAur_3628 | 459 | 80.62 | 98.69 | FAD dependent oxidoreductase domain protein, putative |
| AAur_pTC20059 | 329 | AAur_3629 | 325 | 94.14 | 99.38 | thioredoxin-disulfide reductase |
| AAur_pTC20058 | 140 | AAur_3630 | 140 | 93.57 | 99.29 | arsenate reductase |
| AAur_pTC20057 | 224 | AAur_3631 | 124 | 69.35 | 99.19 | arsenate reductase, putative |
| AAur_pTC20056 | 246 | AAur_3632 | 275 | 80.17 | 87.64 | MIP family protein |
| AAur_pTC20055 | 115 | AAur_3634 | 125 | 79.31 | 91.20 | transcriptional regulator, ArsR family, putative |
| AAur_pTC20054 | 162 | AAur_3635 | 169 | 69.62 | 92.90 | phosphinothricin N-acetyltransferase, putative |
| AAur_pTC20053 | 115 | AAur_3326 | 186 | 38.71 | 32.80 | transcriptional regulator, ArsR family, putative |
| AAur_pTC20049 | 236 | AAur_2648 | 237 | 46.26 | 94.09 | Iron dependent repressor, putative |
| AAur_pTC20047 | 220 | AAur_0407 | 210 | 36.49 | 94.76 | copper resistance protein, CopC family, putative |
| AAur_pTC20046 | 202 | AAur_0784 | 206 | 37.50 | 93.20 | hypothetical protein |
| AAur_pTC20045 | 230 | AAur_1365 | 222 | 71.89 | 97.30 | DSBA-like thioredoxin domain protein, putative |
| AAur_pTC20044 | 305 | AAur_0412 | 306 | 50.00 | 98.69 | cobalt-zinc-cadmium efflux permease |
| AAur_pTC20043 | 125 | AAur_1363 | 108 | 62.86 | 96.30 | transcriptional regulator, ArsR family |
| AAur_pTC20042 | 701 | AAur_3742 | 751 | 55.59 | 92.28 | copper resistance protein D family, putative |
| AAur_pTC20041 | 141 | AAur_3634 | 125 | 39.02 | 62.40 | transcriptional regulator, ArsR family, putative |
| AAur_pTC20039 | 543 | AAur_3287 | 564 | 72.69 | 91.84 | cytochrome c biogenesis membrane protein, ResB-like family, putative |
| AAur_pTC20038 | 285 | AAur_2117 | 288 | 48.95 | 97.57 | lipoprotein, putative |
| AAur_pTC20194 | 101 | AAur_3175 | 101 | 100.00 | 99.01 | ISAau1, transposase orfA |
