# Supplementary material for: Secrets of Soil Survival Revealed by the Genome Sequence of Arthrobacter aurescens TC1
Source: PLoS Genet. 2006 Dec 22;2(12):e214. doi: 10.1371/journal.pgen.0020214 (PMC1713258; doi:10.1371/journal.pgen.0020214)
Supplement: Supplementary file 7 [file pgen.0020214.st004.doc]

**Supplemental Table 4**. List of the genes shared between the *A. aurescens* pTC1, *Pseudomonas* sp. pADP1 and *A. nicotinivorans* pAO1 plasmids.

| **Reference ORF** | **Predicted Protein Product** | **Gene symbol** | **Length (bp)** | **Query ORF** | **Predicted Protein Product** | **Gene symbol** | **Length (bp)** | **% Sim** |
| --- | --- | --- | --- | --- | --- | --- | --- | --- |
| ***A. aurescens* pTC1 vs. *Pseudomonas* sp. ADP pADP1:** | | | | | | | | |
| AAur_pTC10087 | triazine hydrolase | *trzN* | 1371 | AAC45138.1 | hydroxyatrazine hydrolase second enzyme of a novel atrazine degradation | *atzB* | 1445 | 32.02 |
| AAur_pTC10131 | heavy metal-associated domain protein | *--* | 996 | AAK50287.1 | MerP protein | *merP* | 275 | 34.29 |
| AAur_pTC10132 | copper-translocating P-type ATPase | *--* | 2322 | AAK50287.1 | MerP protein | *merP* | 275 | 34.67 |
| AAur_pTC10138 | COP associated protein (Copper ion binding pprotein) | *--* | 336 | AAK50287.1 | MerP protein | *merP* | 275 | 30.00 |
| AAur_pTC10202 | putative glutamine amidotransferase | *--* | 795 | AAK50292.1 | putative glutamine amidotransferase | *--* | 794 | 100.00 |
| AAur_pTC10203 | putative Lipoprotein | *--* | 474 | AAK50293.1 | hypothetical protein | *--* | 473 | 100.00 |
| AAur_pTC10204 | putative Integral membrane protein DUF6 | *--* | 948 | AAK50294.1 | hypothetical protein | *--* | 947 | 100.00 |
| AAur_pTC10205 | Conserved hypothetical protein | *--* | 804 | AAK50295.1 | hypothetical protein | *--* | 803 | 100.00 |
| AAur_pTC10206 | hypothetical protein | *--* | 552 | AAK50297.1 | putative short chain dehydrogenase | *--* | 443 | 99.32 |
| AAur_pTC10207 | putative Transcriptional regulator, TetR family | *--* | 567 | AAK50298.1 | putative transcriptional regulator | *--* | 566 | 100.00 |
| AAur_pTC10209 | IS1380 family transposase | *--* | 1311 | AAK50300.1 | putative transposase | *tnpA* | 1310 | 100.00 |
| AAur_pTC10211 | IS5 family transposase | *--* | 870 | AAK50302.1 | putative transposase | *tnpA* | 968 | 99.65 |
| AAur_pTC10212 | N-isopropylammelide isopropylaminohydrolase | *atzC* | 1224 | AAB96621.1 | N-isopropylammelide isopropylamino hydrolase transforms N-isopropylammelide to cyanuric acid | *atzC* | 1211 | 99.02 |
| AAur_pTC10216 | IS1071, transposase, truncation | *--* | 1392 | AAK50281.1 | putative transposase | *tnpA* | 2915 | 100.00 |
| AAur_pTC10218 | hydroxyatrazine hydrolase | *atzB* | 1473 | AAC45138.1 | hydroxyatrazine hydrolase second enzyme of a novel atrazine degradation | *atzB* | 1445 | 100.00 |
| AAur_pTC10217 | putative Transcriptional regulator, TetR family | *--* | 576 | AAK50277.1 | putative transcriptional regulator | *--* | 575 | 100.00 |
| AAur_pTC10220 | putative Xanthine/uracil permease family protein | *--* | 1569 | AAK50278.1 | putative transporter | *--* | 1340 | 100.00 |
| AAur_pTC10222 | ISPps1, transposase | *--* | 1548 | AAK50279.1 | putative transposase | *tnpA* | 1547 | 100.00 |
| AAur_pTC10223 | dihydrolipoamide dehydrogenase, truncation | *--* | 1203 | AAK50266.1 | dihyrolipoamide dehydrogenase-like protein | *--* | 1202 | 100.00 |
| AAur_pTC10224 | IS1071, transposase | *--* | 2916 | AAK50281.1 | putative transposase | *tnpA* | 2915 | 100.00 |
| AAur_pTC10225 | resolvase, N terminal domain | *--* | 618 | AAK50282.1 | putative resolvase | *tnpR* | 614 | 83.33 |
| AAur_pTC10241 | mercuric reductase | *merA* | 1443 | AAK50285.1 | MerA protein | *merA* | 1646 | 43.76 |
| AAur_pTC10242 | invertase recombinase-like protein | *--* | 630 | AAK50282.1 | putative resolvase | *tnpR* | 614 | 42.03 |
| AAur_pTC10243 | DNA-invertase (Site-specific recombinase resolvase family) | *--* | 549 | AAK50282.1 | putative resolvase | *tnpR* | 614 | 37.10 |
| AAur_pTC10247 | putative ABC transporter, ATP-binding protein | *--* | 816 | AAK50327.1 | putative-ATP binding domain of ABC transporter | *--* | 1622 | 30.23 |
| AAur_pTC10255 | putative Amidase | *--* | 1413 | AAK50332.1 | biuret hydrolase | *atzE* | 1373 | 32.75 |
| AAur_pTC10275 | hypothetical protein | *--* | 1026 | AAK50247.1 | KrfA protein | *krfA* | 1031 | 30.36 |
| AAur_pTC10014 | putative Type II/IV secretion system protein | *--* | 1365 | AAK50319.1 | TrbB protein | *trbB* | 962 | 30.38 |
| AAur_pTC10051 | IS21 family element, transposase istB | *--* | 804 | AAK50261.1 | IstB homolog | *--* | 848 | 36.93 |
| AAur_pTC10065 | putative glutamine amidotransferase | *--* | 726 | AAK50292.1 | putative glutamine amidotransferase | *--* | 794 | 38.65 |
| AAur_pTC10076 | hypothetical protein | *--* | 975 | AAK50309.1 | TrbL protein | *trbL* | 1718 | 40.38 |
| AAur_pTC10210 | putative integral membrane protein | *--* | 1146 | AAK50301.1 | putative transporter | *--* | 1145 | 100.00 |
| AAur_pTC10215 | degenerate dihydrolipoamide dehydrogenase/degenerate ISPps1 transposase fusion | *--* | 1884 | AAK50305.1 | dihydrolipoamide dehydrogenase homolog | *--* | 1499 | 81.82 |
| ***A. aurescens* pTC1 vs. *A. nicotinivorans* pAO1:** | | | | | | | | |
| AAur_pTC10093 | Putative Soj/ParA family protein | *--* | 942 | CAD48001.1 | putative soj family protein | *--* | 944 | 87.91 |
| AAur_pTC10094 | Conserved hypothetical protein | *--* | 753 | CAD48000.1 | hypothetical protein | *--* | 695 | 48.97 |
| AAur_pTC10095 | hypothetical protein | *--* | 1926 | CAD47997.1 | hypothetical protein | *--* | 1961 | 69.79 |
| AAur_pTC10096 | conserved hypothetical protein | *--* | 237 | CAD47996.1 | hypothetical protein | *--* | 650 | 62.82 |
| AAur_pTC10262 | ISAau1, transposase orfB | *--* | 840 | CAD47998.1 | putative transposase | *--* | 725 | 30.71 |
| AAur_pTC10261 | ISAau1, transposase orfA | *--* | 279 | CAD48016.1 | hypothetical protein | *--* | 290 | 38.46 |
| AAur_pTC10104 | Conserved hypothetical protein | *--* | 1146 | CAD47994.1 | hypothetical protein | *--* | 1166 | 33.42 |
| AAur_pTC10105 | Conserved hypothetical protein | *--* | 585 | CAD47991.1 | hypothetical protein | *--* | 1073 | 67.76 |
| AAur_pTC10106 | M23 peptidase domain protein | *--* | 1077 | CAD47991.1 | hypothetical protein | *--* | 1073 | 48.26 |
| AAur_pTC10107 | putative Lipoprotein | *--* | 522 | CAD47990.1 | hypothetical protein | *--* | 512 | 30.23 |
| AAur_pTC10108 | Conserved hypothetical protein | *--* | 1194 | CAD47989.1 | hypothetical protein | *--* | 1442 | 30.60 |
| AAur_pTC10115 | hypothetical protein | *--* | 384 | CAD47981.1 | hypothetical protein | *--* | 440 | 33.58 |
| AAur_pTC10124 | putative Partitioning protein, parB-family | *--* | 1686 | CAD47865.1 | PAR putative chromosome partitioning protein | *--* | 620 | 51.44 |
| AAur_pTC10225 | resolvase, N terminal domain | *--* | 618 | CAD48008.1 | putative recombinase | *--* | 578 | 49.72 |
| AAur_pTC10242 | invertase recombinase-like protein | *--* | 630 | CAD48008.1 | putative recombinase | *--* | 578 | 38.86 |
| AAur_pTC10243 | DNA-invertase (Site-specific recombinase resolvase family) | *--* | 549 | CAD48011.1 | putative DNA recombinase | *--* | 503 | 70.73 |
| AAur_pTC10247 | putative ABC transporter, ATP-binding protein | *--* | 816 | CAD47964.1 | MODC molybdenum transport ATPase modC | *--* | 1049 | 34.36 |
| AAur_pTC10102 | ISAau1, transposase orfA | *--* | 279 | CAD48016.1 | hypothetical protein | *--* | 290 | 38.46 |
| AAur_pTC10100 | ISAau1, transposase orfB | *--* | 840 | CAD47998.1 | putative transposase | *--* | 725 | 30.71 |
| AAur_pTC10272 | hypothetical protein | *--* | 177 | CAD47931.1 | hypothetical protein | *--* | 353 | 39.13 |
| AAur_pTC10275 | hypothetical protein | *--* | 1026 | CAD47876.1 | putative kfrA-like protein | *--* | 680 | 32.26 |
| AAur_pTC10296 | putative Transcriptional regulator, helix-turn-helix XRE-family | *--* | 300 | CAD47888.1 | hypothetical protein | *--* | 287 | 42.05 |
| AAur_pTC10297 | Conserved hypothetical protein | *--* | 1341 | CAD47889.1 | hypothetical protein | *--* | 1376 | 37.70 |
| AAur_pTC10001 | putative ATPase, ParA-family | *--* | 1122 | CAD48001.1 | putative soj family protein | *--* | 944 | 37.79 |
| AAur_pTC10033 | Conserved hypothetical protein | *--* | 360 | CAD47911.1 | hypothetical protein | *--* | 305 | 30.95 |
| AAur_pTC10063 | Aldehyde dehydrogenase (NAD) family protein | *--* | 1353 | CAD47916.1 | CGL putative NAD-dependent aldehyde dehydrogenase | *--* | 1352 | 35.14 |
