# Supplementary material for: Secrets of Soil Survival Revealed by the Genome Sequence of Arthrobacter aurescens TC1
Source: PLoS Genet. 2006 Dec 22;2(12):e214. doi: 10.1371/journal.pgen.0020214 (PMC1713258; doi:10.1371/journal.pgen.0020214)
Supplement: Supplementary file 8 [file pgen.0020214.st005.doc]

**Supplemental Table 5**. List of the *Arthrobacter aurescens* TC1 uniques genes, compared to the genome of *Arthrobacter*

*sp.* FB24.

| **Gene** | **Gene Length** | **End5** | **End3** | **Gene**  **Symbol** | **EC Number** | **Functional Gene Name** |
| --- | --- | --- | --- | --- | --- | --- |
| AAur_0020 | 735 | 19650 | 18916 | *--* | -- | putative DNA repair protein |
| AAur_0038 | 255 | 35644 | 35898 | *--* | -- | hypothetical protein |
| AAur_0039 | 513 | 36851 | 36339 | *--* | -- | putative D-mannose binding-like domain |
| AAur_0041 | 825 | 37967 | 38791 | *--* | -- | hypothetical protein |
| AAur_0063 | 930 | 63330 | 62401 | *--* | -- | putative integral membrane protein |
| AAur_0066 | 312 | 64613 | 64302 | *--* | -- | hypothetical protein |
| AAur_0071 | 243 | 69932 | 69690 | *--* | -- | conserved hypothetical protein |
| AAur_0072 | 444 | 70027 | 70470 | *--* | -- | conserved hypothetical protein |
| AAur_0074 | 858 | 72837 | 71980 | *--* | -- | putative membrane protein |
| AAur_0075 | 447 | 73347 | 72901 | *--* | -- | conserved hypothetical protein |
| AAur_0076 | 450 | 73793 | 73344 | *--* | -- | conserved hypothetical protein |
| AAur_0077 | 216 | 74317 | 74102 | *--* | -- | hypothetical protein |
| AAur_0106 | 855 | 109397 | 110251 | *--* | -- | putative transcriptional regulator, AraC family domain protein |
| AAur_0126 | 636 | 128786 | 128151 | *--* | -- | hypothetical protein |
| AAur_0127 | 636 | 129408 | 128773 | *--* | -- | hypothetical protein |
| AAur_0128 | 777 | 130232 | 129456 | *--* | -- | hypothetical protein |
| AAur_0135 | 111 | 136789 | 136679 | *--* | -- | hypothetical protein |
| AAur_0149 | 123 | 150038 | 149916 | *--* | -- | hypothetical protein |
| AAur_0153 | 546 | 156696 | 156151 | *--* | -- | hypothetical protein |
| AAur_0159 | 969 | 164321 | 165289 | *--* | -- | Type I phosphodiesterase / nucleotide pyrophosphatase family protein |
| AAur_0160 | 858 | 165286 | 166143 | *--* | -- | putative Integral membrane protein DUF6 |
| AAur_0168 | 1209 | 173989 | 175197 | *--* | -- | putative glucosyltransferase |
| AAur_0172 | 189 | 179699 | 179887 | *--* | -- | hypothetical protein |
| AAur_0180 | 813 | 183981 | 184793 | *--* | -- | transcriptional regulator, AraC family |
| AAur_0181 | 153 | 184957 | 185109 | *--* | -- | hypothetical protein |
| AAur_0201 | 774 | 207922 | 208695 | *--* | -- | hypothetical protein |
| AAur_0226 | 231 | 230844 | 231074 | *--* | -- | hypothetical protein |
| AAur_0227 | 1023 | 232171 | 231149 | *--* | -- | putative ATP/GTP-binding protein |
| AAur_0236 | 1452 | 240296 | 238845 | *--* | -- | putative pectate lyase |
| AAur_0237 | 1983 | 242280 | 240298 | *--* | -- | putative pectinesterase/pectate lyase protein |
| AAur_0242 | 1581 | 246717 | 248297 | *--* | 3.2.1.- | xylosidase/arabinosidase |
| AAur_0248 | 1242 | 256540 | 255299 | *--* | -- | putative oxidoreductase |
| AAur_0250 | 150 | 257646 | 257497 | *--* | -- | hypothetical protein |
| AAur_0262 | 582 | 269274 | 268693 | *--* | -- | hypothetical protein |
| AAur_0265 | 360 | 274482 | 274123 | *--* | -- | hypothetical protein |
| AAur_0270 | 1320 | 281471 | 280152 | *--* | -- | hypothetical protein |
| AAur_0272 | 432 | 282398 | 282829 | *--* | -- | putative lipoprotein |
| AAur_0275 | 516 | 284540 | 285055 | *--* | -- | acetyltransferase, GNAT family protein |
| AAur_0276 | 402 | 285457 | 285056 | *--* | -- | conserved hypothetical protein |
| AAur_0288 | 222 | 297946 | 297725 | *--* | -- | hypothetical protein |
| AAur_0291 | 558 | 299776 | 300333 | *--* | -- | putative integral membrane protein |
| AAur_0297 | 306 | 305941 | 305636 | *--* | -- | hypothetical protein |
| AAur_0298 | 453 | 306357 | 305905 | *--* | -- | hypothetical protein |
| AAur_0307 | 354 | 315487 | 315134 | *--* | -- | hypothetical protein |
| AAur_0310 | 756 | 317764 | 318519 | *--* | -- | putative methyltransferase |
| AAur_0315 | 918 | 324575 | 323658 | *--* | -- | putative lycopene elongase |
| AAur_0317 | 351 | 325210 | 324860 | *--* | -- | putative C50 carotenoid epsilon cyclase |
| AAur_0319 | 906 | 327764 | 326859 | *crtB* | 2.5.1.- | phytoene synthase |
| AAur_0321 | 555 | 329421 | 328867 | *idi* | 5.3.3.2 | isopentenyl-diphosphate delta-isomerase, type 1 |
| AAur_0327 | 1389 | 333560 | 334948 | *--* | -- | putative integral membrane protein |
| AAur_0338 | 471 | 347741 | 347271 | *--* | -- | hypothetical protein |
| AAur_0358 | 261 | 370302 | 370042 | *--* | -- | hypothetical protein |
| AAur_0359 | 372 | 370781 | 371152 | *--* | -- | domain of unknown function (306) |
| AAur_0361 | 408 | 372621 | 372214 | *--* | -- | hypothetical protein |
| AAur_0377 | 402 | 384791 | 384390 | *--* | -- | hypothetical protein |
| AAur_0378 | 1167 | 384845 | 386011 | *--* | -- | putative cupin superfamily protein |
| AAur_0379 | 909 | 386021 | 386929 | *--* | -- | conserved hypothetical protein |
| AAur_0380 | 156 | 387117 | 386962 | *--* | -- | hypothetical protein |
| AAur_0384 | 828 | 391717 | 392544 | *--* | -- | transcriptional regulator, AraC family, authentic FRAMESHIFT |
| AAur_0397 | 438 | 407602 | 407165 | *--* | -- | hypothetical protein |
| AAur_0419 | 627 | 432000 | 431374 | *--* | -- | putative integral membrane protein |
| AAur_0421 | 450 | 433372 | 432923 | *--* | -- | putative histidine triad protein ( HIT domain) |
| AAur_0422 | 777 | 434145 | 433369 | *--* | -- | conserved hypothetical protein |
| AAur_0423 | 510 | 434723 | 434214 | *--* | -- | conserved hypothetical protein |
| AAur_0424 | 129 | 435581 | 435453 | *--* | -- | hypothetical protein |
| AAur_0425 | 1371 | 436999 | 435629 | *--* | -- | hypothetical protein |
| AAur_0426 | 909 | 437823 | 436915 | *--* | -- | hypothetical protein |
| AAur_0427 | 1113 | 440833 | 439721 | *--* | -- | putative protein of unknown function (DUF1526) |
| AAur_0428 | 726 | 442263 | 441538 | *--* | -- | putative lipoprotein |
| AAur_0430 | 2400 | 444885 | 442486 | *--* | -- | hypothetical protein |
| AAur_0431 | 228 | 445212 | 444985 | *--* | -- | putative integral membrane protein |
| AAur_0432 | 327 | 445579 | 445253 | *--* | -- | putative lipoprotein |
| AAur_0433 | 747 | 446358 | 445612 | *--* | -- | hypothetical protein |
| AAur_0434 | 993 | 447634 | 446642 | *--* | -- | putative integral membrane protein |
| AAur_0437 | 567 | 451522 | 450956 | *--* | -- | putative transcriptional regulator, TetR family |
| AAur_0438 | 468 | 451989 | 451522 | *--* | -- | putative protein of unknown function (DUF1348) |
| AAur_0440 | 1002 | 453353 | 454354 | *--* | -- | putative SIS domain protein |
| AAur_0458 | 402 | 468434 | 468033 | *--* | -- | putative helix-turn-helix domain protein |
| AAur_0476 | 189 | 484301 | 484113 | *--* | -- | hypothetical protein |
| AAur_0483 | 627 | 494351 | 493725 | *--* | -- | putative formiminotetrahydrofolate cyclodeaminase |
| AAur_0498 | 450 | 511051 | 511500 | *--* | -- | hypothetical protein |
| AAur_0514 | 771 | 530423 | 529653 | *--* | -- | putative lipoprotein |
| AAur_0539 | 114 | 556898 | 557011 | *--* | -- | hypothetical protein |
| AAur_0540 | 1110 | 557023 | 558132 | *--* | -- | hypothetical protein |
| AAur_0541 | 933 | 558172 | 559104 | *--* | -- | conserved hypothetical protein |
| AAur_0542 | 174 | 559216 | 559389 | *--* | -- | hypothetical protein |
| AAur_0543 | 174 | 559506 | 559679 | *--* | -- | hypothetical protein |
| AAur_0544 | 654 | 559774 | 560427 | *--* | -- | Flavoprotein domain protein |
| AAur_0545 | 864 | 560424 | 561287 | *--* | -- | hypothetical protein |
| AAur_0546 | 1086 | 561452 | 562537 | *--* | -- | putative peptidase M16 family |
| AAur_0547 | 378 | 562928 | 562551 | *--* | -- | conserved hypothetical protein |
| AAur_0574 | 672 | 591730 | 591059 | *--* | -- | putative Family of unknown function (DUF1028) |
| AAur_0575 | 423 | 592149 | 591727 | *--* | -- | putative endoribonuclease L-PSP family |
| AAur_0591 | 1722 | 612224 | 610503 | *--* | -- | putative PHP domain protein |
| AAur_0598 | 435 | 618879 | 618445 | *--* | -- | hypothetical protein |
| AAur_0599 | 681 | 619688 | 619008 | *--* | -- | hypothetical protein |
| AAur_0600 | 459 | 619859 | 620317 | *--* | -- | conserved hypothetical protein |
| AAur_0602 | 1068 | 621938 | 623005 | *--* | -- | putative saccharopine dehydrogenase |
| AAur_0604 | 1275 | 623613 | 624887 | *--* | -- | conserved hypothetical protein |
| AAur_0617 | 372 | 635728 | 635357 | *--* | -- | conserved hypothetical protein |
| AAur_0624 | 510 | 641833 | 641324 | *--* | -- | hypothetical protein |
| AAur_0625 | 333 | 642155 | 641823 | *--* | -- | conserved hypothetical protein |
| AAur_0627 | 504 | 643483 | 643986 | *--* | -- | hypothetical protein |
| AAur_0633 | 309 | 649742 | 649434 | *--* | -- | hypothetical protein |
| AAur_0634 | 969 | 650777 | 649809 | *--* | 1.11.1.6 | catalase, Mn-containing |
| AAur_0648 | 540 | 665780 | 666319 | *--* | -- | putative formaldehyde-activating enzyme |
| AAur_0664 | 990 | 681697 | 682686 | *--* | -- | putative integral membrane protein |
| AAur_0670 | 1074 | 688326 | 689399 | *--* | -- | putative acyltransferase |
| AAur_0679 | 945 | 702065 | 703009 | *--* | -- | hypothetical protein |
| AAur_0680 | 858 | 703014 | 703871 | *--* | -- | hypothetical protein |
| AAur_0707 | 261 | 732869 | 732609 | *--* | -- | addiction module toxin, RelE/StbE |
| AAur_0708 | 267 | 733132 | 732866 | *--* | -- | prevent-host-death protein |
| AAur_0711 | 1308 | 734663 | 735970 | *--* | -- | putative HipA-like domains protein |
| AAur_0732 | 765 | 759192 | 759956 | *--* | -- | conserved hypothetical protein |
| AAur_0741 | 408 | 767808 | 767401 | *--* | -- | endoribonuclease L-PSP family, putative |
| AAur_0743 | 1218 | 770545 | 769328 | *--* | -- | conserved hypothetical protein |
| AAur_0749 | 1257 | 776798 | 778054 | *--* | -- | putative N-carbamoyl-L-amino acid amidohydrolase |
| AAur_0758 | 870 | 788792 | 787923 | *--* | -- | hypothetical protein |
| AAur_0761 | 912 | 791533 | 792444 | *--* | -- | conserved hypothetical protein |
| AAur_0763 | 1062 | 793787 | 794848 | *--* | -- | conserved hypothetical protein |
| AAur_0767 | 459 | 799102 | 799560 | *--* | -- | putative appr-1-p processing enzyme domain with unknown function |
| AAur_0768 | 660 | 800240 | 799581 | *--* | -- | putative integral membrane protein |
| AAur_0774 | 627 | 803305 | 803931 | *--* | -- | conserved hypothetical protein |
| AAur_0777 | 438 | 807759 | 807322 | *--* | -- | putative STAS domain protein |
| AAur_0779 | 1263 | 810048 | 811310 | *--* | -- | putative beta-1,3-glucanase |
| AAur_0785 | 552 | 820319 | 819768 | *--* | -- | conserved hypothetical protein |
| AAur_0787 | 135 | 822565 | 822699 | *--* | -- | hypothetical protein |
| AAur_0793 | 381 | 827146 | 826766 | *--* | -- | putative glyoxalase family protein |
| AAur_0796 | 639 | 828896 | 829534 | *--* | -- | hypothetical protein |
| AAur_0806 | 300 | 839559 | 839260 | *--* | -- | hypothetical protein |
| AAur_0855 | 762 | 900705 | 901466 | *--* | -- | putative integral membrane protein |
| AAur_0856 | 735 | 901531 | 902265 | *--* | -- | putative integral membrane protein |
| AAur_0863 | 1077 | 910828 | 909752 | *--* | -- | putative NAD dependent epimerase/dehydratase family protein |
| AAur_0865 | 339 | 912299 | 911961 | *--* | -- | hypothetical protein |
| AAur_0866 | 486 | 912800 | 912315 | *--* | -- | hypothetical protein |
| AAur_0868 | 888 | 914752 | 913865 | *--* | -- | putative NAD dependent epimerase/dehydratase family protein |
| AAur_0871 | 405 | 917024 | 917428 | *--* | -- | putative NifU-like domain protein |
| AAur_0928 | 1584 | 987242 | 985659 | *nahA* | 3.2.1.52 | beta-N-acetylhexosaminidase |
| AAur_0934 | 114 | 992691 | 992578 | *--* | -- | hypothetical protein |
| AAur_0935 | 942 | 993629 | 992688 | *--* | -- | hypothetical protein |
| AAur_0937 | 936 | 995943 | 995008 | *--* | -- | hypothetical protein |
| AAur_0938 | 162 | 996155 | 996316 | *--* | -- | hypothetical protein |
| AAur_0939 | 285 | 996716 | 997000 | *--* | -- | conserved hypothetical protein |
| AAur_0941 | 114 | 997963 | 997850 | *--* | -- | hypothetical protein |
| AAur_0955 | 1359 | 1014109 | 1012751 | *--* | -- | putative prokaryotic protein of unknown function, DUF849-family |
| AAur_0957 | 372 | 1015271 | 1014900 | *--* | -- | hypothetical protein |
| AAur_0958 | 333 | 1016016 | 1016348 | *--* | -- | hypothetical protein |
| AAur_0959 | 687 | 1016562 | 1017248 | *--* | -- | hypothetical protein |
| AAur_0967 | 492 | 1024897 | 1025388 | *--* | -- | putative protein of unknown function (DUF805) |
| AAur_0970 | 216 | 1027587 | 1027802 | *--* | -- | hypothetical protein |
| AAur_0980 | 534 | 1036463 | 1036996 | *--* | -- | hypothetical protein |
| AAur_0982 | 660 | 1038422 | 1039081 | *--* | -- | hypothetical protein |
| AAur_0983 | 540 | 1039331 | 1039870 | *--* | -- | hypothetical protein |
| AAur_0984 | 492 | 1039978 | 1040469 | *--* | -- | hypothetical protein |
| AAur_0991 | 252 | 1049332 | 1049583 | *--* | -- | hypothetical protein |
| AAur_0994 | 1317 | 1051728 | 1053044 | *--* | -- | putative FemAB family protein |
| AAur_1010 | 474 | 1068507 | 1068034 | *--* | -- | hypothetical protein |
| AAur_1026 | 1008 | 1091122 | 1090115 | *--* | -- | hypothetical protein |
| AAur_1027 | 897 | 1092021 | 1091125 | *--* | -- | hypothetical protein |
| AAur_1043 | 96 | 1106900 | 1106805 | *--* | -- | hypothetical protein |
| AAur_1052 | 330 | 1118138 | 1117809 | *--* | -- | hypothetical protein |
| AAur_1066 | 3099 | 1135615 | 1138713 | *--* | -- | hypothetical protein |
| AAur_1067 | 1887 | 1138766 | 1140652 | *--* | -- | putative tetratricopeptide repeat family protein |
| AAur_1068 | 603 | 1140743 | 1141345 | *--* | -- | conserved hypothetical protein |
| AAur_1085 | 174 | 1157618 | 1157791 | *--* | -- | hypothetical protein |
| AAur_1087 | 684 | 1160075 | 1159392 | *bioD* | 6.3.3.3 | dethiobiotin synthase |
| AAur_1090 | 195 | 1162578 | 1162772 | *--* | -- | hypothetical protein |
| AAur_1096 | 204 | 1168459 | 1168662 | *--* | -- | hypothetical protein |
| AAur_1111 | 435 | 1184091 | 1184525 | *--* | -- | CBS domains protein |
| AAur_1122 | 1062 | 1193044 | 1194105 | *--* | -- | putative NAD dependent epimerase/dehydratase family protein |
| AAur_1132 | 711 | 1205337 | 1206047 | *--* | -- | putative integral membrane protein |
| AAur_1136 | 321 | 1210985 | 1210665 | *--* | -- | hypothetical protein |
| AAur_1142 | 717 | 1217174 | 1217890 | *--* | -- | putative cupin domain protein |
| AAur_1144 | 303 | 1219248 | 1218946 | *--* | -- | hypothetical protein |
| AAur_1153 | 783 | 1228355 | 1227573 | *--* | -- | hypothetical protein |
| AAur_1155 | 735 | 1229994 | 1229260 | *--* | -- | hypothetical protein |
| AAur_1156 | 819 | 1230881 | 1230063 | *--* | -- | hypothetical protein |
| AAur_1164 | 2829 | 1240447 | 1237619 | *--* | -- | conserved domain protein |
| AAur_1165 | 1908 | 1242338 | 1240431 | *--* | -- | putative protein of unknown function DUF262 family |
| AAur_1166 | 99 | 1244453 | 1244551 | *--* | -- | hypothetical protein |
| AAur_1171 | 648 | 1249751 | 1249104 | *--* | -- | hypothetical protein |
| AAur_1172 | 345 | 1250056 | 1249712 | *--* | -- | hypothetical protein |
| AAur_1173 | 384 | 1250112 | 1250495 | *--* | -- | hypothetical protein |
| AAur_1175 | 942 | 1252108 | 1251167 | *--* | -- | putative integral membrane protein |
| AAur_1181 | 567 | 1259979 | 1259413 | *--* | -- | putative Alpha/Beta hydrolase family of unknown function (DUF1234) |
| AAur_1185 | 1335 | 1265217 | 1263883 | *--* | -- | hypothetical protein |
| AAur_1190 | 1038 | 1267422 | 1268459 | *--* | -- | glyoxalase family protein |
| AAur_1195 | 960 | 1273771 | 1272812 | *--* | -- | putative SIS (Sugar ISomerase) domain protein |
| AAur_1200 | 303 | 1279666 | 1279364 | *--* | -- | hypothetical protein |
| AAur_1206 | 1254 | 1287081 | 1285828 | *--* | -- | putative FAD dependent oxidoreductase domain protein |
| AAur_1222 | 714 | 1306245 | 1305532 | *--* | -- | hypothetical protein |
| AAur_1233 | 1080 | 1316748 | 1315669 | *--* | -- | hypothetical protein |
| AAur_1241 | 963 | 1325793 | 1324831 | *--* | -- | putative glycosyl hydrolase, family 43 |
| AAur_1318 | 231 | 1406979 | 1407209 | *--* | -- | hypothetical protein |
| AAur_1319 | 696 | 1407250 | 1407945 | *--* | -- | putative pentapeptide repeats protein |
| AAur_1322 | 135 | 1411387 | 1411253 | *--* | -- | hypothetical protein |
| AAur_1324 | 567 | 1412452 | 1411886 | *--* | -- | hypothetical protein |
| AAur_1326 | 543 | 1414129 | 1413587 | *--* | -- | hypothetical protein |
| AAur_1361 | 432 | 1460980 | 1460549 | *--* | -- | hypothetical protein |
| AAur_1364 | 732 | 1462174 | 1462905 | *--* | -- | putative cation efflux family protein |
| AAur_1365 | 669 | 1462902 | 1463570 | *--* | -- | putative DSBA-like thioredoxin domain protein |
| AAur_1369 | 3138 | 1468457 | 1471594 | *--* | -- | hypothetical protein |
| AAur_1372 | 516 | 1474921 | 1475436 | *--* | -- | Tn554-related transposase C |
| AAur_1376 | 558 | 1479208 | 1478651 | *--* | -- | hypothetical protein |
| AAur_1377 | 234 | 1479761 | 1479528 | *--* | -- | putative membrane protein |
| AAur_1383 | 840 | 1487235 | 1486396 | *--* | -- | ISAau1, transposase orfB |
| AAur_1384 | 279 | 1487543 | 1487265 | *--* | -- | ISAau1, transposase orfA |
| AAur_1389 | 3072 | 1491810 | 1494881 | *--* | -- | Tn3 family transposase |
| AAur_1390 | 123 | 1494906 | 1495028 | *--* | -- | hypothetical protein |
| AAur_1391 | 405 | 1495025 | 1495429 | *--* | -- | putative glyoxalase family protein |
| AAur_1392 | 2103 | 1498152 | 1496050 | *--* | -- | phage integrase family domain protein |
| AAur_1393 | 1791 | 1499939 | 1498149 | *--* | -- | hypothetical protein |
| AAur_1394 | 1566 | 1501507 | 1499942 | *--* | -- | phage integrase family domain protein |
| AAur_1414 | 441 | 1530735 | 1530295 | *--* | -- | putative domain of unknown function DUF74 |
| AAur_1447 | 303 | 1585606 | 1585304 | *--* | -- | hypothetical protein |
| AAur_1451 | 834 | 1590973 | 1590140 | *--* | -- | Domain of unknown function (DUF323) protein |
| AAur_1461 | 279 | 1601631 | 1601909 | *--* | -- | ISAau1, transposase orfA |
| AAur_1462 | 840 | 1601939 | 1602778 | *--* | -- | ISAau1, transposase orfB |
| AAur_1465 | 4398 | 1609550 | 1605153 | *--* | -- | conserved hypothetical protein |
| AAur_1509 | 546 | 1655217 | 1654672 | *--* | -- | hypothetical protein |
| AAur_1517 | 582 | 1663182 | 1662601 | *--* | -- | hypothetical protein |
| AAur_1526 | 315 | 1672156 | 1671842 | *--* | -- | hypothetical protein |
| AAur_1550 | 744 | 1697371 | 1696628 | *--* | -- | putative ABC transporter efflux protein, DrrB family |
| AAur_1552 | 114 | 1698327 | 1698440 | *--* | -- | hypothetical protein |
| AAur_1560 | 450 | 1706172 | 1706621 | *--* | -- | putative protein of unknown function (DUF448) |
| AAur_1644 | 225 | 1795262 | 1795038 | *--* | -- | hypothetical protein |
| AAur_1645 | 129 | 1795451 | 1795323 | *--* | -- | hypothetical protein |
| AAur_1658 | 759 | 1813061 | 1812303 | *--* | -- | hypothetical protein |
| AAur_1737 | 486 | 1899834 | 1899349 | *--* | -- | hypothetical protein |
| AAur_1738 | 687 | 1900076 | 1900762 | *--* | -- | putative lipoprotein |
| AAur_1739 | 519 | 1901414 | 1900896 | *--* | -- | acetyltransferase, GNAT family protein |
| AAur_1742 | 1353 | 1904153 | 1902801 | *--* | -- | putative lipoprotein |
| AAur_1765 | 537 | 1932812 | 1933348 | *--* | -- | conserved hypothetical protein |
| AAur_1819 | 189 | 1988270 | 1988458 | *--* | -- | hypothetical protein |
| AAur_1847 | 138 | 2017984 | 2017847 | *--* | -- | hypothetical protein |
| AAur_1849 | 372 | 2020921 | 2021292 | *--* | -- | conserved hypothetical protein |
| AAur_1854 | 852 | 2027327 | 2026476 | *--* | -- | conserved hypothetical protein |
| AAur_1857 | 993 | 2030539 | 2029547 | *--* | -- | conserved hypothetical protein |
| AAur_1859 | 111 | 2032124 | 2032234 | *--* | -- | hypothetical protein |
| AAur_1860 | 276 | 2032231 | 2032506 | *--* | -- | hypothetical protein |
| AAur_1862 | 312 | 2034162 | 2034473 | *--* | -- | conserved hypothetical protein |
| AAur_1863 | 636 | 2034538 | 2035173 | *--* | -- | putative integral membrane protein (DUF1211) |
| AAur_1865 | 588 | 2038201 | 2037614 | *--* | -- | hypothetical protein |
| AAur_1868 | 180 | 2041463 | 2041284 | *--* | -- | hypothetical protein |
| AAur_1871 | 1089 | 2045735 | 2044647 | *--* | -- | conserved hypothetical protein |
| AAur_1875 | 96 | 2049856 | 2049951 | *--* | -- | hypothetical protein |
| AAur_1879 | 318 | 2053662 | 2053979 | *--* | -- | putative HspR protein |
| AAur_1883 | 732 | 2058317 | 2059048 | *--* | -- | conserved hypothetical protein |
| AAur_1896 | 369 | 2075044 | 2075412 | *--* | -- | death-on-curing protein |
| AAur_1898 | 1035 | 2076602 | 2077636 | *--* | 3.5.5.- | nitrilase |
| AAur_1899 | 1101 | 2077637 | 2078737 | *oxdA* | 4.99.1.5 | aldoxime dehydratase |
| AAur_1902 | 903 | 2083616 | 2082714 | *--* | -- | putative diacylglycerol kinase catalytic domain |
| AAur_1905 | 1296 | 2087663 | 2086368 | *--* | -- | ribulose bisphosphate carboxylase, large subunit, homolog |
| AAur_1906 | 1011 | 2088682 | 2087672 | *kdgT* | -- | 2-keto-3-deoxygluconate permease |
| AAur_1926 | 345 | 2117396 | 2117740 | *--* | -- | putative endoribonuclease L-PSP family |
| AAur_1933 | 714 | 2123823 | 2124536 | *--* | -- | conserved hypothetical protein |
| AAur_1934 | 219 | 2124529 | 2124747 | *--* | -- | conserved hypothetical protein |
| AAur_1935 | 1104 | 2124751 | 2125854 | *--* | -- | putative iron-sulfur cluster-binding protein, rieske family |
| AAur_1936 | 519 | 2126498 | 2125980 | *--* | -- | putative cupin domain protein |
| AAur_1938 | 798 | 2127303 | 2128100 | *--* | -- | putative homoserine dehydrogenase, NAD binding domain protein |
| AAur_1943 | 189 | 2134687 | 2134499 | *--* | -- | hypothetical protein |
| AAur_1997 | 435 | 2194259 | 2194693 | *--* | -- | putative ChaB family protein |
| AAur_2005 | 309 | 2203858 | 2203550 | *--* | -- | putative cold shock protein |
| AAur_2009 | 156 | 2208008 | 2207853 | *--* | -- | hypothetical protein |
| AAur_2013 | 465 | 2212591 | 2213055 | *--* | -- | conserved hypothetical protein |
| AAur_2012 | 405 | 2213456 | 2213052 | *--* | -- | putative endoribonuclease L-PSP family |
| AAur_2020 | 486 | 2219732 | 2219247 | *--* | -- | hypothetical protein |
| AAur_2027 | 1224 | 2224843 | 2223620 | *--* | -- | VWA domain containing CoxE-like protein family |
| AAur_2028 | 2304 | 2227128 | 2224825 | *--* | -- | conserved hypothetical protein |
| AAur_2030 | 1443 | 2229677 | 2228235 | *--* | -- | conserved hypothetical protein |
| AAur_2031 | 1299 | 2230972 | 2229674 | *--* | -- | putative SWIM zinc finger domain protein |
| AAur_2034 | 315 | 2234854 | 2234540 | *--* | -- | conserved hypothetical protein |
| AAur_2038 | 753 | 2240021 | 2239269 | *--* | -- | hypothetical protein |
| AAur_2056 | 948 | 2261668 | 2260721 | *--* | -- | putative transcriptional regulator, PaaX-like protein family |
| AAur_2099 | 603 | 2314142 | 2313540 | *--* | -- | conserved hypothetical protein |
| AAur_2123 | 594 | 2336108 | 2335515 | *--* | -- | conserved hypothetical protein |
| AAur_2148 | 117 | 2363109 | 2363225 | *--* | -- | hypothetical protein |
| AAur_2156 | 813 | 2372897 | 2373709 | *--* | -- | conserved hypothetical protein |
| AAur_2157 | 1248 | 2373706 | 2374953 | *--* | -- | conserved hypothetical protein |
| AAur_2176 | 174 | 2397105 | 2397278 | *--* | -- | conserved hypothetical protein |
| AAur_2256 | 1284 | 2485940 | 2484657 | *vanA* | 1.14.13.82 | vanillate O-demethylase oxygenase subunit |
| AAur_2326 | 1140 | 2561797 | 2562936 | *--* | -- | putative protein of unknown function (DUF1016) |
| AAur_2333 | 453 | 2568766 | 2569218 | *fabZ* | 4.2.1.- | (3R)-hydroxymyristoyl-[acyl carrier protein] dehydratase |
| AAur_2334 | 2160 | 2569215 | 2571374 | *--* | -- | acetyltransferase, GNAT family protein |
| AAur_2335 | 993 | 2571371 | 2572363 | *--* | -- | putative aminomethyltransferase (Glycine cleavage system Tprotein) |
| AAur_2337 | 558 | 2574758 | 2575315 | *--* | -- | hypothetical protein |
| AAur_2349 | 237 | 2586907 | 2586671 | *--* | -- | hypothetical protein |
| AAur_2397 | 240 | 2638090 | 2637851 | *--* | -- | hypothetical protein |
| AAur_2409 | 813 | 2645146 | 2645958 | *--* | -- | conserved hypothetical protein |
| AAur_2472 | 528 | 2715713 | 2715186 | *--* | -- | putative proteins of unknown function DUF177 |
| AAur_2525 | 318 | 2776279 | 2776596 | *--* | -- | hypothetical protein |
| AAur_2528 | 636 | 2778763 | 2779398 | *--* | -- | conserved hypothetical protein |
| AAur_2542 | 414 | 2792907 | 2792494 | *--* | -- | putative transcriptional regulator, MarR family |
| AAur_2546 | 903 | 2795969 | 2796871 | *--* | -- | conserved hypothetical protein |
| AAur_2565 | 687 | 2818215 | 2817529 | *--* | -- | putative integral membrane protein |
| AAur_2608 | 240 | 2860737 | 2860498 | *--* | -- | hypothetical protein |
| AAur_2666 | 1347 | 2924611 | 2925957 | *--* | -- | hypothetical protein |
| AAur_2667 | 900 | 2926008 | 2926907 | *--* | -- | putative lipoprotein |
| AAur_2668 | 528 | 2926992 | 2927519 | *--* | -- | putative lipoprotein |
| AAur_2671 | 1029 | 2931568 | 2932596 | *--* | -- | putative integral membrane protein |
| AAur_2672 | 2274 | 2932664 | 2934937 | *--* | -- | putative integral membrane protein |
| AAur_2690 | 183 | 2959182 | 2959000 | *--* | -- | hypothetical protein |
| AAur_2728 | 273 | 3003273 | 3003001 | *--* | -- | hypothetical protein |
| AAur_2767 | 948 | 3046746 | 3047693 | *--* | -- | putative TM2 domain family protein |
| AAur_2770 | 609 | 3052792 | 3052184 | *--* | -- | putative MarC family integral membrane protein |
| AAur_2805 | 906 | 3088218 | 3087313 | *--* | -- | hypothetical protein |
| AAur_2819 | 162 | 3101855 | 3101694 | *--* | -- | hypothetical protein |
| AAur_2821 | 1797 | 3104177 | 3102381 | *bgaC* | 3.2.1.23 | beta-galactosidase |
| AAur_2827 | 642 | 3109582 | 3110223 | *--* | -- | putative membrane protein (DUF1211 family) |
| AAur_2838 | 1704 | 3118992 | 3117289 | *--* | -- | putative ABC1 family domain protein |
| AAur_2841 | 528 | 3120013 | 3120540 | *--* | -- | hypothetical protein |
| AAur_2843 | 438 | 3121470 | 3121033 | *--* | -- | hypothetical protein |
| AAur_2844 | 153 | 3121609 | 3121457 | *--* | -- | hypothetical protein |
| AAur_2845 | 681 | 3121647 | 3122327 | *--* | -- | hypothetical protein |
| AAur_2846 | 747 | 3122454 | 3123200 | *--* | -- | hypothetical protein |
| AAur_2863 | 633 | 3140560 | 3139928 | *--* | -- | hypothetical protein |
| AAur_2872 | 330 | 3152131 | 3152460 | *--* | -- | hypothetical protein |
| AAur_2881 | 741 | 3162392 | 3161652 | *--* | -- | hypothetical protein |
| AAur_2915 | 1176 | 3199157 | 3200332 | *--* | -- | trypsin-like serine protease |
| AAur_2943 | 366 | 3219186 | 3218821 | *rplV* | -- | ribosomal protein L22 |
| AAur_2972 | 159 | 3254055 | 3253897 | *--* | -- | hypothetical protein |
| AAur_2990 | 321 | 3270283 | 3269963 | *--* | -- | hypothetical protein |
| AAur_2991 | 345 | 3270710 | 3270366 | *--* | -- | conserved hypothetical protein |
| AAur_3036 | 2145 | 3317101 | 3319245 | *--* | -- | putative phospholipase C |
| AAur_3046 | 678 | 3328051 | 3328728 | *--* | -- | putative transcriptional regulator, TetR family |
| AAur_3049 | 801 | 3329734 | 3330534 | *--* | -- | putative ABC transporter, permease protein |
| AAur_3077 | 558 | 3363028 | 3362471 | *--* | -- | conserved hypothetical protein |
| AAur_3078 | 123 | 3363192 | 3363314 | *--* | -- | hypothetical protein |
| AAur_3080 | 825 | 3365270 | 3364446 | *--* | -- | conserved hypothetical protein |
| AAur_3083 | 435 | 3367156 | 3367590 | *--* | -- | hypothetical protein |
| AAur_3094 | 537 | 3378445 | 3378981 | *--* | -- | hypothetical protein |
| AAur_3095 | 477 | 3378992 | 3379468 | *--* | -- | hypothetical protein |
| AAur_3108 | 828 | 3390901 | 3391728 | *--* | -- | hypothetical protein |
| AAur_3110 | 840 | 3395009 | 3394170 | *--* | -- | ISAau1, transposase orfB |
| AAur_3111 | 279 | 3395317 | 3395039 | *--* | -- | ISAau1, transposase orfA |
| AAur_3112 | 279 | 3395599 | 3395877 | *--* | -- | ISAau1, transposase orfA |
| AAur_3113 | 840 | 3395907 | 3396746 | *--* | -- | ISAau1, transposase orfB |
| AAur_3116 | 690 | 3400968 | 3400279 | *--* | -- | hypothetical protein |
| AAur_3117 | 660 | 3400988 | 3401647 | *--* | -- | hypothetical protein |
| AAur_3133 | 240 | 3425429 | 3425668 | *--* | -- | hypothetical protein |
| AAur_3137 | 1011 | 3434545 | 3433535 | *--* | -- | putative cobalt-zinc-cadmium efflux permease |
| AAur_3138 | 480 | 3435021 | 3434542 | *--* | -- | putative lipoprotein |
| AAur_3141 | 237 | 3437989 | 3437753 | *--* | -- | hypothetical protein |
| AAur_3145 | 213 | 3449104 | 3448892 | *--* | -- | conserved hypothetical protein |
| AAur_3146 | 366 | 3449542 | 3449177 | *--* | -- | putative cupin domain protein |
| AAur_3147 | 471 | 3450061 | 3449591 | *--* | -- | conserved hypothetical protein |
| AAur_3152 | 840 | 3453854 | 3453015 | *--* | -- | ISAau1, transposase orfB |
| AAur_3153 | 279 | 3454162 | 3453884 | *--* | -- | ISAau1, transposase orfA |
| AAur_3158 | 549 | 3458799 | 3459347 | *--* | -- | putative uncharacterized DUF218 domain protein |
| AAur_3163 | 1023 | 3467827 | 3466805 | *--* | -- | hypothetical protein |
| AAur_3165 | 1227 | 3469840 | 3468614 | *--* | -- | putative integral membrane protein |
| AAur_3166 | 1287 | 3471136 | 3469850 | *--* | -- | putative integral membrane protein |
| AAur_3167 | 1098 | 3473128 | 3472031 | *--* | -- | hypothetical protein |
| AAur_3299 | 840 | 3623156 | 3623995 | *--* | -- | ISAau1, transposase orfB |
| AAur_3298 | 279 | 3622848 | 3623126 | *--* | -- | ISAau1, transposase orfA |
| AAur_3177 | 123 | 3485430 | 3485552 | *--* | -- | hypothetical protein |
| AAur_3182 | 513 | 3490114 | 3490626 | *--* | -- | conserved hypothetical protein |
| AAur_3183 | 957 | 3490661 | 3491617 | *--* | -- | putative FHA domain protein |
| AAur_3185 | 1017 | 3494290 | 3495306 | *--* | -- | hypothetical protein |
| AAur_3188 | 984 | 3499066 | 3500049 | *--* | -- | hypothetical protein |
| AAur_3189 | 2292 | 3500046 | 3502337 | *--* | -- | hypothetical protein |
| AAur_3190 | 333 | 3503084 | 3502752 | *--* | -- | hypothetical protein |
| AAur_3191 | 180 | 3503961 | 3504140 | *--* | -- | hypothetical protein |
| AAur_3192 | 648 | 3504195 | 3504842 | *--* | -- | hypothetical protein |
| AAur_3193 | 891 | 3505814 | 3504924 | *--* | -- | hypothetical protein |
| AAur_3194 | 423 | 3506266 | 3505844 | *--* | -- | hypothetical protein |
| AAur_3198 | 444 | 3508807 | 3509250 | *--* | -- | hypothetical protein |
| AAur_3199 | 420 | 3509868 | 3509449 | *--* | -- | acetyltransferase, GNAT family protein |
| AAur_3201 | 582 | 3511323 | 3511904 | *--* | -- | putative protein of unknown function (DUF1349) |
| AAur_3205 | 1464 | 3516309 | 3514846 | *--* | -- | putative uncharacterized ACR, YdiU/UPF0061 family protein |
| AAur_3206 | 105 | 3516719 | 3516615 | *--* | -- | hypothetical protein |
| AAur_3207 | 1335 | 3516879 | 3518213 | *--* | -- | putative integral membrane protein |
| AAur_3208 | 972 | 3519313 | 3520284 | *--* | -- | conserved hypothetical protein |
| AAur_3211 | 177 | 3525950 | 3525774 | *--* | -- | hypothetical protein |
| AAur_3215 | 1527 | 3529583 | 3531109 | *--* | -- | putative lipoprotein |
| AAur_3219 | 165 | 3538377 | 3538213 | *--* | -- | hypothetical protein |
| AAur_3220 | 1551 | 3538545 | 3540095 | *--* | -- | putative lipoprotein |
| AAur_3231 | 780 | 3549911 | 3549132 | *--* | -- | hypothetical protein |
| AAur_3239 | 174 | 3560692 | 3560865 | *--* | -- | hypothetical protein |
| AAur_3268 | 537 | 3592889 | 3593425 | *--* | -- | hypothetical protein |
| AAur_3281 | 603 | 3602123 | 3602725 | *--* | -- | hypothetical protein |
| AAur_3280 | 1476 | 3604181 | 3602706 | *--* | -- | putative GTPase of unknown function |
| AAur_3282 | 1875 | 3606052 | 3604178 | *--* | -- | conserved hypothetical protein |
| AAur_3283 | 1557 | 3606225 | 3607781 | *--* | -- | hypothetical protein |
| AAur_1740 | 840 | 1902220 | 1901381 | *--* | -- | ISAau1, transposase orfB |
| AAur_3300 | 432 | 3624551 | 3624120 | *--* | -- | hypothetical protein |
| AAur_3373 | 1071 | 3711487 | 3710417 | *--* | -- | putative 3-oxoacyl-(acyl-carrier-protein) synthase III |
| AAur_3376 | 969 | 3714581 | 3713613 | *--* | -- | putative A-factor biosynthesis repeat family protein |
| AAur_3404 | 483 | 3750745 | 3751227 | *--* | -- | conserved hypothetical protein |
| AAur_3411 | 309 | 3758057 | 3757749 | *--* | -- | conserved hypothetical protein |
| AAur_3443 | 450 | 3793254 | 3793703 | *--* | -- | putative rhodanese-like domain protein |
| AAur_3455 | 921 | 3807699 | 3808619 | *--* | -- | conserved hypothetical protein |
| AAur_3456 | 645 | 3808798 | 3809442 | *--* | -- | hypothetical protein |
| AAur_3457 | 156 | 3809764 | 3809609 | *--* | -- | hypothetical protein |
| AAur_3459 | 438 | 3811353 | 3810916 | *--* | -- | hypothetical protein |
| AAur_3461 | 132 | 3812159 | 3812028 | *--* | -- | hypothetical protein |
| AAur_3462 | 504 | 3812256 | 3812759 | *--* | -- | conserved hypothetical protein |
| AAur_3465 | 678 | 3817335 | 3816658 | *--* | -- | putative acyl carrier protein phosphodiesterase |
| AAur_3471 | 288 | 3821963 | 3821676 | *--* | -- | conserved hypothetical protein |
| AAur_3472 | 171 | 3822433 | 3822263 | *--* | -- | hypothetical protein |
| AAur_3479 | 1020 | 3827794 | 3828813 | *--* | -- | conserved hypothetical protein |
| AAur_3480 | 903 | 3828823 | 3829725 | *--* | -- | conserved hypothetical protein |
| AAur_3481 | 945 | 3829919 | 3830863 | *--* | -- | putative integral membrane protein |
| AAur_3483 | 222 | 3832858 | 3833079 | *--* | -- | hypothetical protein |
| AAur_3484 | 699 | 3833859 | 3833161 | *--* | -- | putative integral membrane protein |
| AAur_3488 | 1242 | 3838242 | 3837001 | *--* | -- | hypothetical protein |
| AAur_3489 | 816 | 3839045 | 3838230 | *--* | -- | conserved hypothetical protein |
| AAur_3490 | 177 | 3839306 | 3839130 | *--* | -- | hypothetical protein |
| AAur_3492 | 1224 | 3840851 | 3839628 | *--* | -- | hypothetical protein |
| AAur_3494 | 1023 | 3843159 | 3842137 | *--* | -- | hypothetical protein |
| AAur_3495 | 1074 | 3843391 | 3844464 | *--* | -- | hypothetical protein |
| AAur_3496 | 444 | 3845402 | 3844959 | *--* | -- | hypothetical protein |
| AAur_3502 | 699 | 3851403 | 3850705 | *--* | -- | conserved hypothetical protein |
| AAur_3504 | 531 | 3853484 | 3854014 | *--* | -- | hypothetical protein |
| AAur_3505 | 1437 | 3856584 | 3855148 | *--* | -- | hypothetical protein |
| AAur_3517 | 561 | 3868829 | 3869389 | *--* | -- | putative lipoprotein |
| AAur_3532 | 618 | 3883736 | 3884353 | *--* | -- | Pyridoxamine 5'-phosphate oxidase family protein |
| AAur_3556 | 414 | 3907107 | 3907520 | *--* | -- | hypothetical protein |
| AAur_3564 | 717 | 3912366 | 3913082 | *--* | -- | hypothetical protein |
| AAur_3566 | 840 | 3915244 | 3914405 | *--* | -- | putative membrane protein (DUF990 family) |
| AAur_3567 | 795 | 3916031 | 3915237 | *--* | -- | putative membrane protein (DUF990 family) |
| AAur_3568 | 1062 | 3916051 | 3917112 | *--* | -- | hypothetical protein |
| AAur_3572 | 1092 | 3922900 | 3923991 | *--* | -- | hypothetical protein |
| AAur_3577 | 423 | 3928411 | 3928833 | *--* | -- | hypothetical protein |
| AAur_3595 | 558 | 3943681 | 3944238 | *--* | -- | hypothetical protein |
| AAur_3601 | 507 | 3950569 | 3951075 | *--* | -- | putative integral membrane protein |
| AAur_3602 | 459 | 3951548 | 3951090 | *--* | -- | conserved hypothetical protein |
| AAur_3603 | 354 | 3954858 | 3954505 | *--* | -- | putative integral membrane protein |
| AAur_3607 | 306 | 3958721 | 3958416 | *--* | -- | hypothetical protein |
| AAur_3608 | 693 | 3958793 | 3959485 | *--* | -- | hypothetical protein |
| AAur_3609 | 1284 | 3959529 | 3960812 | *--* | -- | putative phage tail sheath family protein |
| AAur_3610 | 480 | 3960814 | 3961293 | *--* | -- | conserved hypothetical protein |
| AAur_3611 | 1449 | 3961533 | 3962981 | *--* | -- | hypothetical protein |
| AAur_3612 | 1521 | 3962978 | 3964498 | *--* | -- | putative phage-related protein |
| AAur_3613 | 339 | 3964537 | 3964875 | *--* | -- | hypothetical protein |
| AAur_3614 | 435 | 3964889 | 3965323 | *--* | -- | putative phage-related protein |
| AAur_3615 | 2628 | 3965320 | 3967947 | *--* | -- | putative phage-related protein |
| AAur_3616 | 534 | 3967940 | 3968473 | *--* | -- | putative phage tail domain protein |
| AAur_3619 | 714 | 3971307 | 3972020 | *--* | -- | hypothetical protein |
| AAur_3620 | 1326 | 3972017 | 3973342 | *--* | -- | putative Bacterial surface protein containing Ig-like domains |
| AAur_3621 | 813 | 3974158 | 3973346 | *--* | -- | putative integral membrane protein |
| AAur_3633 | 831 | 3985131 | 3985961 | *--* | -- | putative integral membrane protein |
| AAur_3645 | 357 | 3995360 | 3995716 | *--* | -- | hypothetical protein |
| AAur_3649 | 318 | 4000746 | 4000429 | *--* | -- | conserved hypothetical protein |
| AAur_3657 | 144 | 4009298 | 4009155 | *--* | -- | hypothetical protein |
| AAur_3669 | 2136 | 4021929 | 4024064 | *--* | -- | putative penicillin amidase |
| AAur_3696 | 567 | 4050337 | 4050903 | *--* | -- | acetyltransferase, GNAT family protein |
| AAur_3702 | 171 | 4057485 | 4057655 | *--* | -- | hypothetical protein |
| AAur_3703 | 210 | 4057999 | 4057790 | *--* | -- | hypothetical protein |
| AAur_3718 | 546 | 4075925 | 4076470 | *--* | -- | putative Helix-turn-helix domain protein |
| AAur_3719 | 336 | 4076536 | 4076871 | *--* | -- | conserved hypothetical protein |
| AAur_3726 | 528 | 4084498 | 4085025 | *--* | -- | acetyltransferase, GNAT family protein |
| AAur_3728 | 162 | 4086479 | 4086318 | *--* | -- | hypothetical protein |
| AAur_3778 | 1641 | 4149268 | 4147628 | *--* | -- | hypothetical protein |
| AAur_3782 | 1206 | 4153487 | 4152282 | *--* | -- | PQQ (pyrrolo-quinoline quinone) enzyme repeat domain protein |
| AAur_3784 | 1254 | 4156137 | 4154884 | *--* | -- | hypothetical protein |
| AAur_3786 | 156 | 4157314 | 4157159 | *--* | -- | hypothetical protein |
| AAur_3796 | 870 | 4168324 | 4167455 | *--* | -- | putative amidohydrolase family protein |
| AAur_3800 | 1308 | 4170866 | 4169559 | *--* | -- | putative alpha-L-fucosidase |
| AAur_3805 | 123 | 4175112 | 4175234 | *--* | -- | hypothetical protein |
| AAur_3813 | 141 | 4182738 | 4182598 | *--* | -- | hypothetical protein |
| AAur_3849 | 366 | 4220211 | 4220576 | *--* | -- | conserved hypothetical protein |
| AAur_3864 | 666 | 4239333 | 4239998 | *--* | -- | conserved hypothetical protein |
| AAur_3874 | 96 | 4250242 | 4250337 | *--* | -- | hypothetical protein |
| AAur_3882 | 492 | 4259398 | 4258907 | *--* | -- | hypothetical protein |
| AAur_3883 | 1098 | 4260524 | 4259427 | *--* | -- | hypothetical protein |
| AAur_3890 | 720 | 4267257 | 4266538 | *--* | -- | PAP2 superfamily domain protein |
| AAur_3907 | 177 | 4284975 | 4284799 | *--* | -- | hypothetical protein |
| AAur_3910 | 852 | 4287131 | 4287982 | *--* | -- | hypothetical protein |
| AAur_3919 | 651 | 4295822 | 4296472 | *--* | -- | putative esterase/lipase |
| AAur_3924 | 564 | 4299699 | 4299136 | *--* | -- | putative isochorismatase family protein |
| AAur_3928 | 177 | 4301894 | 4301718 | *--* | -- | hypothetical protein |
| AAur_3930 | 177 | 4304221 | 4304045 | *--* | -- | hypothetical protein |
| AAur_3936 | 114 | 4306757 | 4306644 | *--* | -- | hypothetical protein |
| AAur_3938 | 612 | 4307508 | 4308119 | *--* | -- | hypothetical protein |
| AAur_3939 | 351 | 4308116 | 4308466 | *--* | -- | hypothetical protein |
| AAur_3947 | 252 | 4317868 | 4317617 | *--* | -- | hypothetical protein |
| AAur_3953 | 1224 | 4323031 | 4324254 | *nylB* | 3.5.1.46 | 6-aminohexanoate-dimer hydrolase |
| AAur_3955 | 924 | 4325948 | 4326871 | *--* | -- | hydrolase, alpha/beta fold family domain protein |
| AAur_3957 | 285 | 4327841 | 4327557 | *--* | -- | hypothetical protein |
| AAur_3970 | 849 | 4340813 | 4339965 | *--* | -- | putative conserved domain protein |
| AAur_3971 | 1401 | 4341440 | 4342840 | *--* | 4.1.1.15 | glutamate decarboxylase |
| AAur_3972 | 870 | 4342862 | 4343731 | *--* | -- | putative glutamine amidotransferase domain protein |
| AAur_3976 | 459 | 4347437 | 4346979 | *--* | -- | putative lactoylglutathione lyase |
| AAur_3983 | 693 | 4354659 | 4353967 | *--* | -- | hypothetical protein |
| AAur_3984 | 636 | 4355401 | 4354766 | *--* | -- | hypothetical protein |
| AAur_3986 | 1257 | 4357640 | 4356384 | *--* | -- | putative integral membrane protein |
| AAur_3987 | 399 | 4357938 | 4358336 | *--* | -- | putative integral membrane protein |
| AAur_3994 | 402 | 4366127 | 4365726 | *--* | -- | hypothetical protein |
| AAur_4024 | 885 | 4398984 | 4398100 | *--* | -- | putative transcriptional regulator, PaaX-like protein family |
| AAur_4025 | 381 | 4399518 | 4399138 | *--* | -- | putative integral membrane protein |
| AAur_4027 | 396 | 4400660 | 4400265 | *--* | -- | putative thioesterase family domain protein |
| AAur_4032 | 483 | 4407548 | 4407066 | *--* | -- | putative cupin domain protein |
| AAur_4061 | 1659 | 4436065 | 4434407 | *--* | -- | N-acyl-D-amino acid deacylase family protein |
| AAur_4072 | 549 | 4449335 | 4448787 | *--* | -- | putative acetyltransferase |
| AAur_4074 | 1338 | 4450456 | 4451793 | *--* | -- | putative protein of unknown function (DUF1343) |
| AAur_4076 | 1011 | 4454912 | 4453902 | *--* | -- | putative integral membrane protein with DUF218 domain |
| AAur_4077 | 381 | 4455315 | 4454935 | *--* | -- | conserved hypothetical protein |
| AAur_4083 | 678 | 4462209 | 4462886 | *--* | -- | putative lipoprotein |
| AAur_4086 | 654 | 4466629 | 4465976 | *--* | -- | conserved hypothetical protein |
| AAur_4089 | 1623 | 4469484 | 4467862 | *nahA* | 3.2.1.52 | beta-N-acetylhexosaminidase |
| AAur_4103 | 576 | 4481164 | 4481739 | *--* | -- | conserved hypothetical protein |
| AAur_4106 | 564 | 4482779 | 4483342 | *--* | -- | conserved hypothetical protein |
| AAur_4114 | 1227 | 4493702 | 4492476 | *--* | -- | putative glycosyl transferase, group 1 family protein |
| AAur_4115 | 246 | 4494030 | 4493785 | *--* | -- | hypothetical protein |
| AAur_4117 | 1335 | 4495463 | 4496797 | *--* | -- | putative regulatory protein, araC family signature |
| AAur_4119 | 864 | 4498178 | 4499041 | *--* | -- | conserved hypothetical protein |
| AAur_4126 | 729 | 4507474 | 4508202 | *--* | -- | hypothetical protein |
| AAur_4127 | 552 | 4509816 | 4509265 | *--* | -- | putative Tat pathway signal sequence domain protein |
| AAur_4142 | 1851 | 4526431 | 4524581 | *--* | -- | putative Tat pathway signal sequence domain protein |
| AAur_4148 | 978 | 4532628 | 4533605 | *--* | -- | putative hydroxymethylglutaryl-CoA lyase |
| AAur_4152 | 294 | 4536398 | 4536105 | *--* | -- | hypothetical protein |
| AAur_4155 | 756 | 4539101 | 4538346 | *--* | -- | conserved hypothetical protein |
| AAur_4159 | 1533 | 4540825 | 4542357 | *--* | -- | putative integral membrane protein |
| AAur_4166 | 339 | 4545783 | 4546121 | *--* | -- | conserved hypothetical protein |
| AAur_4168 | 147 | 4546417 | 4546563 | *--* | -- | hypothetical protein |
| AAur_4183 | 3849 | 4568180 | 4564332 | *--* | -- | putative glycosyl hydrolases family 31 |
| AAur_4200 | 135 | 4586068 | 4585934 | *--* | -- | hypothetical protein |
| AAur_4201 | 138 | 4586316 | 4586179 | *--* | -- | hypothetical protein |
| AAur_4202 | 93 | 4588250 | 4588158 | *--* | -- | hypothetical protein |
| AAur_3187 | 2937 | 3495977 | 3498913 | *--* | -- | putative membrane protein |
| AAur_1163 | 1077 | 1237604 | 1236528 | *--* | -- | hypothetical protein |
| AAur_4209 | 138 | 4597221 | 4597084 | *rpmH* | -- | ribosomal protein L34 |
| AAur_3173 | 840 | 3484083 | 3483244 | *--* | -- | ISAau1, transposase orfB |
| AAur_0710 | 303 | 734361 | 734663 | *--* | -- | putative Helix-turn-helix domain protein |
| AAur_1162 | 291 | 1236227 | 1235937 | *--* | -- | hypothetical protein |
| AAur_1161 | 405 | 1235838 | 1235434 | *--* | -- | hypothetical protein |
| AAur_3491 | 249 | 3839582 | 3839334 | *--* | -- | hypothetical protein |
| AAur_3485 | 957 | 3834816 | 3833860 | *--* | -- | putative membrane protein |
| AAur_3175 | 306 | 3484418 | 3484113 | *--* | -- | ISAau1, transposase orfA |
| AAur_1741 | 279 | 1902528 | 1902250 | *--* | -- | ISAau1, transposase orfA |
