# Supplementary material for: Secrets of Soil Survival Revealed by the Genome Sequence of Arthrobacter aurescens TC1
Source: PLoS Genet. 2006 Dec 22;2(12):e214. doi: 10.1371/journal.pgen.0020214 (PMC1713258; doi:10.1371/journal.pgen.0020214)
Supplement: Supplementary file 9 [file pgen.0020214.st006.doc]

**Supplemental Table 6. Genes in the *A. aurescens* TC1 genome involved in stress survival.**

| **Gene** | **Predicted Protein Product** | | | **Gene Symbol** | **EC#** | | **PFAM/**  **TIGRFAM** | | **Evidence Name** | |
| --- | --- | --- | --- | --- | --- | --- | --- | --- | --- | --- |
| **Genes involved in survival to internal oxidative stress** | | | | | | | | | | |
| **- Amine oxidases:** | | | |  |  | |  | |  | |
| AAur_0043 | putrescine oxidase | | | *puo* | 1.4.3.10 | | PF01266 | | FAD dependent oxidoreductase | |
|  |  | | |  |  | | PF01593 | | amine oxidase, flavin-containing | |
| AAur_0579 | amine oxidase (copper-containing) | | | *--* | 1.4.3.6 | | PF02727 | | Copper amine oxidase, N2 domain | |
|  |  | | |  |  | | PF02728 | | Copper amine oxidase, N3 domain | |
|  |  | | |  |  | | PF01179 | | Copper amine oxidase, enzyme domain | |
| AAur_1378 | amine oxidase (copper-containing) | | | *--* | 1.4.3.6 | | PF02727 | | Copper amine oxidase, N2 domain | |
|  |  | | |  |  | | PF02728 | | Copper amine oxidase, N3 domain | |
|  |  | | |  |  | | PF01179 | | Copper amine oxidase, enzyme domain | |
| AAur_2533 | L-aspartate oxidase | | | *nadB* | 1.4.3.16 | | TIGR00551 | | L-aspartate oxidase | |
|  |  | | |  |  | | PF07992 | | Pyridine nucleotide-disulphide oxidoreductase | |
|  |  | | |  |  | | PF00890 | | FAD binding domain | |
|  |  | | |  |  | | PF01266 | | FAD dependent oxidoreductase | |
|  |  | | |  |  | | PF02910 | | Fumarate reductase/succinate dehydrogenase flavoprotein C-terminal domain | |
| AAur_2764 | glycine oxidase ThiO | | | *thiO* | 1.4.3.19 | | PF01266 | | FAD dependent oxidoreductase | |
|  |  | | |  |  | | TIGR02352 | | glycine oxidase ThiO | |
| AAur_pTC20192 | amine oxidase (copper-containing) | | | *--* | 1.4.3.6 | | PF02727 | | Copper amine oxidase, N2 domain | |
|  |  | | |  |  | | PF02728 | | Copper amine oxidase, N3 domain | |
|  |  | | |  |  | | PF01179 | | Copper amine oxidase, enzyme domain | |
| AAur_pTC20082 | amine oxidase, copper-containing | | | *--* | 1.4.3.6 | | PF02727 | | Copper amine oxidase, N2 domain | |
|  |  | | |  |  | | PF02728 | | Copper amine oxidase, N3 domain | |
|  |  | | |  |  | | PF01179 | | Copper amine oxidase, enzyme domain | |
| AAur_0171 | mitomycin radical oxidase | | | *mcrA* | 1.5.3.- | | PF01565 | | FAD binding domain | |
| AAur_0597 | fructosyl-amino acid oxidase | | | *--* | 1.5.3.- | | PF01266 | | FAD dependent oxidoreductase | |
| AAur_1202 | sarcosine oxidase, monomeric form | | | *soxA* | 1.5.3.1 | | PF01266 | | FAD dependent oxidoreductase | |
|  |  | | |  |  | | PF07992 | | Pyridine nucleotide-disulphide oxidoreductase | |
| AAur_3826 | Sarcosine oxidase beta subunit | | | *soxB* | 1.5.3.1 | | TIGR01373 | | sarcosine oxidase, beta subunit family | |
|  |  | | |  |  | | PF01266 | | FAD dependent oxidoreductase | |
| AAur_3827 | Sarcosine oxidase delta subunit | | | *--* | 1.5.3.1 | | PF04267 | | Sarcosine oxidase, delta subunit family | |
|  |  | | |  |  | | TIGR01374 | | sarcosine oxidase, delta subunit family | |
| AAur_3828 | sarcosine oxidase alpha subunit | | | *--* | 1.5.3.1 | | TIGR01372 | | sarcosine oxidase, alpha subunit family | |
|  |  | | |  |  | | PF07992 | | Pyridine nucleotide-disulphide oxidoreductase | |
|  |  | | |  |  | | PF01266 | | FAD dependent oxidoreductase | |
|  |  | | |  |  | | PF01571 | | Glycine cleavage T-protein (transferase) | |
| AAur_3829 | Sarcosine oxidase gamma subunit | | | *soxG* | 1.5.3.1 | | PF04268 | | Sarcosine oxidase, gamma subunit family | |
| - **Metabolism of reactive oxygen species:** | | | | | | |  | |  | |
| AAur_0634 | catalase, Mn-containing | | | *--* | 1.11.1.6 | | PF05067 | | Manganese containing catalase | |
| AAur_1251 | organic hydroperoxide resistance protein | | | *ohr* | -- | | PF02566 | | OsmC-like protein | |
| AAur_1864 | catalase | | | *--* | 1.11.1.6 | | PF00199 | | catalase | |
|  |  | | |  |  | | PF01965 | | DJ-1/PfpI family | |
|  |  | | |  |  | | PF06628 | | Catalase-related | |
| AAur_2025 | uncharacterized peroxidase-related enzyme | | | *--* | -- | | TIGR01926 | | uncharacterized peroxidase-related enzyme | |
| AAur_2087 | superoxide dismutase | | | *sodA* | 1.15.1.1 | | PF02777 | | superoxide dismutase (Mn/Fe family), C-terminal domain | |
|  |  | | |  |  | | PF00081 | | Iron/manganese superoxide dismutases, alpha-hairpin domain | |
| AAur_3059 | catalase | | | *--* | 1.11.1.6 | | PF00199 | | catalase | |
| AAur_3550 | redox-sensitive transcriptional activator SoxR | | | *soxR* | -- | | PF00376 | | transcriptional regulator, MerR family | |
|  |  | | |  |  | | TIGR01950 | | redox-sensitive transcriptional activator SoxR | |
| **Genes involved in protection against osmotic stress** | | | | | | | | |  | |
| **- Biosynthesis and catabolism of trehalose:** | | | | | | | | |  | |
| AAur_0306 | putative trehalose/maltose hydrolase (possible phosphorylase) | | | *--* | -- | | PF03633 | | Glycosyl hydrolase family 65, C-terminal domain | |
|  |  | | |  |  | | PF03636 | | Glycosyl hydrolase family 65, N-terminal domain | |
|  |  | | |  |  | | PF03632 | | Glycosyl hydrolase family 65 cent catalytic dom. | |
| AAur_0909 | trehalose synthase | | | *treS* | 5.4.99.16 | | TIGR02456 | | trehalose synthase | |
|  |  | | |  |  | | PF00128 | | Alpha amylase, catalytic domain | |
| AAur_0930 | trehalose-phosphatase | | | *otsB* | 3.1.3.12 | | PF02358 | | Trehalose-phosphatase | |
|  |  | | |  |  | | TIGR00685 | | trehalose-phosphatase | |
|  |  | | |  |  | | TIGR01484 | | HAD-superfamily hydrolase, subfamily IIB | |
| AAur_0931 | trehalose-6-phosphate synthase | | | *otsB* | 2.4.1.15 | | PF00982 | | Trehalose-6-phosphate synthase domain | |
|  |  | | |  |  | | PF00534 | | glycosyl transferase, group 1 family protein | |
| AAur_2895 | malto-oligosyltrehalose trehalohydrolase | | | *treZ* | 3.2.1.141 | | PF02922 | | Isoamylase N-terminal domain | |
|  |  | | |  |  | | PF00128 | | Alpha amylase, catalytic domain | |
|  |  | | |  |  | | TIGR02402 | | malto-oligosyltrehalose trehalohydrolase | |
| AAur_2896 | malto-oligosyltrehalose synthase | | | *treY* | 5.4.99.15 | | PF00128 | | Alpha amylase, catalytic domain | |
|  |  | | |  |  | | TIGR02401 | | malto-oligosyltrehalose synthase | |
| AAur_3722 | trehalose-phosphatase | | | *otsB* | 3.1.3.12 | | PF02358 | | Trehalose-phosphatase | |
|  | |  |  | | | TIGR00685 | | Trehalose-phosphatase | |  |
|  |  | | |  |  | | PF00723 | | Glycosyl hydrolase, family 15 | |
| AAur_2137 | glycogen synthase | | | *glgA* | -- | | TIGR02149 | | glycogen synthase, Corynebacterium family | |
|  |  | | |  |  | | PF00534 | | glycosyl transferase, group 1 family protein | |
| AAur_2897 | glycogen debranching enzyme | | | *glgX* | 3.2.1.- | | PF02922 | | Isoamylase N-terminal domain | |
|  |  | | |  |  | | PF00128 | | Alpha amylase, catalytic domain | |
|  |  | | |  |  | | TIGR02100 | | glycogen debranching enzyme GlgX | |
| AAur_0691 | putative glycogen debranching enzyme family protein | | | *--* | -- | | PF06202 | | Amylo-alpha-1,6-glucosidase | |
| AAur_0904 | glycogen debranching enzyme GlgX | | | *glgX* | 3.2.1.- | | PF02922 | | Isoamylase N-terminal domain | |
|  |  | | |  |  | | PF00128 | | Alpha amylase, catalytic domain | |
|  |  | | |  |  | | TIGR02100 | | glycogen debranching enzyme GlgX | |
| **- Biosynthesis and accumulation of glycine betaine:** | | | | | | | | | | |
| AAur_0280 | proline-betaine transporter | | | *prop* | -- | | PF07690 | | transporter, major facilitator family | |
|  |  | | |  |  | | PF00083 | | transporter, major facilitator superfamily | |
| AAur_0512 | choline oxidase | | | *--* | 1.1.3.17 | | PF00732 | | GMC oxidoreductase | |
|  |  | | |  |  | | PF05199 | | GMC oxidoreductase | |
| AAur_0513 | betaine aldehyde dehydrogenase | | | *betB* | 1.2.1.8 | | PF00171 | | aldehyde dehydrogenase (NAD) family protein | |
| AAur_0516 | HTH-type transcriptional regulator | | | *betI* | -- | | PF00440 | | transcriptional regulator, TetR family | |
| AAur_0644 | putative glycine betaine/carnitine/ choline ABC transporter | | | *--* | -- | | PF00528 | | ABC transporter, permease protein | |
| AAur_0647 | putative substrate binding domain glycine/betaine ABC transport sys | | | *--* | -- | | PF04069 | | ABC transporter, quaternary amine uptake transporter (QAT) family, substrate-binding | |
| AAur_2814 | glycine betaine/choline-binding protein ABC-type transport system | | | *proX* | -- | | PF04069 | | ABC transporter, quaternary amine uptake transporter (QAT) family, subst.-binding protein | |
| AAur_2817 | ABC-type proline/glycine betaine transport systems, ATPase compon | | | *proV* | -- | | PF00005 | | ABC transporter, ATP-binding protein | |
| AAur_3606 | choline dehydrogenase | | | *beta* | 1.1.99.1 | | PF00732 | | GMC oxidoreductase | |
|  |  | | |  |  | | PF05199 | | GMC oxidoreductase | |
| **- Other Genes:** | | | |  |  | |  | |  | |
| AAur_2559 | aquaporin Z | | | *aqpZ* | -- | | PF00230 | | transporter, major intrinsic protein (MIP) family | |
| **Cupin genes: related to stress, Mn accumulation and morphogenesis** | | | | | | | | | | |
| AAur_0331 | gentisate 1,2-dioxygenase | | | *gtdA* | 1.13.11.4 | | PF07883 | | Cupin Domain (2 domains) | |
| AAur_0378 | cupin superfamily protein, putative | | | *--* | -- | | PF08007 | | Cupin Superfamily Protein | |
| AAur_0978 | Put. Helix-turn-helix domain prot | | | *--* | -- | | PF07883 | | Cupin domain | |
|  |  | | |  |  | | PF01381 | | Helix-turn-helix | |
| AAur_1055 | Put. Helix-turn-helix domain prot | | | *--* | -- | | PF07883 | | Cupin domain | |
|  |  | | |  |  | | PF01381 | | Helix-turn-helix | |
| AAur_1082 | Put. Helix-turn-helix domain prot | | | *--* | -- | | PF07883 | | Cupin domain | |
|  |  | | |  |  | | PF01381 | | Helix-turn-helix | |
| AAur_1142 | cupin domain protein, putative | | | *--* | -- | | PF07883 | | Cupin Domain | |
| AAur_1936 | cupin domain protein, putative | | | *--* | -- | | PF07883 | | Cupin Domain | |
| AAur_2355 | cupin domain protein, putative | | | *--* | -- | | PF07883 | | Cupin Domain | |
| AAur_3146 | cupin domain protein, putative | | | *--* | -- | | PF07883 | | Cupin Domain | |
| AAur_3409 | putative cupin domain protein | | | *--* | -- | | PF07883 | | Cupin domain (2 domains) | |
|  |  | | |  |  | | PF05899 | | Protein of unknown function (DUF861) | |
| AAur_3964 | Put. Helix-turn-helix domain | | | *--* | -- | | PF07883 | | Cupin Domain | |
| AAur_4032 | cupin domain protein, putative | | | *--* | -- | | PF07883 | | Cupin Domain | |
| AAur_2822 | Put. pirin-like protein | | | *--* | -- | | PF05726 | | Pirin C-terminal region | |
|  |  | | |  |  | | PF02678 | | Uncharacterized BCR, YhhW family COG1741 | |
| AAur_pTC10055 | cupin domain protein, putative | | | *--* | -- | | PF07883 | | Cupin Domain | |
| AAur_pTC20129 | cupin domain protein, putative | | | *--* | -- | | PF07883 | | Cupin Domain | |
| **Universal stress-related proteins (USPs), heat- and cold-shock proteins:** | | | | | | | | | | |
| AAur_0044 | put universal stress family domain | | | *--* | -- | | PF00582 | | universal stress protein family (2 domains) | |
| AAur_0235 | Put universal stress family domain | | | *--* | -- | | PF00582 | | universal stress protein family | |
| AAur_0410 | Put. universal stress family domain | | | *--* | -- | | PF00582 | | universal stress protein family (2 domains) | |
| AAur_0506 | Put. universal stress family domain | | | *--* | -- | | PF00582 | | universal stress protein family (2 domains) | |
| AAur_0701 | Put. universal stress family domain | | | *--* | -- | | PF00582 | | universal stress protein family | |
| AAur_1001 | chaperonin GroEL | | | *groL* | -- | | TIGR02348 | | chaperonin GroL | |
|  |  | | |  |  | | PF00118 | | TCP-1/cpn60 chaperonin family | |
| AAur_1876 | chaperone protein DnaK | | | *dnaK* | -- | | PF00012 | | DnaK family protein | |
|  |  | | |  |  | | TIGR02350 | | chaperone protein DnaK | |
| AAur_1877 | co-chaperone GrpE | | | *grpE* | -- | | PF01025 | | co-chaperone GrpE | |
| AAur_1878 | putative DnaJ domain protein | | | *--* | -- | | PF00226 | | DnaJ domain | |
|  |  | | |  |  | | PF01556 | | DnaJ C terminal region | |
| AAur_1879 | putative HspR protein homolog | | | *--* | -- | | -- | | -- | |
| AAur_1880 | ATP-dependent chaperone protein | | | *clpB* | -- | | PF00004 | | ATPase, AAA family | |
|  |  | | |  |  | | PF02861 | | Clp amino terminal domain | |
|  |  | | |  |  | | PF02861 | | Clp amino terminal domain | |
|  |  | | |  |  | | PF07728 | | ATPase family associated cell activities (AAA) | |
|  |  | | |  |  | | PF07724 | | ATPase family associated cell activities (AAA) | |
| AAur_2837 | Put. universal stress family domain | | | *--* | -- | | PF00582 | | universal stress protein family | |
| AAur_2874 | chaperonin GroEL | | | *groL* | -- | | TIGR02348 | | chaperonin GroL | |
|  |  | | |  |  | | PF00118 | | TCP-1/cpn60 chaperonin family | |
| AAur_2875 | chaperonin groES | | | *groES* | -- | | PF00166 | | chaperonin GroS | |
| AAur_3886 | putative universal stress family | | | *--* | -- | | PF00582 | | universal stress protein family | |
| AAur_4058 | putative universal stress family | | | *--* | -- | | PF00582 | | universal stress protein family | |
